# Identification of Histone Peptide Binding Specificity and Small-Molecule Ligands for the TRIM33α and TRIM33β Bromodomains

**DOI:** 10.1021/acschembio.2c00266

**Published:** 2022-09-13

**Authors:** Angelina R. Sekirnik, Jessica K. Reynolds, Larissa See, Joseph P. Bluck, Amy R. Scorah, Cynthia Tallant, Bernadette Lee, Katarzyna B. Leszczynska, Rachel L. Grimley, R. Ian Storer, Marta Malattia, Sara Crespillo, Sofia Caria, Stephanie Duclos, Ester M. Hammond, Stefan Knapp, Garrett M. Morris, Fernanda Duarte, Philip C. Biggin, Stuart J. Conway

**Affiliations:** 1 Department of Chemistry, Chemistry Research Laboratory, University of Oxford, Mansfield Road, Oxford OX1 3TA, U.K.; 2 Department of Biochemistry, University of Oxford, South Parks Road, Oxford OX1 3QU, U.K.; 3 Nuffield Department of Clinical Medicine, Structural Genomics Consortium, University of Oxford, Old Road Campus Research Building, Roosevelt Drive, Oxford OX3 3TA, U.K.; 4 Oxford Institute for Radiation Oncology, Department of Oncology, University of Oxford, Old Road Campus Research Building, Oxford OX3 7DQ, U.K.; ⊥ Worldwide Medicinal Chemistry, Discovery Biology, Pfizer Ltd, The Portway, Granta Park, Cambridge CB21 6GS, U.K.; 6 Evotec (UK) Ltd, 90 Park Drive, Milton Park, Abingdon, Oxfordshire OX14 4RZ, U.K.; 7 Institute of Pharmaceutical Chemistry, Goethe University, Max-von-Laue-Strasse 9, D-60438 Frankfurt am Main, Germany; 8 Structural Genomics Consortium, Buchmann Institute for Life Sciences (BMLS), Goethe University, Max-von-Laue-Strasse 15, D-60438 Frankfurt am Main, Germany; 9 Department of Statistics, University of Oxford, 24-29 St Giles’, Oxford OX1 3LB, U.K.

## Abstract

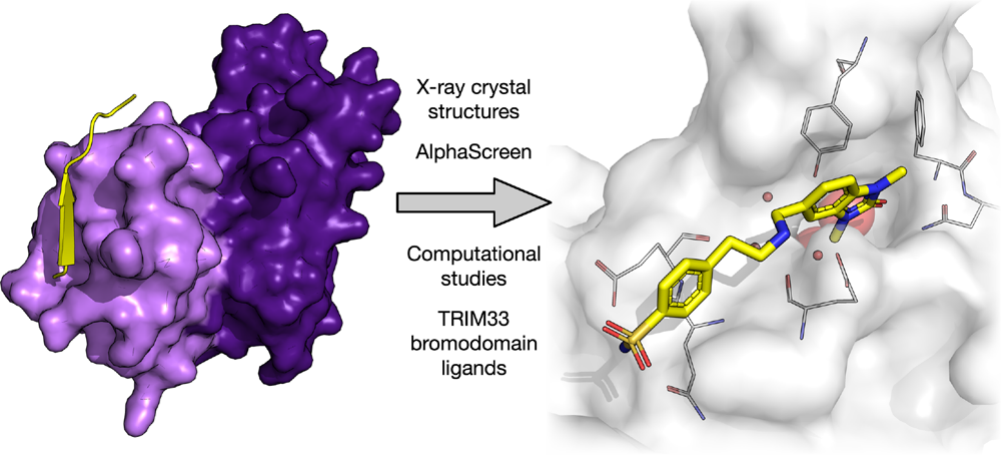

TRIM33 is a member of the tripartite motif (TRIM) family of proteins, some of which possess E3 ligase activity and are involved in the ubiquitin-dependent degradation of proteins. Four of the TRIM family proteins, TRIM24 (TIF1α), TRIM28 (TIF1β), TRIM33 (TIF1γ) and TRIM66, contain C-terminal plant homeodomain (PHD) and bromodomain (BRD) modules, which bind to methylated lysine (KMe_*n*_) and acetylated lysine (KAc), respectively. Here we investigate the differences between the two isoforms of TRIM33, TRIM33α and TRIM33β, using structural and biophysical approaches. We show that the N1039 residue, which is equivalent to N140 in BRD4(1) and which is conserved in most BRDs, has a different orientation in each isoform. In TRIM33β, this residue coordinates KAc, but this is not the case in TRIM33α. Despite these differences, both isoforms show similar affinities for H3_1–27_K18Ac, and bind preferentially to H3_1–27_K9Me_3_K18Ac. We used this information to develop an AlphaScreen assay, with which we have identified four new ligands for the TRIM33 PHD-BRD cassette. These findings provide fundamental new information regarding which histone marks are recognized by both isoforms of TRIM33 and suggest starting points for the development of chemical probes to investigate the cellular function of TRIM33.

## Introduction

**Figure 1:**
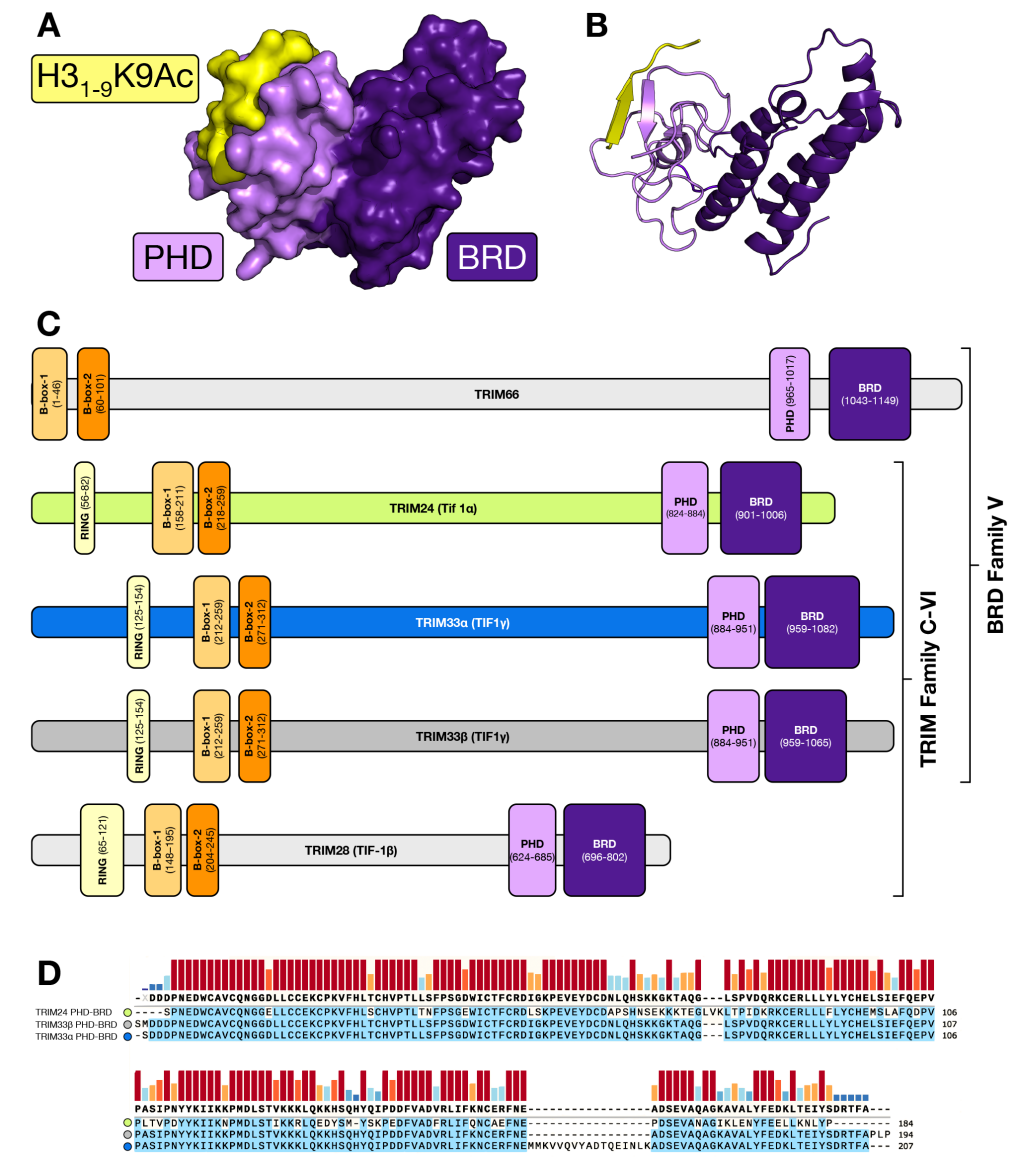
X-ray crystal structure of the PHD-BRD cassette of TRIM33β bound to H3_1–9_K9Ac (PDB code 5MR8) with (A) the surface represented and (B) the cartoon showing the extended β-sheet formed between the PHD and H3_1–9_K9Ac. Figures were generated using the PyMOL Molecular Graphics System, version 2.5, Schrödinger, LLC. (C) Domain composition of TRIM24, TRIM28, TRIM33, and TRIM66. The PHD (lilac) and BRD (purple) domains of TRIM proteins sit at the C-terminus of the proteins. The TRIM family C–VI proteins (TRIM24, TRIM28, and TRIM33) and the homologous TRIM66 possess a tandem PHD-BRD cassette. TRIM24, TRIM33, and TRIM66 are in BRD Family V. Figure generated using OmniGraffle. (D) TRIM24 PHD-BRD has 62.0% identity and 75.5% similarity with TRIM33α. TRIM24 PHD-BRD has 67.8% identity and 82.5% similarity with TRIM33β. TRIM33α has 91.8% identity and 91.8% similarity with TRIM33β; they are identical apart from the 17 amino acid insertion/deletion. The primary sequences were obtained from PDB files 3U5O (TRIM33α), 4YC9 (TRIM24), and 5MR8 (TRIM33β). Local alignment is Smith–Waterman using SnapGene.

TRIM33 is a member of the tripartite motif (TRIM) family of proteins, which are characterized by an N-terminal tripartite motif typically containing one RING-finger domain, one or two zinc-finger domains (B1 box and B2 box), and an associated coiled-coil region. Most TRIM proteins possess E3 ligase activity and are involved in the ubiquitin-dependent degradation of a number of important proteins.^1,2^ Four of the TRIM family proteins, TRIM24 (TIF1α), TRIM28 (TIF1β), TRIM33 (TIF1γ), and TRIM66, possess C-terminal plant homeodomain (PHD) and bromodomain (BRD) modules (Figure 1A–C), which bind to methylated lysine (KMe_*n*_) and acetylated lysine (KAc), respectively.

PHDs are small, independently folded, 50–80 residue long protein domains. Over 170 sequences have been annotated as PHD fingers in the human genome.^3^ They contain conserved regions of cysteine and histidine residues that coordinate two Zn^2+^ ions in a cross braced fashion, with an antiparallel two-stranded β-sheet core, which imposes conformational stability.^3−7^ These domains read a range of histone marks, notably the methylation state of the K4 residue of H3 histone (H3K4Me_3_, H3K4Me_2_, or H3K4Me_0_) and the H3K9 and H3R2 positions.^8,9^ Mutations within this motif have been linked to immunological, neurological, and developmental disorders.^9,10^

BRDs are well characterized protein modules comprising approximately 110 amino acids that bind to KAc residues on histones and many other proteins.^11−15^ Their well-defined tertiary structure comprises a left-handed, antiparallel four-helical bundle (αA, αB, αC, and αZ),^16^ which is structurally conserved across the family, despite relatively low sequence identity.^17^ The KAc binding pocket of most BRDs is hydrophobic but contains structural water molecules at the base of the pocket and, in some cases, in the ZA channel.^18^ The ability of these water molecules to be displaced varies between classes of BRDs and has been exploited to develop selective ligands for certain proteins.^19,20^ The canonical BRDs possess a highly conserved asparagine residue, which forms a hydrogen bond to KAc. However, 13 non-canonical BRDs exist, 12 of which possess threonine or tyrosine residues at this position that are, in principle, capable of hydrogen-bonding to KAc.^15^ One of these is TRIM28, in which the asparagine residue is replaced by a threonine.^15^ In addition, the 17-amino acid insertion in TRIM33α (see below) moves this asparagine out of the KAc-binding pocket, resulting in no obvious residue that can interact with KAc at this position. The inherent affinity of BRDs for a single KAc amino acid is low, and further affinity is derived from interactions with the cognate peptide/protein, which also confers selectivity for given acetylated binding partners.^21^ These PHD and BRD modules enable chromatin binding of proteins, and consistent with this, deletion of either the BRD or the PHD in TRIM33 prevents its localization to sites of DNA breaks.^22^

The biological functions of TRIM24, TRIM28, TRIM33, and TRIM66 have been investigated, and some key findings are briefly described below. Overexpression of TRIM24 is connected to tumor progression and poor prognosis in breast cancer,^23^ and significant upregulation has been observed in cancers including gastric cancer,^24^ non-small-cell lung cancer,^25^ leukemia,^26^ prostate cancer,^27^ and hepatocellular carcinoma.^28^ Knockdown of TRIM24 in colon cancer cell lines suppressed tumor growth and induced apoptosis.^29^ Despite mouse models indicating that TRIM24 can act as a liver-specific tumor suppressor,^30^ most studies show that TRIM24 is an oncogene when overexpressed.

TRIM33 is a tumor suppressor in breast cancer,^31^ non-small-cell lung cancer,^32^ and clear cell renal cell carcinoma^33^ and, through its role in β-catenin degradation, prevents brain tumor development and human glioblastoma.^34^ TRIM33 also has a role in regulation of the transforming growth factor beta (TGF-β) superfamily.^35,36^ In contrast, TRIM33 can also function as a tumor promoter by preventing apoptosis in B lymphoblastic leukemia,^37^ demonstrating that TRIM33 has a range of biological functions.^38^ TRIM33 also plays a role in the poly(ADP-ribose) polymerase (PARP)-dependent DNA damage response pathway.^22^ TRIM66 has also recently been shown to act in the DNA damage response and binds to H3R2K4 and H3K56Ac.^39^ Prior to their identification,^40^ biological studies did not distinguish between the two TRIM33 isoforms, TRIM33α and TRIM33β.

The location of two reader domains proximal to each other in TRIM24, TRIM28, TRIM33, and TRIM66 raised the question of whether and how these domains function together. It is possible that binding of a target protein to one domain will result in a different biological response to binding to the other domain or both domains simultaneously.

The H3 histone modifications recognized by TRIM24, TRIM28, TRIM33, and TRIM66 have been the subject of a number of studies. The targets of the TRIM24 reader domains were identified by Tsai et al., reporting TRIM24 as a dual reader of unmodified H3K4 and H3K23Ac.^23^ Xi et al. have characterized TRIM33α, the full-length isoform of TRIM33. The PHD of TRIM33α binds to unmodified H3K4 and H3K9Me_3_ through an interaction with W889.^36^ This residue is conserved in TRIM24, where binding to H3K9Me_3_ has been reported but not quantified.^41^ The BRD of TRIM33α binds to H3K18Ac, which is an appropriate distance from H3K9 to be read simultaneously. A recent study by Chen et al. demonstrated that all peptides binding to the PHD of TRIM24, TRIM33, or TRIM66 require unmodified H3R2.^39^

The study by Xi et al. focused on the TRIM33α isoform, which contains a BRD with a 17-amino acid insertion on the BC loop, compared to the TRIM24 sequence.^36^ However, a second isoform of TRIM33 has been identified, TRIM33β,^40^ which is a splice variant that lacks the 17-amino acid insertion and is homologous to TRIM24 (Figure 1D). The histone H3 binding profile of TRIM33β has not previously been compared to TRIM33α, and the role of the extended BC loop in TRIM33α has not been explored. The X-ray crystal structures of TRIM33α show a non-canonical BRD, in which the conserved N1039 residue is located outside of the KAc-binding pocket, indicating that it cannot interact with KAc,^36^ and no residue replaces N1039 to compensate. Additionally, prior to this work there was no structural information on the TRIM33β isoform to demonstrate the location of the N1039 residue.

Over the past decade, BRDs have emerged as a ligandable class of protein modules that are therapeutically relevant.^12,13,18,42−45^ Despite this, the TRIM proteins remain among the most understudied BRD-containing targets. Ligands for the BRD of TRIM24, which also bind to the BRD of BRPF1, have been reported by both Palmer et al.^46,47^ and Bennett et al.^48^ In addition, a PROTAC for TRIM24 was developed and confirmed TRIM24 as a novel dependency in acute leukemia.^26^ At the start of this work, there were no reported small molecule ligands for either isoform of TRIM33. In a recent patent, multiple putative TRIM33α ligands were disclosed;^49^ there are no non-peptide ligands reported for the PHDs of TRIM24 or TRIM33.

Here we report an analysis of the histone H3 peptide binding profile of TRIM24, TRIM33α, and TRIM33β. We show that both TRIM33α and TRIM33β bind preferentially to H3_1–27_K9Me_3_K18Ac. Surprisingly, we found that TRIM33α and TRIM33β have similar binding affinities for H3_1–27_K18Ac, despite the non-canonical BRD of TRIM33α. We also show that the PHD and BRD of these proteins contribute equally to the affinity for the dual modified H3_1–27_K9Me_3_K18Ac peptide and that this contribution is additive. We have employed this information to develop an AlphaScreen assay^50,51^ for the tandem PHD-BRD cassettes of TRIM24, TRIM33α, and TRIM33β. Using these assays, we screened approximately 1700 compounds, and identified novel ligands for TRIM24, TRIM33α, and TRIM33β. We also show that the compounds disclosed in a recent patent^49^ do not bind to TRIM33β in our hands. This work provides the foundation for the development of more refined TRIM33 ligands, which will enable the function of this fascinating protein to be further explored and potentially recruited as an E3 ligase in proteolysis-targeting chimeras (PROTACs).

## Results and Discussion

### TRIM33 Has Higher Expression Levels in Cancer Cell Lines Compared to Noncancer Cell Lines

**Figure 2:**
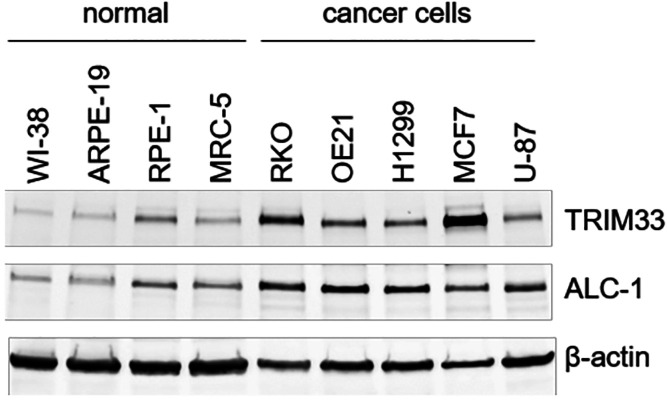
Western blot analysis of expression levels in cancer and noncancer cell lines show that both TRIM33 and amplified in liver cancer 1 (ALC1 or CHD1L) are generally present in higher levels in the cancer cell lines. Noncancerous cell lines: WI-38 = lung fibroblasts; ARPE-19 = retinal pigment epithelial cells; RPE-1 = retinal pigment epithelial cells; MRC-5 = lung. Cancer cell lines: RKO = colon carcinoma; OE21 = esophageal squamous cell carcinoma; H1299 = non-small-cell lung carcinoma; MCF7 = breast adenocarcinoma; U-87 = glioblastoma.

To determine whether TRIM33 is of potential interest as a therapeutic target in oncology, we compared the expression levels of TRIM33 in a range of cancer and noncancer cell lines using Western blotting (Figure 2). We demonstrate that the cancer cell lines generally show higher expression levels of both TRIM33 and amplified in liver cancer 1 (ALC1 or CHD1L) compared to related noncancer cell lines. ALC1 is a helicase that is recruited to the site of single strand breaks (SSBs) through binding of its macrodomain to poly(ADP-ribose) (PAR). Its helicase activity relaxes the chromatin structure to allow repair, but this activity is short-lived (*t*_1/2_ ≈ 2.5 min) and tightly regulated as prolonged chromatin relaxation exposes it to further damage.^52^ TRIM33 is recruited to the site of SSBs in an ALC1-dependent manner and is required to ensure timely dissociation of ALC1 from chromatin.^22^ TRIM33 does not bind PAR directly, but deletion of the chromatin-binding PHD/BRD module prevents TRIM33 localization to sites of laser scissor-induced DNA breaks. TRIM33 (sh/siRNA) knock down studies conducted by Kulkarni et al.^22^ showed accumulation of ALC1 at the sites of DNA damage, evidence of DNA damage-induced checkpoint activation, and prolonged DNA damage. Combined with our data, these observations raise the possibility that inhibition of TRIM33 BRD or PHD function or both might be of therapeutic benefit in cancers that have high levels of TRIM33. These data encouraged us to identify ligands for the TRIM33 BRD and PHD to probe their functions.

### TRIM33β Contains a Canonical Bromodomain

**Figure 3:**
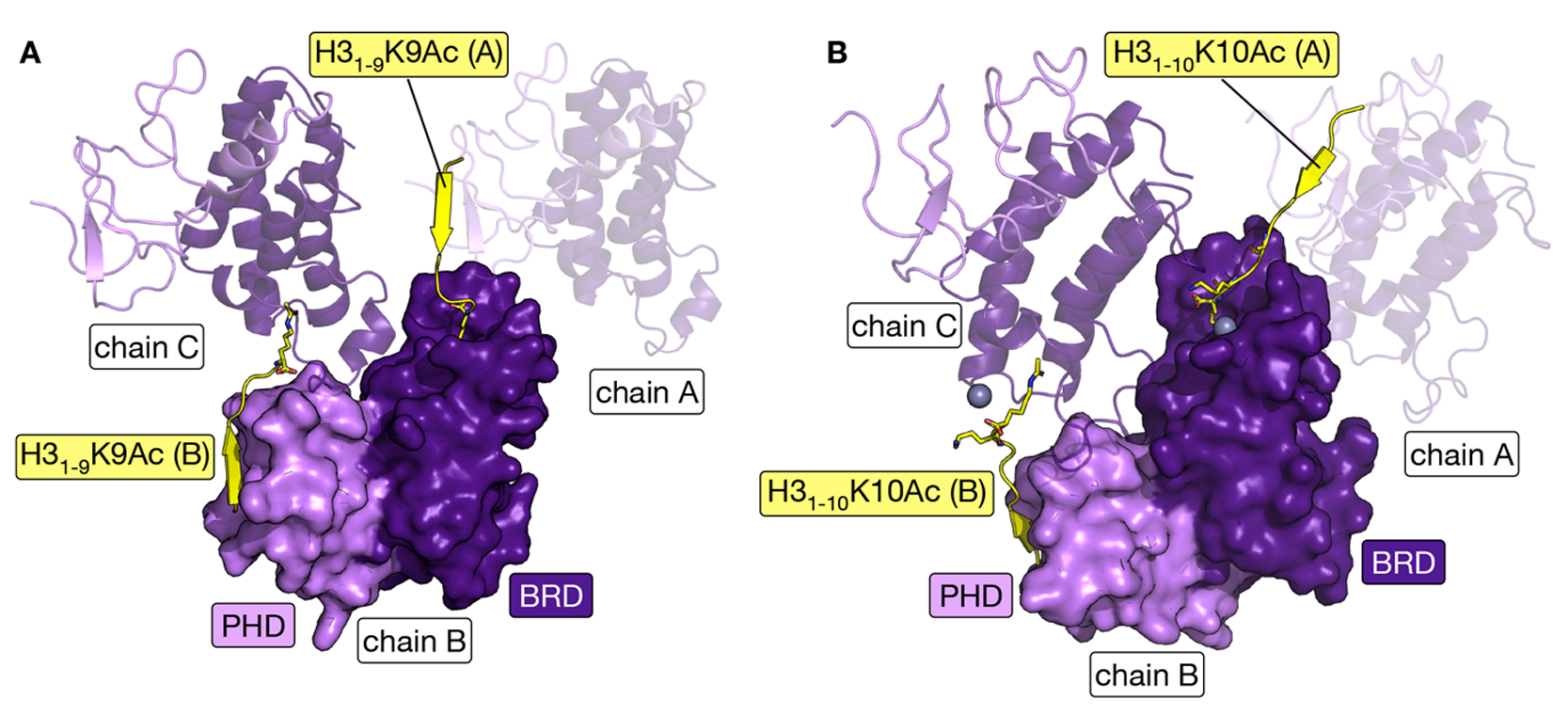
X-ray crystal structures of TRIM33β bound to (A) H3_1–9_K9Ac (PDB code 5MR8) or (B) H3_1–10_K10Ac (PDB code 7ZDD). Two symmetry mates are shown (represented as cartoon) revealing that, in both cases, the H3-mimicking peptide binds to the PHD of one chain and the KAc-binding pocket of an adjacent protein (i.e., in *trans*). Figures generated using the PyMOL Molecular Graphics System, version 2.5, Schrödinger, LLC.

We were interested to investigate the effect of the 17-amino acid deletion observed in TRIM33β on its BRD structure, and whether this alteration affected the orientation of N1039 in relation to the KAc binding pocket. To achieve this, we sought to obtain X-ray crystal structures of the TRIM33β BRD and PHD cassette, as X-ray structural data have only previously been reported for TRIM33α. The TRIM33β cassette (residues 888–1127) was expressed and cocrystallized with the PHD-binding region of the H3 peptide (H3_1–9_K9Ac) (PDB 5MR8). We also obtained an X-ray crystal structure of 7ZDD construct bound to a peptide that does not occur naturally, H3_1–10_K10Ac, which possesses the first 9 residues of the H3 peptide (K9 unmodified) with a KAc residue added as the 10th residue. This peptide was ordered in error, but the X-ray crystal structure with it bound still provides useful structural information and so we have included it here. Both 5MR8 and 7ZDD show the TRIM33β PHD-BRD cassette crystallizing with the H3-mimicking peptide bound to the PHD of one protein chain, through residues 1–8, with the K9Ac or K10Ac residue occupying the KAc binding pocket of an adjacent protein chain (Figure 3A,B).

**Figure 4:**
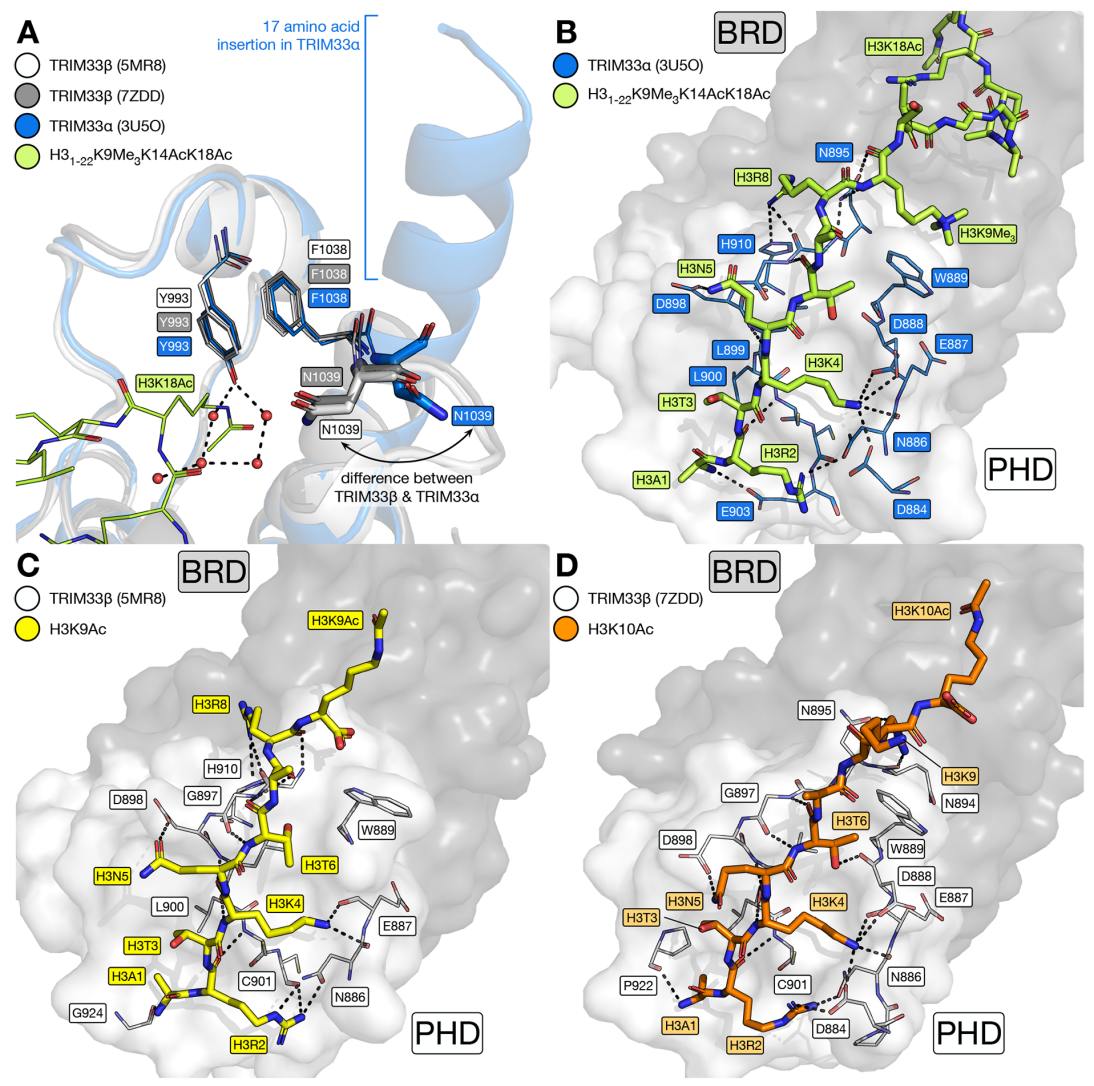
X-ray crystal structures of the TRIM33α and -β BRD and PHD cassettes. (A) Overlay of the X-ray crystal structures of the TRIM33α BRD-PHD (carbon = blue, PDB code 3U5O) in complex with the histone H3-mimicking peptide H3_1–22_K9Me_3_K14AcK18Ac (carbon = lime, PDB code 3U5O),^36^ and the TRIM33β PHD-BRD (PDB codes 5MR8 and 7ZDD). Both X-ray crystal structures of TRIM33β show that the BRD is canonical, with N1039 residing inside the KAc-binding pocket, while this residue sits outside of the pocket in TRIM33α. The 17 amino acid extension present in TRIM33α is shown as a blue helix cartoon. (B) H3_1–22_K9Me_3_K14AcK18Ac (carbon = lime, PDB code 3U5O) peptide forms a number of backbone interactions resulting in an extended β-sheet structure with the PHD of TRIM33α (carbon = blue, PDB code 3U5O). H3R2 interacts with N886 and H3K4 forms interactions with D884, E887, D888, and the backbone carbonyl oxygen of N886. The side chains of H3N5 and H3R8 also interact with the TRIM33α PHD. H3K9Me_3_ is observed in proximity to W889, implying the existence of a cation−π interaction. Both the H3_1–9_K9Ac (C; carbon = yellow) and H3_1–10_K10Ac (D; carbon = orange) peptides form similar interactions with the TRIM33β PHD to those formed by H3_1–22_K9Me_3_K14AcK18Ac with the TRIM33α PHD. Figures generated using the PyMOL Molecular Graphics System, version 2.5, Schrödinger, LLC.

Both X-ray crystal structures show that the BRD of TRIM33β is canonical, with N1039 residing inside the KAc-binding pocket, unlike TRIM33α (Figure 4A), where the residue is located 6.2 Å away (N1039 NH_2_ to NH_2_ distance) outside of the pocket. The H3-mimicking peptides form very similar interactions with TRIM33β to those formed with TRIM33α (Figure 4B–D). The peptide acts as an extended β-sheet, with a significant number of backbone interactions observed. The necessity of unmodified H3R2 for effective binding is reflected by this residue forming interactions with a number of residues; the interaction with N886 is present in all structures. The same is true for unmodified H3K4, which interacts with D884, E887, D888, and the backbone carbonyl oxygen of N886. H3N5 hydrogen bonds to D898, and H3R8 forms interactions with H910 (Figure 4B–D). The KAc residues do not form any interactions with the protein in *cis* (i.e., to the same protein), as they are bound to the BRD of an adjacent protein copy in *trans* (see above).

### The TRIM33β PHD-BRD Domains Bind to Histone H3 K9Me_3_ and K18Ac Marks

To establish an AlphaScreen assay,^13,50,51^ the PHD-BRD cassettes of TRIM24 and both TRIM33 isoforms were expressed with an N-terminal His_6_ tag attached (CD spectra for these constructs are shown in Figure S9). Consistent with the X-ray crystallography data above, it has been established previously that methylation of the K4 position of histone H3 reduces affinity to the PHD finger: *K*_d_ values for the TRIM24 PHD have been measured by ITC as 8.6 μM for H3_1–15_K4Me_0_, 41 μM for H3_1–15_K4Me_1_, 198 μM for H3_1–15_K4Me_2_, and >400 μM for H3_1–15_K4Me_3_.^53^ The presence of the four N-terminal residues of the peptide (H3_1–4_), which are recognized by the β-sheet surface of the PHD finger, are essential for affinity to TRIM33α.^35^ Consequently, we maintained K4Me_0_ in most of the peptides investigated, to avoid affinities falling below detection limits. The peptide recognition profiles of TRIM24, TRIM33α, and TRIM33β were investigated by determining affinity of their tandem BRD-PHD cassettes for H3-mimicking peptides containing combinations of acetylation at K14, K18, or K23, and methylation of K9 (Table 1).

**Table 1:**
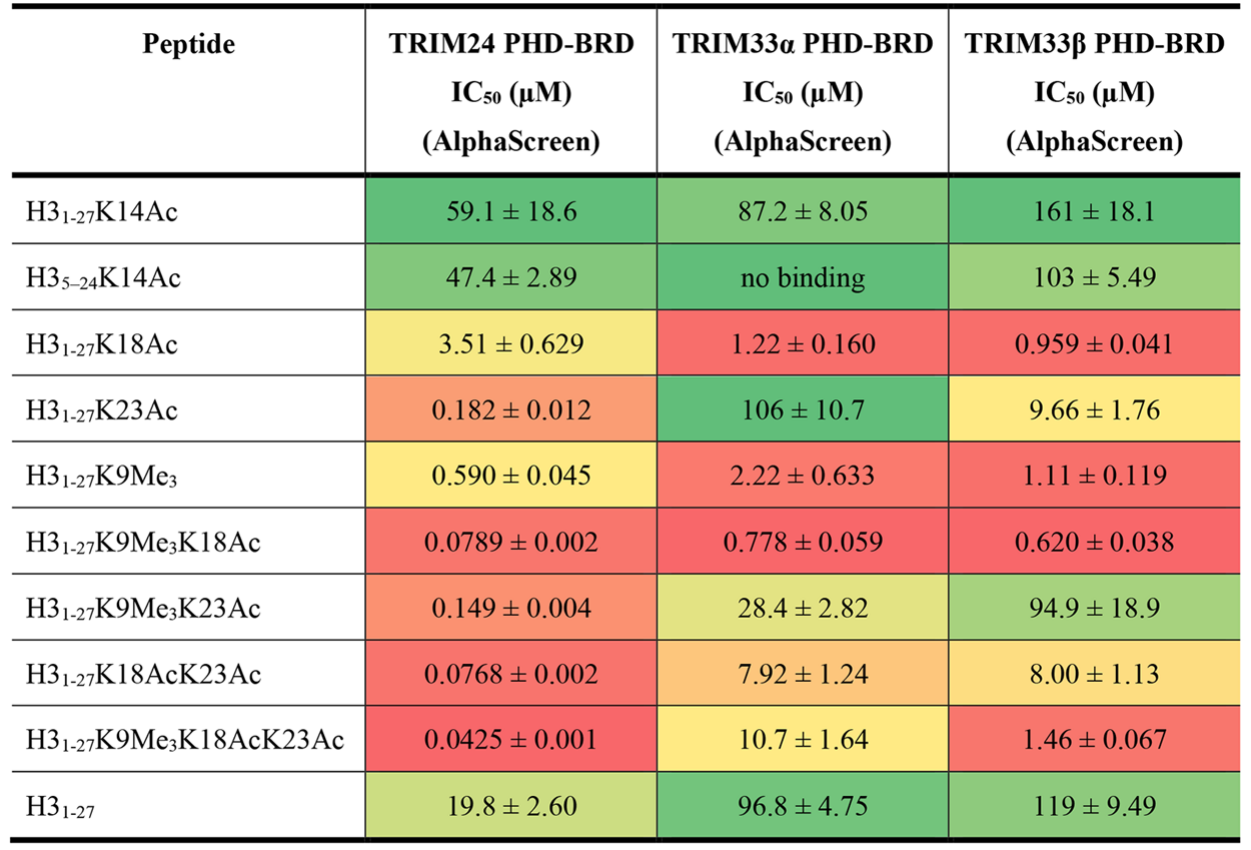
AlphaScreen IC_50_ Values for Modified Histone H3-Based Peptides Binding to the PHD-BRD Cassette of TRIM24, TRIM33α, or TRIM33β^a^

a Dose-response of unbiotinylated peptide displacing the biotinylated equivalent from the protein. Values quoted are the mean of triplicate data ± standard error of the mean. A heatmap display is used with “hot” colors corresponding to lower *K*_d_ values. See Tables S14 and S15 for peptide concentration and sequences.

The data from the AlphaScreen assay show that the TRIM24 PHD-BRD binds preferentially to H3_1–27_K23Ac over the other monoacetylated peptides, while both TRIM33α and TRIM33β PHD-BRD bind preferentially to H3_1–27_K18Ac, which is consistent with the literature (Table 1).^36,53^ We note that the dual modified peptide H3_1–27_K18AcK23Ac shows increased affinity for the TRIM24 PHD-BRD (IC_50_ = 0.0768 ± 0.002 μM vs 0.182 ± 0.012 μM for H3_1–27_K18Ac). Conversely, H3_1–27_K18AcK23Ac has lower affinity for both TRIM33α (IC_50_ = 7.92 ± 1.24 μM vs 1.22 ± 0.160 μM for H3_1–27_K18Ac) and TRIM33β (IC_50_ = 8.00 ± 1.13 vs 0.959 ± 0.041 μM for H3_1–27_K18Ac) PHD-BRD. It is interesting that the TRIM33α PHD-BRD and TRIM33β PHD-BRD show the same affinity for H3_1–27_K18Ac, despite the difference in orientation of N1039 in these constructs.

All three proteins bind to H3_1–27_K9Me_3_, with the TRIM24 PHD-BRD showing approximately double the affinity (IC_50_ = 0.590 ± 0.045 μM) compared to the TRIM33α (IC_50_ = 2.22 ± 0.633 μM) or TRIM33β PHD-BRD (IC_50_ = 1.11 ± 0.119 μM). Despite their different sequence preferences for the monoacetylated peptides, all three constructs show preferential binding to the dual modified H3_1–27_K9Me_3_K18Ac peptide, compared to H3_1–27_K9Me_3_K23Ac peptide (Table 1). The structural data indicate that K9Me_3_ and K18Ac are optimally spaced to allow simultaneous binding to the PHD and BRD in *cis*, resulting in higher affinity compared to H3_1–27_K9Me_3_K23Ac. The TRIM33α and TRIM33β PHD-BRD have the highest affinity for the H3_1–27_K9Me_3_K18Ac peptide; additional modifications to the peptide all result in reduced affinity. Interestingly, the dual modified H3_1–27_K18AcK23Ac peptide shows similar affinity (IC_50_ = 0.0768 ± 0.002 μM) for the TRIM24 PHD-BRD to the H3_1–27_K9Me_3_K18Ac peptide (IC_50_ = 0.0789 ± 0.002 μM). The TRIM24 PHD-BRD shows the highest affinity for the H3_1–27_K9Me_3_K18AcK23Ac peptide (IC_50_ = 0.0425 ± 0.001 μM).

To confirm the data obtained using our AlphaScreen assay, we investigated the affinity of the H3_1–27_K9Me_3_, H3_1–27_K18Ac, and H3_1–27_K9Me_3_K18Ac peptides for the TRIM24, TRIM33α, and TRIM33β PHD-BRD using ITC (Table 2). The data obtained show similar trends to the AlphaScreen data. The TRIM24 PHD-BRD shows similar affinity for H3_1–27_K9Me_3_ (*K*_d_ = 3.90 ± 0.423 μM) and H3_1–27_K18Ac (*K*_d_ = 4.37 ± 0.430 μM). The affinity of the two marks is additive, with H3_1–27_K9Me_3_K18Ac having higher affinity (*K*_d_ = 1.94 ± 0.111 μM). This observation is in line with work from Ruthenburg et al., who showed that binding of H3K4Me_3_ and H4K16Ac (in *trans*) is cooperative.^54^ The TRIM33α and TRIM33β PHD-BRD cassettes show very similar affinity for H3_1–27_K9Me_3_ (*K*_d_ = 11.2 ± 0.760 μM and 9.81 ± 2.33 μM, respectively) and H3_1–27_K18Ac (*K*_d_ = 10.0 ± 0.319 μM and 8.95 ± 0.643 μM, respectively). Interestingly the affinity of these two marks is not additive for TRIM33α as the dual modified H3_1–27_K9Me_3_K18Ac peptide shows a similar *K*_d_ value to the singly modified peptides (*K*_d_ = 9.28 ± 0.214 μM). The dual modified H3_1–27_K9Me_3_K18Ac peptide has approximately double the affinity for the TRIM33β PHD-BRD (*K*_d_ = 4.78 ± 0.200 μM), compared to the singly modified peptides. The ITC N values from TRIM24 and TRIM33 range from 0.868 to 0.939 for the dual modified H3_1–27_K9Me_3_K18Ac peptide, indicating that the dual marked peptide is binding in *cis* in under these conditions (Table S18). We note that these absolute *K*_d_ values observed using ITC are weaker than the IC_50_ values observed using AlphaScreen, which we attribute to the competitive nature and inherent variability in the AlphaScreen format,^50^ although the rank order of affinities is meaningful. The values observed using ITC are in line with those seen for other BRD–histone peptide interactions.^15^

**Table 2:**
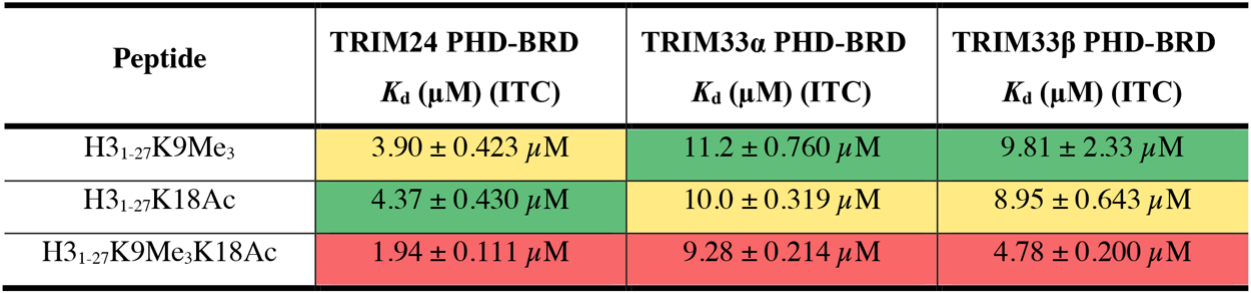
ITC *K*_d_ Values for Modified Histone H3-Based Peptides Binding to the PHD-BRD Cassette of TRIM24, TRIM33α, or TRIM33β^a^

a Values quoted from a representative run ± error of the curve fit. H3-peptide was injected (20 × 2 μL injections) into a cell containing protein in 50 mM HEPES buffer, pH 7.4, 150 mM NaCl. Raw heat effects for the data are shown in the Supporting Information. A heatmap display is used with “hot” colors corresponding to lower *K*_d_ values.

### Mutation Studies Identify the Contribution of Key Residues for H3 Binding

Having established the H3_1–27_K9Me_3_, H3_1–27_K18Ac, and H3_1–27_K9Me_3_K18Ac peptides as binding partners for the TRIM33α and TRIM33β PHD-BRD, we next sought to investigate the contributions of key residues in the PHD and BRD to peptide recognition. To achieve this, we used the signal response curves for mutants of the TRIM24, TRIM33α, and TRIM33β PHD-BRD, and biotinylated peptides in the AlphaScreen assay. To probe binding to the PHD, we made the W828A mutation in TRIM24, and the W889A mutation in TRIM33α and -β. To probe BRD binding, we made both the N980A and N980F mutants in TRIM24 and the N1039F mutation in TRIM33α and -β. The N to F mutation has previously been shown to be more effective at preventing KAc binding to the BRD than the N to A mutation at the same position.^55^

The W828A and W889A mutants generated no signal in the AlphaScreen assay when the proteins were incubated with varying concentrations of the H3_1–27_K9Me_3_ peptide. This indicates that this mutation abolishes PHD-mediated recognition of KMe_3_ for all three proteins (Table 3). This result confirms the importance of W828/W889 in the recognition of the H3K9Me_3_ modification. The affinity of the dual modified H3_1–27_K9Me_3_K18Ac was also reduced by the W828A/W889A mutations, compared to the wild-type proteins. The largest reduction in signal response for the H3_1–27_K9Me_3_K18Ac peptide occurred for the TRIM33α PHD-BRD, suggesting that the K9Me_3_ modification provides a substantial contribution to the TRIM33α PHD-BRD affinity for the peptide, in line with the ITC data.

**Table 3:**
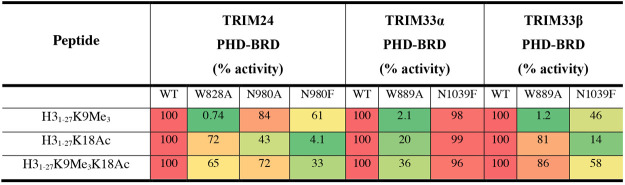
AlphaScreen Signal Response for Biotinylated Peptides with Proteins Bearing Inactive Mutations^a^

a Peptides were serially diluted 1:2. A heatmap display is used, with high percentage activity shown in red and low percentage activity shown in green. High percentage activity indicates binding of the peptide to the protein; reduced percentage activity indicates reduced binding. Percentage activity was calculated using the equation shown in the Supporting Information. Binding curves generated using GraphPad Prism are shown in the Supporting Information.

As expected, the N980F mutant was more effective than N980A at reducing binding of the H3_1–27_K18Ac and H3_1–27_K9Me_3_K18Ac peptides to the TRIM24 PHD-BRD (Table 3). The N1039F mutation also reduced binding of the H3_1–27_K18Ac peptide to the TRIM33β PHD-BRD, which has the canonical BRD. However, this mutation had no effect on the binding of the H3_1–27_K18Ac and H3_1–27_K9Me_3_K18Ac peptides to the TRIM33α PHD-BRD. This result is consistent with the structural data showing that N1039 is located outside of the KAc binding pocket of the BRD. This result is particularly interesting when considered together with the previous AlphaScreen and ITC data (Tables 1 and 2). The ability of the TRIM33α PHD-BRD to bind to the H3_1–27_K18Ac peptide and the fact that this binding is not disrupted by the N1039F mutation indicate that the K18Ac residue does bind in the BRD and that N1039 does not move into the KAc binding pocket to interact with K18Ac. This suggests that K18Ac forms interactions with the TRIM33α BRD that do not involve N1039.

### Molecular Dynamics Simulations of the H3 Peptide Identify Recognition Patterns for H3K18Ac to TRIM24 and the TRIM33 Isoforms

To probe interaction of the H3 mimicking peptides with TRIM24, TRIM33α, and TRIM33β, we conducted molecular dynamics (MD) simulations. Using the X-ray crystal structure of the TRIM33α PHD-BRD bound to H3_1–20_K9Me_3_K14AcK18Ac (PDB code 3U5O),^36^ models of each TRIM reader domain bound to H3_1–20_K9Me_3_K18Ac were generated through alignment and used to perform MD simulations (see SI for details).

**Figure 5:**
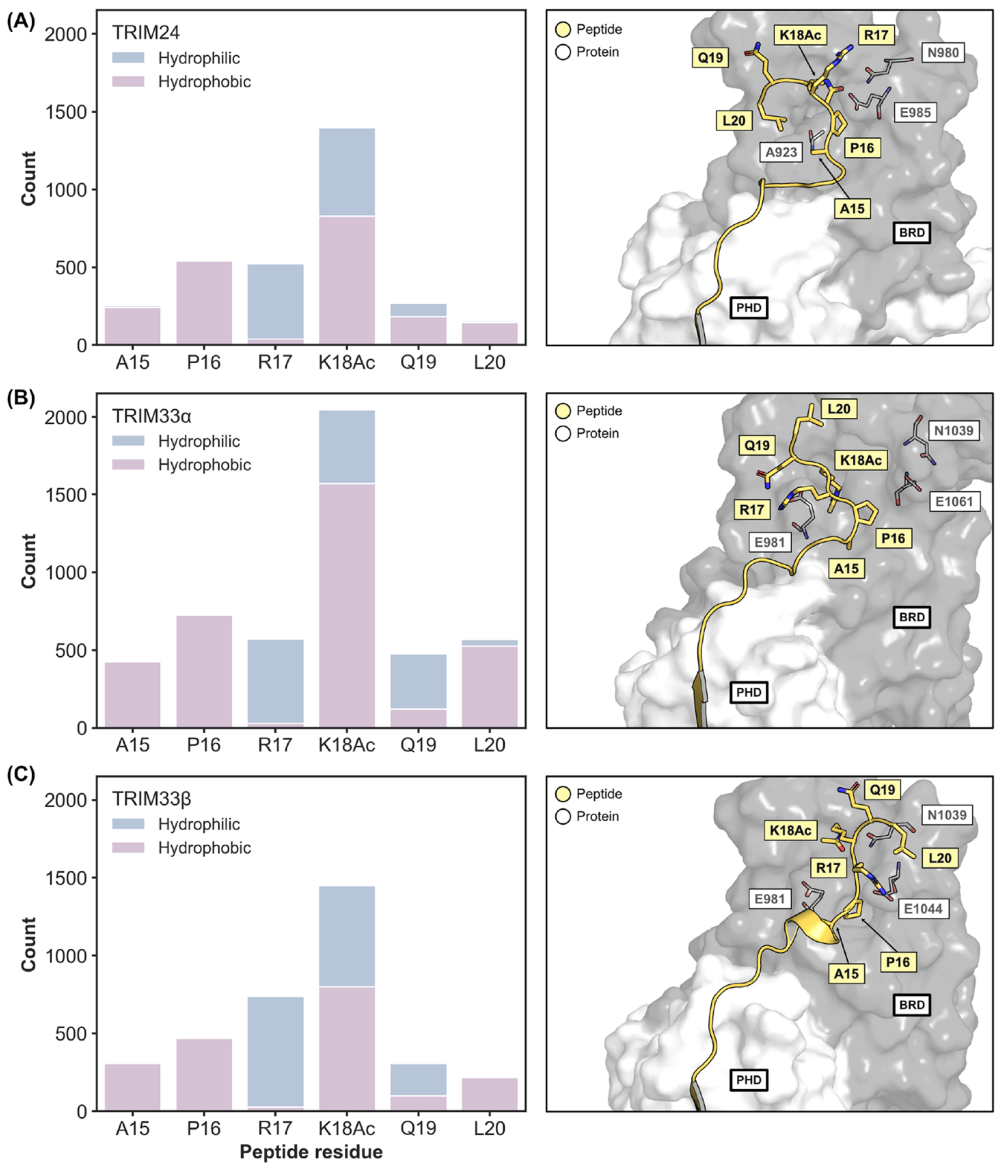
Hydrophobic and hydrophilic contact analysis for (A) TRIM24, (B) TRIM33α, and (C) TRIM33β, showing that H3K18Ac forms more interactions with the BRDs as compared to other peptide residues. Protein–Ligand Interaction Profiler (PLIP) was run on 101 frames, taken 1 ns apart, from each of the 5 independent MD runs, totalling 505 frames for each protein. Beside each bar plot a representative snapshot of the peptide bound to each BRD is shown, which corresponds to the middle structure of the top cluster based on the root-mean-squared deviation (RMSD) of the backbone atoms of peptide residues 15–20. The images of the proteins were made using the PyMOL Molecular Graphics System, version 2.5, Schrödinger, LLC.

The simulations were analyzed using Protein–Ligand Interaction Profiler (PLIP)^56^ to identify hydrophilic and hydrophobic contacts between the TRIM PHD-BRDs and the H3_1–20_K9Me_3_K18Ac peptide. Hydrophilic contacts were defined as hydrogen bonds, salt bridges, and cation−π interactions, while hydrophobic contacts were defined as interactions between close apolar peptide/protein atoms and π-stacking interactions (Figure 5). H3K18Ac made the most contacts with each BRD, consistent with the recognition of this modified residue by BRDs, but the nature of the interaction differed between systems. A similar number of hydrophilic and hydrophobic contacts were identified between the peptide and the TRIM24 or TRIM33β BRDs. Conversely, hydrophobic contacts formed the majority of interactions between H3K18Ac and the TRIM33α BRD.

**Figure 6:**
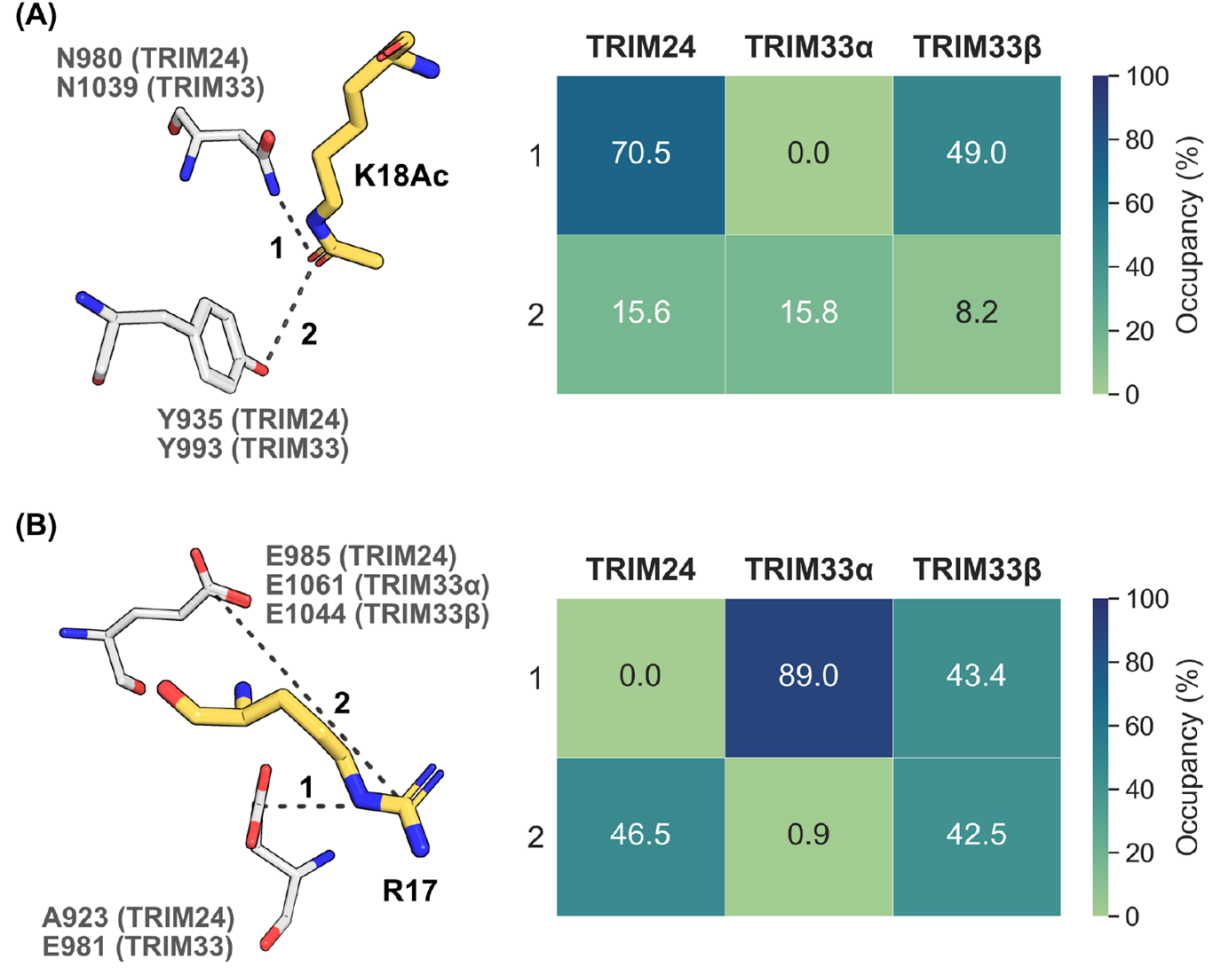
Occupancy of direct hydrophilic interactions (hydrogen bonds/salt bridges) between (A) H3K18Ac or (B) H3R17 and the TRIM BRDs during MD simulations (peptide carbon = yellow, protein carbon = white). Data are taken from 5 × 100 ns MD simulations (see SI for details on how these interactions were detected). The images of the proteins were made using the PyMOL Molecular Graphics System, version 2.5, Schrödinger, LLC.

The hydrophilic contacts between the BRDs and H3K18Ac and the flanking H3R17 residue were examined in greater detail as these two residues were found to display the most hydrophilic contacts with the TRIM-BRDs (Figure 6). In TRIM24 and TRIM33β, H3K18Ac directly interacts with N980/N1039 via the KAc carbonyl group (Figure 6A, Interaction 1), as has been observed for other canonical BRDs. This observation is consistent with experimental data showing that TRIM24 N980F and TRIM33β N1039F mutants have substantially disrupted binding to the H3_1–27_K18Ac peptide (Table 3). In contrast, no direct interactions between H3K18Ac and N1039 in TRIM33α were observed, as this residue remains oriented away from the BRD pocket during simulations (consistent with the orientation shown in Figure 4A). We also examined other hydrogen bond interactions between H3K18Ac and Y935/Y993, but they were only observed intermittently (Figure 6A, Interaction 2). This analysis is also consistent with the TRIM33α crystallographic data (e.g., PDB code 3U5O) indicating that selective binding of both TRIM33 isoforms to H3K18Ac over other KAc residues is guided by an electrostatic interaction between E981 and H3R17 (Figure 6B, Interaction 1).^36^ In addition to the H3R17–E981 interaction, a less common interaction between H3R17 and a glutamate residue on the BC loop of TRIM24 and TRIM33β was also observed (Figure 6B, Interaction 2). This might account for TRIM24 also showing affinity for H3K18Ac despite having A923 in place of E981 on the ZA loop.^36^

Having observed minimal direct hydrogen bonding between the H3K18Ac side chain and Y993/N1039 in the TRIM33α BRD, we also analyzed the possibility of water-mediated interactions in the BRD pocket (details in the SI). This analysis indicates that there is lower water density predicted in the KAc binding pocket of TRIM33α compared to TRIM33β or TRIM24 (Figure S11). This lower density might result from N1039 not being present to interact with the water molecules. The lower water density could impact the water-mediated hydrogen bonding between Y993 and the H3K18Ac side chain that is observed in other BRDs.^57^ We then explored the hydrophobic contacts from the PLIP analysis to identify hydrophobic residues interacting with H3K18Ac, as TRIM33α formed a substantially greater number of hydrophobic contacts with H3K18Ac than the other BRDs (details in the SI). In this analysis, H3K18Ac was found to make more hydrophobic contacts with F1038 in TRIM33α than F979/F1038 in TRIM24/TRIM33β. This residue directly precedes N1039, and we hypothesize that in TRIM33α this residue can fluctuate to form hydrophobic interactions with H3K18Ac and stabilize the H3K18Ac–BRD interaction (see SI for details). Taken together, the MD simulations suggest that TRIM33α recognizes H3K18Ac mainly through hydrophobic contacts, while TRIM24 and TRIM33β form a more balanced split of hydrophobic and hydrophilic interactions with H3K18Ac.

### AlphaScreen Assay Established and Validated for Identifying TRIM24 and TRIM33 Ligands

**Figure 7:**
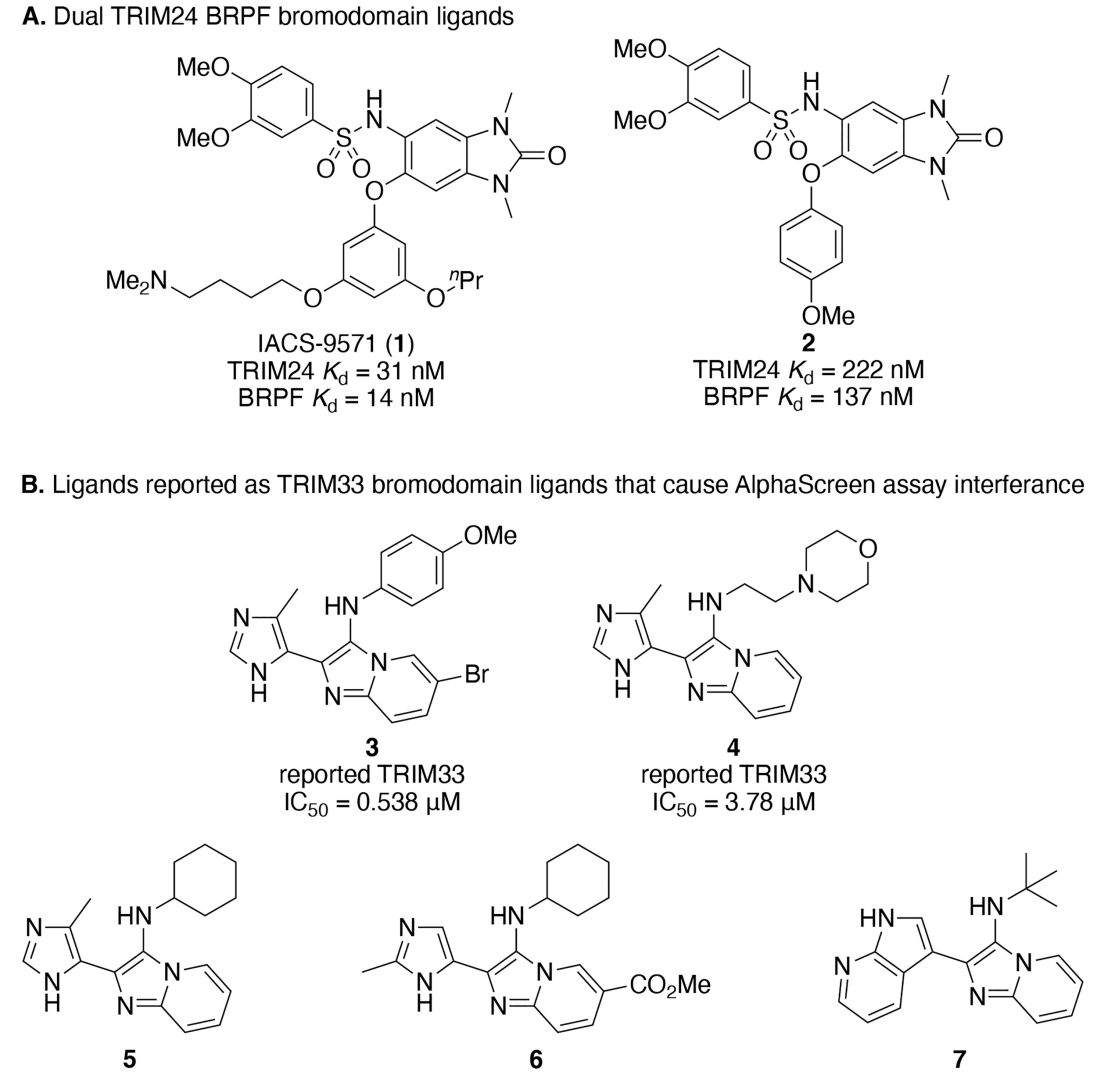
Previously reported ligands for the TRIM24 and TRIM33 BRDs. (A) TRIM24 ligand IACS-9571 (**1**) reported by Palmer et al.^46,47^ and compound **2** reported by Bennett et al.^48^ with their literature *K*_d_ (ITC) values shown. (B) Selection of compounds reported by Qi and Pei,^49^ which have also been synthesized in this study, with their literature IC_50_ (AlphaScreen) values shown. Our data show that all five compounds interfere with our TRIM24 and TRIM33 AlphaScreen assays. We could not detect binding of compounds **3** or **4** to the TRIM33β BRD using either ITC or waterLOGSY.

Only two high affinity probes have been reported for the TRIM24 BRD, and both compounds also bind to the BRPF1 BRD (Figure 7A).^46−48^ A patent from Qi and Pei claims a series of TRIM33 BRD ligands identified using an AlphaScreen (Figure 7B).^49^ The isoform of TRIM33 used in this work is not specified in the patent but was later confirmed to be TRIM33α (Qi, personal communication). We investigated using the previously reported TRIM24 and TRIM33 ligands to help validate our assay. Our AlphaScreen assay results for the TRIM24 ligand **2** agree with the literature reports (Figure S2), as displacement of H3_1–27_K18Ac was observed for TRIM24 but not for either isoform of TRIM33. An IC_50_ value of 219 nM was obtained for compound **2** binding to the TRIM24 BRD-PHD, which is very similar to the *K*_d_ value of 222 nM reported by Bennett et al.^48^ Three of the reported TRIM33 ligands (**3**, **4**, and **6**) showed apparent displacement of all three peptides from all three proteins in the AlphaScreen assay, with the additional two showing binding to TRIM24 and either weak or no binding to TRIM33 (Table S20, compounds **3**–**7**).

To determine whether compounds **3**–**7** are genuine ligands for TRIM33, we employed an AlphaScreen TruHits screen.^58^ The TruHits screen includes streptavidin-coated donor beads and biotinylated acceptor beads, which interact to generate a signal. Compounds that interfere with this control signal, such as fluorescence quenchers, insoluble light scatterers, ^1^O_2_ quenchers, and biotin mimetics, can be identified using this approach. In the TruHits screen,^59^ significant assay interference was observed from all five compounds (**3**–**7**, Table S21), meaning that AlphaScreen data generated for these compounds are unreliable. We therefore sought to investigate TRIM33 binding of compounds **3** and **4** using ITC and waterLOGSY approaches (Figures S3–6). These compounds were selected as they have a range of reported IC_50_ values for TRIM33. Neither compound showed binding to TRIM33β when assessed by ITC (Figures S3 and S4), using conditions based on the reported IC_50_ values. To determine whether these compounds are weak binders to TRIM33β, we subjected them to a waterLOGSY assay against TRIM33β. Again, neither compound showed any binding to this protein (Figures S5 and S6). Based on these data, we have concluded that these compounds, at least in our hands, are interfering with the TRIM24 and TRIM33 AlphaScreen assay and do not bind to TRIM33β. However, the use of different protein isoforms may account for some of the observed discrepancies.

### An Enriched High Throughput Screen Identified Ligands of the TRIM24 and TRIM33 Reader Domains

Having validated the AlphaScreen assay, we next sought to identify new ligands for TRIM33. For simplicity, we used the same peptides as the competing ligand in the AlphaScreen assay for TRIM24, TRIM33α, and TRIM33β. As these peptides have different affinity for each protein, IC_50_ values generated are not comparable between proteins.

**Figure 8:**
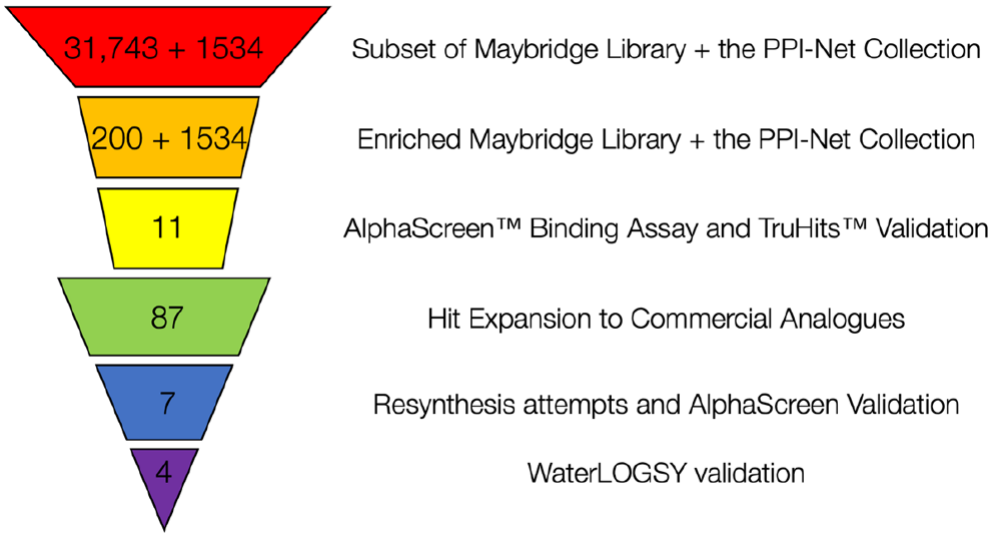
Screening and validation workflow used to identify ligands that bind the reader domains of TRIM24 and TRIM33α. The workflow resulted in ligands **8**–**11** (Figure 9).

**Figure 9:**
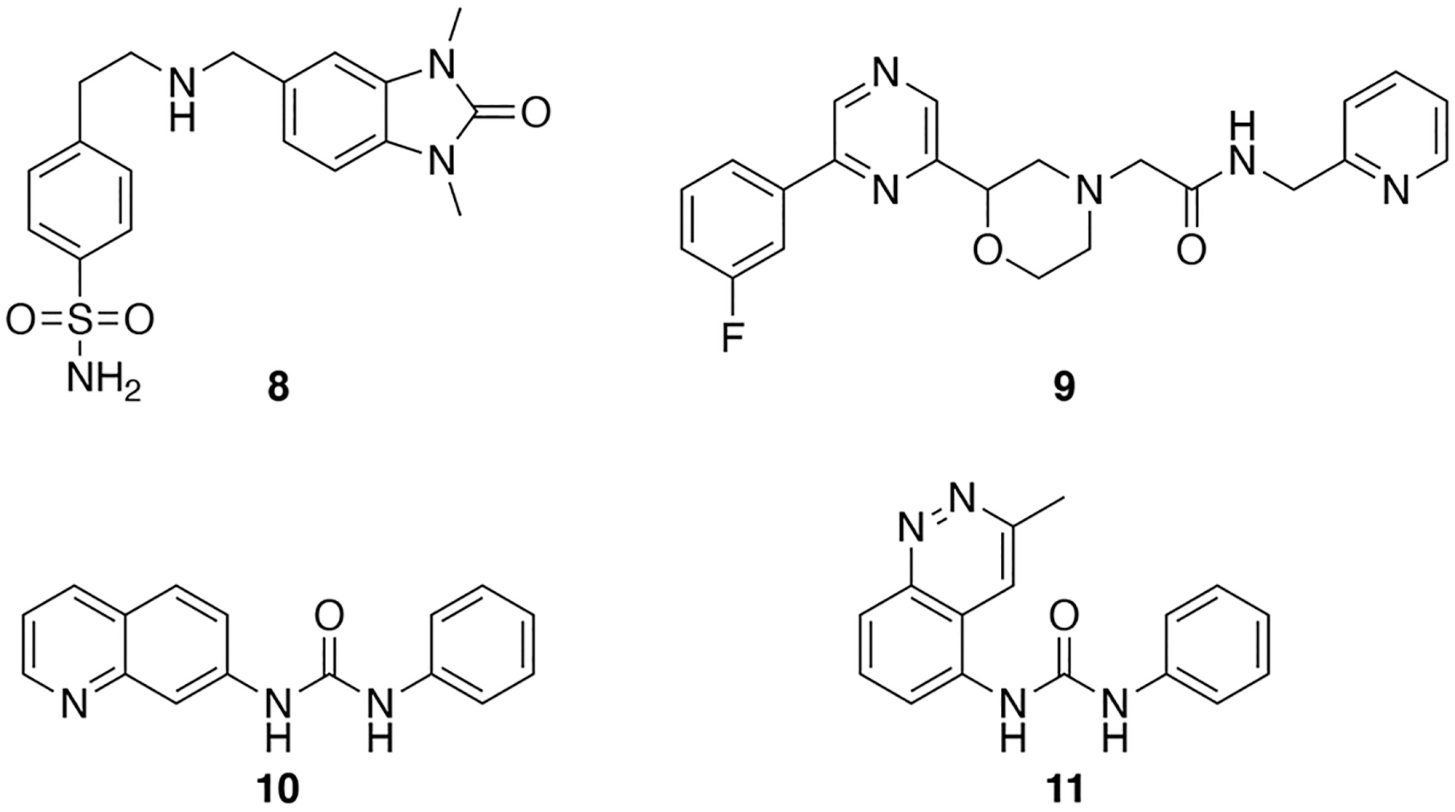
Compounds identified in the virtual screen and subsequently validated as ligands for TRIM24 and TRIM33.

To select compounds to be used in the AlphaScreen assay, 31743 compounds from the Maybridge fragment library (Fisher Scientific), along with 1534 compounds from the PPI-Net collection, were screened *in silico* against TRIM24 and TRIM33α. Based on the results from this screen, we identified an enriched fragment library of 200 compounds selected for predicted affinity, reliability of binding mode, and synthetic tractability. These compounds, along with the entire PPI-Net library, were screened against TRIM24, TRIM33α, and TRIM33β using a qualitative AlphaScreen response assay (data not shown). In this assay, the compounds were tested at two concentrations, 30 μM and 150 μM, as a rapid method of establishing activity. This study identified seven molecules from the PPI-Net library and four molecules from the Maybridge library as potential binders to one or more of the TRIM24 and TRIM33 reader domains. To increase the number of putative ligands, we identified additional ligands in the Maybridge Fragment library that show similarity to the hits, based on the Tanimoto coefficient (Figure 8).^59^ We only selected ligands that have a solubility forecast index (SFI)^60^ of <6.5. After validation of these molecules using NMR WaterLOGSY experiments and AlphaScreen TruHits screens (data not shown), four promising lead compounds, **8**–**11**, were identified (Figure 9).

Compound **8** contains the dimethylbenzimidazolone motif, which acts as the KAc mimic in the TRIM24 BRD ligands **1** and **2**, suggesting that this compound could bind to the TRIM33 BRD. Compounds **9**–**11**, however, possess no commonly employed KAc mimics, making it harder to predict their site of binding. To investigate where these compounds bind on the TRIM24 or 33 PHD-BRD cassette, we performed waterLOGSY experiments using the wild-type and mutant reader domains (Table 4). As expected, compound **8** bound to the WT BRDs of TRIM24, TRIM33α, and TRIM33β. It showed no binding to the TRIM24 N980F mutant and no clear binding to the TRIM33β N1039F mutant, as would be expected for a compound binding to the BRD. The binding of this compound was unaffected by the W889A or W828A mutants, indicating that it does not bind to the PHD of either TRIM24 or TRIM33. Compound **9** showed no binding to TRIM24, but it does bind to the WT TRIM33α and TRIM33β. Binding to TRIM33β, but not TRIM33α, was disrupted by both the W889A and N1039F mutations, suggesting that this compound interacts with both the BRD and PHD or binds between these domains. Compounds **10** and **11** bind to all three proteins, and their binding was unaffected by any of the mutants, indicating that these compounds do not bind to the BRD or PHD.

**Table 4:**
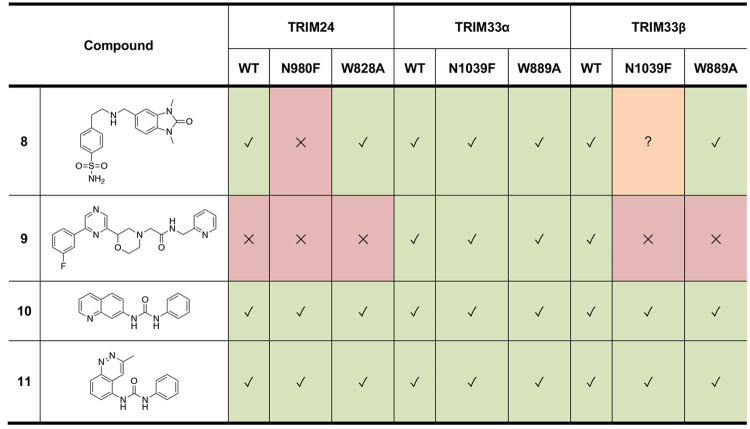
Qualitative WaterLOGSY Binding of WT and Mutant TRIM Proteins^a^

a √ is assigned to compounds that showed binding; ? is assigned when the result was ambiguous, and × is assigned when no-binding was observed.

Dose response AlphaScreen assays were performed to determine IC_50_ values of fragments **8** and **9** for each TRIM protein domain (Table 5). Three peptides were used in this assay, H3_1–27_K18Ac that detects binding to the BRD, H3_1–27_K9Me_3_ that detects binding to the PHD, and H3_1–27_K9Me_3_K18Ac that detects binding to both domains. As these peptides have different binding affinities to the BRDs and PHDs, IC_50_ values obtained using two different peptides cannot be compared. Using the H3_1–27_K18Ac peptide, compound **8** showed low affinity for TRIM24 BRD but IC_50_ values of 2.79 ± 0.83 μM and 18.4 ± 2.06 μM for TRIM33α and TRIM33β, respectively. This compound showed no affinity for the TRIM24 or TRIM33 PHDs when using the H3_1–27_K9Me_3_ peptide, consistent with the hypothesis that this compound binds to the BRD. Compound **8** also showed only very weak binding to BRD4(1), a representative member of the BET family of BCPs, at concentrations of >100 μM when assessed using an AlphaScreen assay (Figure S8).^50,51,62^

**Table 5:**
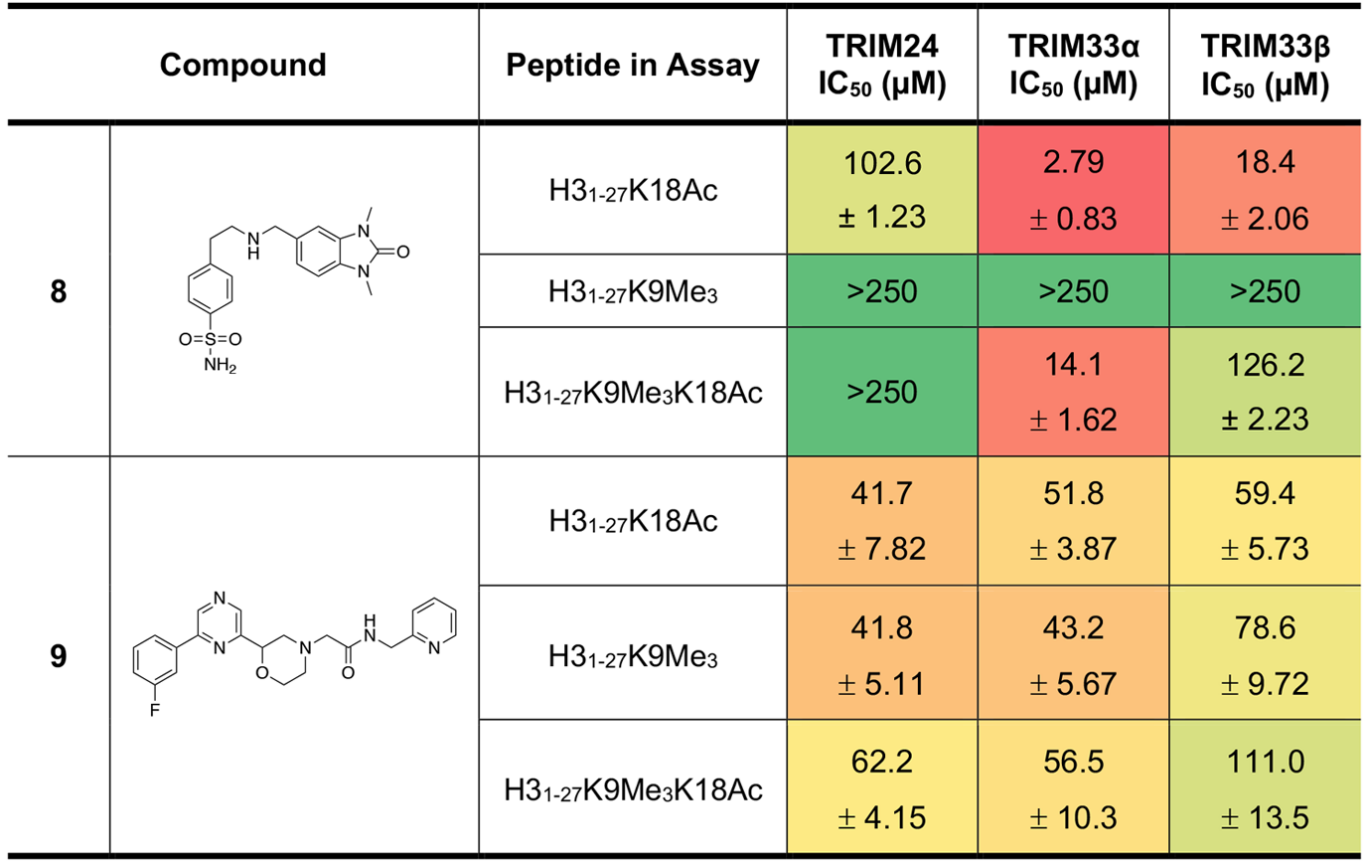
AlphaScreen Data for Compounds **8** and **9**^a^

a Note that the IC_50_ values cannot be compared between proteins. Errors are reported as the standard error of the mean between three measurements. A heatmap display is used with “hot” colors corresponding to lower IC_50_ values.

**Figure 10:**
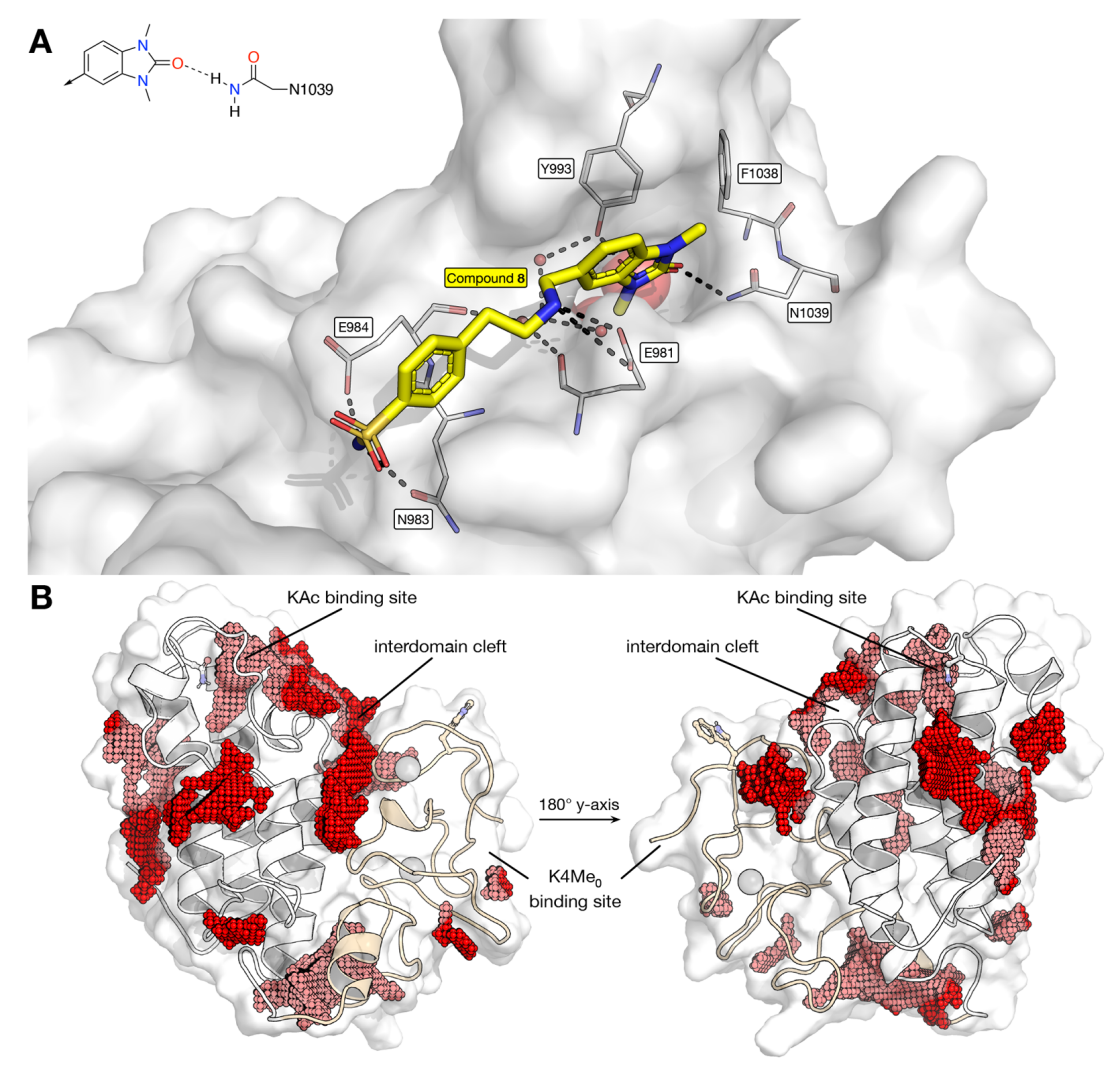
(A) Representation of compound **8** docked to the TRIM33β BRD using MOE. The docking studies suggest that the benzoimidazolone moiety of compound **8** acts as the KAc mimic and that the compound binds to the TRIM33β BRD by interacting with E981 and N1039. (B) Computational identification of cavities in the TRIM33β reader cassette. A knowledge-based cavity detection was performed using CCDC’s SuperStar package. This identified a deep cavity in the BRD, a long trench along the interdomain cleft, and a small region at the H3K4Me_0_ binding site, among others. There was no binding site detected in the vicinity of the H3K9Me_3_ binding region, consistent with its solvent-exposed nature. Figures generated using the PyMOL Molecular Graphics System, version 2.5, Schrödinger, LLC.

Given its similarity to previously reported TRIM24 BRD ligands,^46−48^ we proposed that compound **8** might occupy the KAc binding pocket of the TRIM33 BRD. Preliminary docking studies, using MOE, suggest that compound **8** (Figure 10A) can reside in the KAc binding pocket of TRIM33. The benzoimidazolone is predicted to act as the KAc mimic, with the oxygen atom proposed to form hydrogen bonds with N1039 and, via a structured water molecule, Y993. The benzylic amine is predicted to form a salt bridge with E981, which is the residue that binds to H3R17 contributing to the recognition of the H3 peptide (see above). This interaction is likely important for ligand affinity and explains the selectivity of this compound for TRIM33 over TRIM24, as the equivalent residue in TRIM24 is an alanine (A923). While reasonable, further computational studies are required to improve our understanding of the proposed binding mode of compound **8**.

Compound **9** shows weak affinity to both TRIM24 and TRIM33 when any of the peptides were used, consistent with the idea that this compound does not bind to exclusively the BRD or PHD of these proteins. To identify possible binding locations for compounds **9**–**11**, beyond the BRD and KAc, the CCDC SuperStar package was used to detect cavities in TRIM33β. This approach identified the KAc binding site, a shallow site at the K4Me_0_ binding region, and a larger cavity between the BRD and PHD (Figure 10B). The analysis also showed there was a cavity at the base of the construct. Based on these results, it is possible that compound **9** binds in this interdomain cleft, explaining the AlphaScreen assay results.

The low number of hits from our screen, with only 4 compounds confirmed from a total of 1821 screened (0.22% hit rate), coupled with the low affinity of these hits, indicates that TRIM33 is not an easily ligandable target based on the criteria of Vukovic and Huggins.^61^ This finding is consistent with the work of Vidler et al.^62^ who analyzed the druggability of a range of bromodomains, and classified the TRIM24 and TRIM33 difficult to drug. Given the challenges associated with identifying ligands for TRIM33, our data indicate that compound **8** is an important hit that could form the basis of selective, high affinity ligands for the TRIM33 BRDs.

## Conclusions

In conclusion, we report a comprehensive investigation into both peptide and small molecule ligands for the TRIM33 BRDs and PHDs. We have shown that, while the structure of the TRIM33α and TRIM33β BRDs differ in terms of N1039 location, they can both still bind to H3K18Ac with similar affinity. Interestingly, computational studies suggest that this affinity is derived mainly from hydrophobic interactions in the case of TRIM33α but from a mixture of hydrophilic and hydrophobic contacts for TRIM33β. This observation has implications for future ligand design. An AlphaScreen assay for the TRIM33 PHD-BRDs had low hit rate of 0.22%, indicating that TRIM33 PHDs and BRDs are difficult targets to ligand. However, we did identify compound **8**, which possesses a known KAc mimic; waterLOGSY experiments and initial docking studies predict that this compound binds to the TRIM33 BRDs. Furthermore, this compound shows little or no affinity for the BRDs of TRIM24 and BRD4(1). Given the difficulty of identifying ligands for TRIM33, compound **8** is an important hit that will enable development of selective high affinity ligands for the TRIM33 BRDs. The role of TRIM33 in the DNA damage response means that these compounds are of therapeutic interest for oncology indications. In addition, TRIM33 possesses E3 ligase activity, and so the identification of ligands for its BRDs raises the possibility that PROTACs that recruit TRIM33 to degrade a protein of interest can be developed. It remains to be seen whether ligands that are selective for TRIM33α or TRIM33β can be developed and whether they have different biological activity, either individually or as E3 ligase ligands in PROTACs. The data that we report here provide a strong foundation for such investigations into these important proteins.

## Supporting Information Available

Biological methods, chemical experimental section, computational methods sections, supplementary figures, and tables, NMR spectra and HPLC traces for compound **8** (PDF)
The preliminary PDB validation report for PDB entry 7ZDD (PDF)

## Accession Codes

The atomic coordinates and structure factors of human TRIM33 BRD-PHD isoform B in complex with the H3K9Ac peptide has been deposited in the Protein Data Bank (wwPDB) with accession code 5MR8. The atomic coordinates and structure factors of human TRIM33 BRD-PHD isoform B in complex with the H3K10Ac peptide has been deposited in the Protein Data Bank (wwPDB) with accession code 7ZDD.

## Abbreviations

TRIM: tripartite motif containing protein
BRD: bromodomain
PHD: plant homeodomain
KAc: acetylated lysine
KMe_*n*_: methylated lysine
