# Supplementary material for: Identification of Histone Peptide Binding Specificity and Small-Molecule Ligands for the TRIM33α and TRIM33β Bromodomains
Source: ACS Chem Biol. 2022 Sep 13;17(10):2753–68. doi: 10.1021/acschembio.2c00266 (PMC9594046; doi:10.1021/acschembio.2c00266)
Supplement: Supplementary file 1 — cb2c00266_si_001.pdf [file cb2c00266_si_001.pdf]

Identification of histone peptide binding specificity and small-molecule ligands for the TRIM33α and TRIM33β bromodomains

Angelina R. Sekirnik,[1,‡] Jessica K. Reynolds,[1,‡] Larissa See,[1,†] Joseph P. Bluck,[1,2] Amy R. Scorah,[1] Cynthia Tallant,[3] Bernadette Lee,[1] Katarzyna B. Leszczynska,[4,†] Rachel L. Grimley,[5] R. Ian Storer,[5] Marta Malattia,[6] Sara Crespillo,[6] Sofia Caria,[6] Stephanie Duclos,[6] Ester M. Hammond,[4] Stefan Knapp,[7,8] Garrett M. Morris,[9] Fernanda Duarte,[1] Philip C. Biggin,[2] Stuart J. Conway.[1,]*

Supporting Information

* To whom correspondence should be addressed: stuart.conway@chem.ox.ac.uk

[1]Department of Chemistry, Chemistry Research Laboratory, University of Oxford, Mansfield Road, Oxford OX1 3TA, U.K.

[2]Department of Biochemistry, University of Oxford, South Parks Road, Oxford OX1 3QU, U.K.

[3]Nuffield Department of Clinical Medicine, Structural Genomics Consortium, University of Oxford, Old Road Campus Research Building, Roosevelt Drive, Oxford, OX3 3TA, U.K.

[4]Oxford Institute for Radiation Oncology, Department of Oncology, University of Oxford, Old Road Campus Research Building, Oxford, OX3 7DQ, U.K.

[5]Worldwide Medicinal Chemistry, Discovery Biology, Pfizer Ltd, The Portway, Granta Park, Cambridge, CB21 6GS, U.K.

[6]Evotec (UK) Ltd, 90 Park Drive, Milton Park, Abingdon Oxfordshire, OX14 4RZ, U.K.

[7]Institute of Pharmaceutical Chemistry, Goethe University, Max-von-Laue-Strasse 9, D-60438 Frankfurt am Main, Germany.

[8]Structural Genomics Consortium, Goethe University, Buchmann Institute for Life Sciences (BMLS), Max-von-Laue-Strasse 15, D-60438 Frankfurt am Main, Germany.

[9]Department of Statistics, University of Oxford, 24-29 St Giles', Oxford, OX1 3LB, U.K.

Contents

1 Biological Methods 3
1.1 Materials and Reagents 3
1.2 General Methods 4
1.3 Microbiology 4
1.4 Protein Characterisation 9
1.5 Protein Expression and Purification 13
1.6 X-Ray Crystallography Methods 17
1.7 Assay Procedures 21
2 Chemistry Experimental Section 47
2.1 General Experimental Section 47
2.2 Experimental Details 49
3 Computational Methods 67
3.1 Virtual Screening Workflow 67
3.2 Propensity Map Generation 68
3.3 Molecular Dynamics Simulations 69
4 NMR spectra and HPLC trace for compound 8 85
5 References 88

1 Biological Methods

1.1 Materials and Reagents

Table S1. Suppliers of the materials and reagents used in this work.

Material	Supplier
Agarose	Bioline
SDS-PAGE reagents	Bio-Rad and Sigma
T4 DNA ligase and PfuTurbo® DNA polymerase	Stratagene
Q5® High-Fidelity DNA Polymerase	New England Biolabs (NEB)
IPTG and kanamycin	LaserBio Labs
dNTPs for PCR	Promega
HEPES Free Acid >99.5 %	Apollo Scientific
CHAPS	Calbiochem
AlphaScreen Histidine (NI chelate detection kit)	Perkin Elmer
384-well proxiplates plus	Perkin Elmer
Thermowell Sealing Tape	Corning

Materials and reagents were sourced as shown in Table S1. All other chemicals were obtained from Sigma-Aldrich unless otherwise stated. Purified (Milli-Q) water was obtained from a Millipore Elix® Reverse Osmosis system which was further purified by a Millipore Milli-Q® Synthesis system with a 0.22 µm filter on the outlet. Reagent quantities given as weight per volume (*w/v*) correspond to g/mL unless otherwise stated.

Plasmids

The pNIC28-Bsa4 TRIM24 (TIF1α) construct was kindly provided by the SGC, containing His_6-TRIM24D872 (PHD-Bromo). The pGEX-4T1 TRIM33α (TIF1γ-α) construct was kindly provided by Prof. Joan Massagué, containing Gst-TRIM33 (PHD-Bromo). The pNIC28-Bsa4 TRIM33β (TIF1γ-β) was kindly provided by Prof. Stefan Knapp.

1.2 General Methods

pH Measurements

The pH of solutions was determined using a Jenway pH Meter 3305, with an Aldrich glass/calomel combination electrode. Calibration was carried out between pH 4.0–7.0 or 7.0–10.0 immediately before use, with buffer solutions of phthalate (pH 4.0), phosphate (7.0) and borate (pH 10.0) from Fischer Scientific. Electrodes were stored in a 4 M aqueous potassium chloride solution.

Protein Concentration Measurements

Protein concentration was estimated by measuring the absorbance at 280 nm using a Nanodrop® ND-1000 spectrophotometer (Nanodrop® Technologies Inc.) with the 'Protein A280' program module according to the manufacturer's instructions.

Centrifugation

Samples of volume less than 1.5 mL were centrifuged at room temperature using an accuSpin™ benchtop centrifuge (Fischer Scientific) at 13 000 rpm. Protein samples of up to 2.0 mL were centrifuged in a Beckman Microfuge 22R Centrifuge (14 000 rpm) at 4 °C. Cell growth media and cell lysates were centrifuged in a Beckman Avanti J-25 (8500 rpm: JA-10 rotor; 15 000 rpm: JA-16.250 rotor and 24 000 rpm: JA-25.50 rotor, respectively). Proteins were concentrated using a Beckman Allegra X-30R centrifuge (4000 rpm, SX4400 swing bucket) at 4 °C. Plates were centrifuged using a Beckman Allegra X-30R centrifuge (2000 rpm, S6096 rotor) at 25 °C.

1.3 Microbiology

Standard sterile practices were followed thoroughly, using the Heracleus laminar flow hood when necessary. Media and equipment were sterilised by autoclaving at 121 °C for 20 min. Solutions of IPTG, antibiotics and other labile compounds were sterilised by filtering through 0.2 μm filters (Minisart®, Sartorius Stedim). Final concentrations of antibiotics unless indicated otherwise were: Ampicillin 100 μg/mL; Kanamycin 30 μg/mL.

Agarose Gel Electrophoresis

DNA samples were analysed and purified by gel electrophoresis of the DNA mixture on 1% agarose gels prepared with SYBR Safe™ DNA gel stain (Invitrogen). Samples were loaded after mixing with 6× loading buffer and run in TAE buffer on a Bio-Rad system at a constant potential of 80 V until desired fragment separation was achieved. Gels were visualised using a UV transilluminator (Gel logic 200) and the size of the fragments was referenced to one of the following molecular weight ladders: Quick-Load DNA marker, broad range (NEB); GeneRuler DNA Ladder, 1 kb (Thermo Scientific); GeneRuler DNA Ladder, 100 bp (Thermo Scientific).

Table S2. Reagents and quantities for buffers and gel required for agarose gel electrophoresis.

Buffer	Reagent	Amount required
50× TAE running buffer (per litre)	Tris-HCl	242.0 g
	Acetic acid (glacial)	57.0 mL
	EDTA (0.5 M)	100 mL
6× DNA loading buffer (per 10 mL)	ddH_2O	6.7 mL
	Bromophenol blue	25 mg
	Glycerol	3.3 mL
	Xylene cyanol FF	25 mg
DNA electrophoresis gel (per gel)	Agarose	0.5 g
	TAE buffer (1×)	50 mL
	SYBR® Safe DNA gel stain	5 µL

Digested DNA was purified using a QIAQuick Gel extraction Kit (QIAGEN) according to the manufacturer's instructions. DNA concentration was estimated by measuring the absorbance at 260 nm using a Nanodrop® ND-1000 spectrophotometer (Nanodrop® Technologies Inc.) with the 'DNA-50' program module according to the manufacturer's instructions.

DNA Sequencing

Plasmid sequencing was performed by Source BioScience Sequencing facility in the Department of Biochemistry (University of Oxford). T7 promoter and terminator sequence primers were used unless otherwise stated, on a 3730xl DNA Analyser-Titania.

Site-Directed Mutagenesis

Oligonucleotide primers were supplied by Sigma® Life Science as a dried pellet, resuspended in 10 mM Tris-HCl (pH 7.5) to a final primer concentration of 1 mM, and stored at −20 °C. These were designed with a minimal base-pair mismatch and maximal GC content, with a staggered overlap to prevent primer-dimer formation. Site-directed mutagenesis of plasmids was performed following the protocol of the QuikChange™ site-directed mutagenesis kit (Stratagene) with the quantities of reagents used shown in Table S3.

Table S3. Quantities and concentrations of reagents used for site-directed mutagenesis.

Reagent	Concentration	Amount used (µL)
DNA template	~100 ng/µL	0.5
5’ forward primer	10 pmol/µL	1.25
3’ reverse primer	10 pmol/µL	1.25
dNTP mix	10 mM of each	1.0
10 × PCR buffer	–.	5.0
Sterile Milli-Q water	–	40.0

A Techne Genius thermal cycler was programmed for an initial hold at 95 °C for 2 min to denature double stranded DNA, after which 1.0 µL of PfuTurbo® DNA polymerase was added. This was followed by 18 cycles of denaturing at 95 °C for 30 sec, annealing at 54 °C for 1 min, extension at 68 °C for 8 min. For inactivating mutations, 1.0 µL of PfuTurbo® DNA polymerase was added to the reaction mixture before incubation in a Labnet Multigene II Personal Thermal Cycler, with an initial denaturation step at 95 °C for 30 sec, followed by 16 cycles of denaturing at 95 °C for 30 sec, annealing

at 55 °C for 1 min, and extension at 68 °C for 7 min. The resulting samples were held at 4 °C before removal from the cycler, and digestion of parental DNA (centrifugation with 1 µL of restriction endonuclease *Dpn*I, followed by incubation at 37 °C for 1 h). Immediate transformation into competent *E. coli* XL10-Gold cells, yielded colonies containing plasmids with the desired sequence.

Cloning

Gene inserts were generated by PCR from parental DNA templates using primers. Amplification was carried out in a Labnet Multigene II Personal Thermal Cycler, with an initial denaturation step at 95 °C for 2 min, after which 1.0 µL of Q5® High-Fidelity DNA Polymerase (NEB) was added, followed by 25 cycles of denaturing at 95 °C for 30 sec, annealing at 55 °C for 1 min, and extension at 68 °C for 8 min. The resulting samples were held at 4 °C before removal from the cycler, and digestion of parental DNA (centrifugation with 1 µL of restriction endonuclease *Dpn*I, followed by incubation at 37 °C for 1 h).

Table S4. Quantities of reagents for cloning.

**Reagent**	**Concentration**	**Amount used (µL)**
DNA template	~100 ng/µL	0.5
5’ forward primer	10 pmol/µL	1.25
3’ reverse primer	10 pmol/µL	1.25
dNTP mix	10 mM of each	1.0
5 × Q5 buffer	–	4.0
Sterile Milli-Q water	–	11.0

Gibson Assembly

Cloning was performed using a one-step isothermal *in vitro* recombination reaction consisting of T5 exonuclease, Phusion DNA polymerase and Taq DNA ligase.[1] Linear vectors (~0.03 pmol) and gene inserts (~0.1 pmol) with complementary regions were incubated at 50 °C for 15-60 min in a 1:3 ratio with Gibson Assembly master mix (NEB): 20 µL reaction volume, 10 µL 2×GA master mix. 2 µL of

the reaction mixture was used directly to transform NEB 5-alpha (#C2987) competent cells, or diluted 1:3 to transform BL21 (DE3) Gold competent cells.

Competent Cells

Escherichia coli (*E. coli*) strains of the genotype BL21 (DE3) Gold: F^- ompT hsdS_B ($r_B^- m_B^-$) gal dcm (DE3) was used. DE3 denotes a chromosomal copy of the T7 RNA polymerase gene. Competent cells were thawed on ice for 20 min before 1–5 µL of plasmid DNA was added to 20 µL of the competent cells in pre-chilled 50 mL Falcon tubes. The tubes were left on ice for 30 min before placement into a water bath at 42 °C ('heat shocked') for 20 sec. The tubes were returned to ice for 5 min, 300 µL 2-TY media was added gently to each tube which was then incubated at 37 °C for 1 h before 100 µL of the transformation mixture was plated onto agar plates containing the appropriate antibiotic and incubated overnight.

Western Blotting

Cells were washed in PBS and lysed in SDS lysis buffer (10 mM Tris-Cl, pH 7.5, 0.1 mM EDTA, 0.1 mM EGTA, 0.5% SDS, 0.1 mM β-mercaptoethanol, protease/phosphatase inhibitors). After blocking in LiCOR blocking buffer, the following primary antibodies were used: TRIM33 (13387S, Cell Signaling), ALC-1 (ab197019, Abcam) and β-actin (AC-15, Santa Cruz). IRDye® 680 or IRDye® 800 secondary antibodies were used and the Odyssey infrared system (LI-COR) to visualize western blots.

1.4 Protein Characterisation

Table S5. Protein characterisation of TRIM24.

Gene	His_6-TRIM24 (BRD-PHD)
Vector backbone	pNIC28-Bsa4 (Kanamycin)
DNA sequence	ATGCACCATCATCATCATCATTCTTCTGGTGTAGATCTGGGT<br>ACCGAGAACCTGTACTTCCAATCCATGAATGAGGACTGGTG<br>TGCAGTTTGTCAAAACGGAGGGGAACTCCTCTGCTGTGAAA<br>AGTGCCCCAAAGTATTCCATCTTTCTTGTCATGTGCCCACAT<br>TGACAAATTTTCCAAGTGGAGAGTGGATTTGCACTTTCTGCC<br>GAGACTTATCTAAACCAGAAGTTGAATATGATTGTGATGCT<br>CCCAGTCACAACTCAGAAAAAAAGAAAACTGAAGGCCTTGT<br>TAAGTTAACACCTATAGATAAAAGGAAGTGTGAGCGCCTAC<br>TTTTATTTCTTTACTGCCATGAAATGAGCCTGGCTTTTCAAG<br>ACCCTGTTCCTCTAACTGTGCCTGATTATTACAAAATAATTA<br>AAAATCCAATGGATTTGTCAACCATCAAGAAAAGACTACAA<br>GAAGATTATTCCATGTACTCAAAACCTGAAGATTTTGTAGCT<br>GATTTTAGATTGATCTTTCAAAACTGTGCTGAATTCAATGAG<br>CCTGATTCAGAAGTAGCCAATGCTGGTATAAAACTTGAAAA<br>TTATTTTGAAGAACTTCTAAAGAACCTCTATCCAGAATGA
Amino acid sequence	MHHHHHHSSGVDLGTENLYFQSMNEDWCAVCQNGGELLCCE<br>KCPKVFHLSCHVPTLTNFPSGEWICTFCRDLSKPEVEYDCDAPS<br>HNSEKKKTEGLVKLTPIDKRKCERLLLFLYCHEMSLAFQDPVP<br>LTVPDYYKIIKNPMDLSTIKKRLQEDYSMYSKPEDFVADFRLIF<br>QNCAEFNEPDSEVANAGIKLENYFEELLKNLYPE
MW (Da)	23922
ε (M^{-1} cm^{-1})	25160

Table S6. Protein characterisation of TRIM33α.

Gene	His_6-TRIM33α **(BRD-PHD)**
Vector backbone	pNIC28-Bsa4 (Kanamycin)
DNA sequence	ATGCACCATCATCATCATCATTCTTCTGGTGTAGATCTGGGT ACCGAGAACCTGTACTTCCAATCCATGGACTGGTGTGCTGTC TGCCAAAACGGAGGAGATCTCTTGTGCTGCGAAAAATGTCC AAAGGTCTTTCATCTAACTTGTCATGTTCCAACACTACTTAG CTTTCCAAGTGGGGACTGGATATGCACATTTTGTAGAGATAT TGGAAAGCCAGAAGTTGAATATGATTGTGATAATTTGCAAC ATAGTAAGAAGGGGAAAACTGCGCAGGGGTTAAGCCCCGTG GACCAAAGGAAATGTGAACGTCTTCTGCTTTACCTCTATTGC CATGAATTAAGTATTGAATTCCAGGAGCCTGTTCCTGCTTCG ATACCAAACTACTATAAAATTATAAAGAAACCAATGGATTT ATCCACCGTGAAAAAGAAGCTTCAGAAAAAACATTCCCAAC ACTACCAAATCCCGGATGACTTTGTGGCCGATGTCCGTTTGA TCTTCAAGAACTGTGAAAGGTTTAATGAAATGATGAAAGTT GTTCAAGTTTATGCAGACACACAAGAGATTAATTTGAAGGC TGATTCAGAAGTAGCTCAGGCAGGGAAAGCAGTTGCATTGT ACTTTGAAGATAAACTCACAGAGATCTACTCAGACAGGACC TTCGCACCTTTGCCAGAGTTTGAGCAGGAAGAGGATGATGG TGAGGTAACTGAGGACTCTGATGAAGACTTTATACAGCCCC GCAGAAAACGCCTAAAGTCAGATGAGAGACCAGTACATATA AAGTAA
Amino Acid sequence	MHHHHHHSSGVDLGTENLYFQSMDWCAVCQNGGDLLCCEKC PKVFHLTCHVPTLLSFPSGDWICTFCRDIGKPEVEYDCDNLQHS KKGKTAQGLSPVDQRKCERLLLYLYCHELSIEFQEPVPASIPNY YKIIKKPMDLSTVKKKLQKKHSQHYQIPDDFVADVRLIFKNCE RFNEMMKVVQVYADTQEINLKADSEVAQAGKAVALYFEDKL TEIYSDRTFAPLPEFEQEEDDGEVTEDSDEDFIQPRRKRLKSDER PVHIK
MW (Da)	30502
ε (M^{-1} cm^{-1})	26650

Table S7. Protein characterisation of TRIM33β (His_6).

Gene	His_6-TRIM33β (BRD-PHD)
Plasmid vector	pNIC28-Bsa4 (Kanamycin)
DNA sequence	ATGCACCATCATCATCATCATTCTTCTGGTGTAGATCTGGGT ACCGAGAACTGTACTTCCAATCCATGGACTGGTGTGCTGTCT GCCAAAACGGAGGAGATCTCTTGTGCTGCGAAAAATGTCCA AAGGTCTTTCATCTAACTTGTCATGTTCCAACACTACTTAGC TTTCCAAGTGGGGACTGGATATGCACATTTTGTAGAGATATT GGAAAGCCAGAAGTTGAATATGATTGTGATAATTTGCAACA TAGTAAGAAGGGGAAAACTGCGCAGGGGTTAAGCCCCGTGG ACCAAAGGAAATGTGAACGTCTTCTGCTTTACCTCTATTGCC ATGAATTAAGTATTGAATTCCAGGAGCCTGTTCCTGCTTCGA TACCAAACTACTATAAAATTATAAAGAAACCAATGGATTTA TCCACCGTGAAAAAGAAGCTTCAGAAAAAACATTCCCAACA CTACCAAATCCCGGATGACTTTGTGGCCGATGTCCGTTTGAT CTTCAAGAACTGTGAAAGGTTTAATGAGGCTGATTCAGAAG TAGCTCAGGCAGGGAAAGCAGTTGCATTGTACTTTGAAGAT AAACTCACAGAGATCTACTCAGACAGGACCTTCGCACCTTTG CCAGAGTTTGAGCAGGAAGAGGATGATGGTGAGGTAACTGA GGACTCTGATGAAGACTTTATACAGCCCCGCAGAAAACGCC TAAAGTCAGATGAGAGACCAGTACATATAAAGTAA
Amino Acid sequence	MHHHHHHSSGVDLGTENLYFQSMDWCAVCQNGGDLLCCEKC PKVFHLTCHVPTLLSFPSGDWICTFCRDIGKPEVEYDCDNLQHS KKGKTAQGLSPVDQRKCERLLLYLYCHELSIEFQEPVPASIPNY YKIIKKPMDLSTVKKKLQKKHSQHYQIPDDFVADVRLIFKNCE RFNEADSEVAQAGKAVALYFEDKLTEIYSDRTFAPLPEFEQEED DGEVTEDSDEDFIQPRRKRLKSDERPVHIK
MW (Da)	28509
ε (M^{-1} cm^{-1})	25160

Table S8. Protein characterisation of TRIM33β (GST).

Gene	His_6-SUMO-TRIM33 (BRD-PHD)
Plasmid vector	pETM11-SUMO3 (Kanamycin)
DNA sequence	ATGAAACATCACCATCACCATCACCCCATGAGCGATTACGA CATCCCCACTACTGAGAATCTTTATTTTCAGGGCGCCATGGG CAACGATCACATTAACCTGAAAGTGGCCGGTCAAGACGGTA GCGTAGTCCAGTTTAAAATCAAACGCCACACCCCTCTGTCG AAACTGATGAAAGCCTATTGTGAACGCCAAGGTCTGTCTAT GCGTCAGATCCGTTTTCGCTTCGATGGACAGCCGATTAACG AAACCGACACTCCAGCACAGCTGGAAATGGAAGATGAGGA CACCATTGACGTGTTCCAGCAACAGACCGGTGGATCCATGG ACTGGTGTGCTGTCTGCCAAAACGGAGGAGATCTCTTGTGC TGCGAAAAATGTCCAAAGGTCTTTCATCTAACTTGTCATGTT CCAACACTACTTAGCTTTCCAAGTGGGGACTGGATATGCAC ATTTTGTAGAGATATTGGAAAGCCAGAAGTTGAATATGATT GTGATAATTTGCAACATAGTAAGAAGGGGAAAACTGCGCA GGGGTTAAGCCCCGTGGACCAAAGGAAATGTGAACGTCTTC TGCTTTACCTCTATTGCCATGAATTAAGTATTGAATTCCAGG AGCCTGTTCCTGCTTCGATACCAAACTACTATAAAATTATAA AGAAACCAATGGATTTATCCACCGTGAAAAAGAAGCTTCAG AAAAAACATTCCCAACACTACCAAATCCCGGATGACTTTGT GGCCGATGTCCGTTTGATCTTCAAGAACTGTGAAAGGTTTA ATGAAATGATGAAAGTTGTTCAAGTTTATGCAGACACACAA GAGATTAATTTGAAGGCTGATTCAGAAGTAGCTCAGGCAGG GAAAGCAGTTGCATTGTACTTTGAAGATAAACTCACAGAGA TCTACTCAGACAGGACCTTCGCACCTTTGCCAGAGTTTGAGC AGGAAGAGGATGATGGTGAGGTAACTGAGGACTCTGATGA AGACTTTATACAGCCCCGCAGAAAACGCCTAAAGTCAGATG AGAGACCAGTACATATAAAGTAAAAGCTTGCGGCCGCACTC GAGCACCACCACCACCACCAC
Amino acid sequence	MKHHHHHHPMSDYDIPTTENLYFQGAMGNDHINLKVAGQDG SVVQFKIKRHTPLSKLMKAYCERQGLSMRQIRFRFDGQPINET DTPAQLEMEDEDTIDVFQQQTGGSMDWCAVCQNGGDLLCCE KCPKVFHLTCHVPTLLSFPSGDWICTFCRDIGKPEVEYDCDNLQ HSKKGKTAQGLSPVDQRKCERLLLYLYCHELSIEFQEPVPASIP NYYKIIKKPMDLSTVKKKLQKKHSQHYQIPDDFVADVRLIFKN CERFNEMMKVVQVYADTQEINLKADSEVAQAGKAVALYFED KLTEIYSDRTFAPLPEFEQEEDDGEVTEDSDEDFIQPRRKRLKS DERPVHIK-
MW (Da)	40384
ε (M^{-1} cm^{-1})	29630

1.5 Protein Expression and Purification

All constructs were transformed into *E. coli* BL21 (DE3) Gold cells for expression.

Bacterial Growths

Table S9. Quantities of reagents required for growth media.

Media	Reagent	Amount required per litre (g)
Terrific Broth	Tryptone (pancreatic digest of casein)	12.0
	Yeast extract	24.0
	K_2HPO_4	9.4
	KH_2PO_4	2.2
2× Typtone-Yeast (2-TY) Extract	Bacto tryptone	16.0
	Yeast extract	10.0
	NaCl	5.0

All growth media were autoclaved (*Thermo Live Sciences* MAT 490 LEI) at 121 °C for 45 min before use. Optical density readings were taken in 1.6 mL cuvettes with 5 × dilution in Milli-Q water against a reference sample of the growth media at zero time. The absorbance was measured at 600 nm using a Novaspec® II spectrophotometer.

Bacterial plate cultures were prepared using solid 2-TY medium with the appropriate antibiotic, and streaked in a laminar flow hood (HereSafe® Model KS12 Class II Biosafety Cabinet) using sterile equipment. Following inoculation, these were inverted and grown overnight at 37 °C in a Heraeus® TypB 6030 incubator. Bacterial plates with resulting bacterial colonies were stored in the dark at 4 °C. Small-scale growths for starter cultures (100 mL of 2-TY media containing the appropriate antibiotic in 500 mL Duran flasks), were inoculated with a single colony from an agar plate, and grown at 37 °C overnight.

Expression Trials

Starter cultures were used to inoculate 100 mL (in 500 mL Duran flasks) of 2-TY media containing the appropriate antibiotic. Cultures were incubated at 37 °C until they reached an OD_{600} of 1.0. Cultures

were transferred to incubators held at 18 °C or 28 °C and allowed to equilibrate for 15 min before induction with $ZnCl_2$ to a final concentration of 0.1 mM and IPTG to final concentrations of 0.1 mM, 0.25 mM or 0.5 mM. Cultures were incubated at these temperatures overnight, before being pelleted by centrifugation, resuspended in 300 µL HisTrap column binding buffer for whole-cell analysis by SDS-PAGE.

Large-Scale Growth

Large-scale recombinant protein expression was performed using 2000 mL PYREX® narrow-mouth graduated Erlenmeyer flasks containing 600 mL of 2-TY medium containing the appropriate antibiotic. Flasks were inoculated with 1% of the bacterial starter culture and grown at 37 °C until they reached an OD_{600} of 1.0. Cultures were equilibrated to 18 °C and induced with $ZnCl_2$ (0.1 mM) and IPTG (0.25 mM), before incubation overnight. After this time, cultures were centrifuged and the resulting bacterial pellet frozen in a sealable plastic bag at −80 °C.

Protein Purification

Cell pellets were dissolved in binding buffer (5× mass of cell pellet *w/v*) and lysed by sonication, applying thirty 10 sec bursts interrupted by 10 sec pauses. The lysate was clarified by centrifugation and the supernatant decanted, filtered through a 0.45 µm filter, and applied to a purification column.

Table S10. Quantities of reagents needed for buffers for protein purification.

Reagents	IMAC buffers (pH 7.6)			
	**Binding**	**Wash**	**Elution**	**Strip**
HEPES	50 mM	50 mM	50 mM	50 mM
NaCl	0.5 M	0.5 M	0.5 M	0.5 M
Imidazole	5 mM	30 mM	500 mM	–
EDTA	–	–	–	100 mM

Immobilised metal affinity chromatography (IMAC) was performed with a HisTrap^{TM} (6 mL) column (GE Healthcare) charged with 20 mL of 100 mM $NiSO_4$ and washed with 50 mL of binding buffer prior to loading the cell lysate at 1 mL/min. The column was then washed with approximately 100 mL binding

buffer. When all residual products of bacterial fermentation were eluted from the column by binding buffer, the protein of interest was batch-eluted with 30 mL elution buffer. Eluted protein was collected and fractionated; fractions containing the highest levels of pure protein (as determined by SDS-PAGE gels) were concentrated using Amicon Ultracell-30k concentrators (Millipore) in a Beckmann Allegra™ 21R centrifuge.

Further purification was achieved by gel filtration chromatography. Concentrated fractions (<2 mL) were loaded on a 150/300 mL Superdex 75 size exclusion chromatography column (Amersham). Eluted protein was collected and fractionated; fractions containing the highest levels of pure protein (as determined by SDS-PAGE gels) were concentrated (to 50–500 μM) using Amicon Ultracell-30k concentrators (Millipore) in a Beckmann Allegra™ 21R centrifuge.

Protein concentration was determined by measuring the absorbance at 280 nm using a Nanodrop® ND-1000 spectrophotometer (Nanodrop® Technologies Inc.) as stated above. Protein molecular weight and molar extinction coefficient were calculated using ProtParam on the ExPASy Bioinformatics Resource Portal.

Whole-Protein Mass Spectrometry

Intact masses of proteins, purified as described above, were recorded by liquid chromatography/mass spectrometry (LC/MS) using a ZMD (Waters®) single quadrupole mass spectrometer, interfaced with a Hewlett Packard® Series 1050 liquid chromatography and sample handling system. The protein sample (7 μL) was injected onto a Grace Vydac 214TP C4 (250 mm × 4.6 mm) reverse-phase HPLC column and eluted at 1 mL/min using a gradient system from Solvent A (0.1 % formic acid in H_2O) to Solvent B (0.1% formic acid in acetonitrile). Samples were injected directly into the mass spectrometer. The following MS parameters were used: polarity: ES^+; capillary voltage: 3,000 V; sample cone voltage: 35 V; extraction cone voltage: 1 V; desolvation temperature: 250 °C; source temperature: 100 °C; cone gas flow: 100 L/h; desolvation gas flow (N_2): 830 L/h. Sodium formate was used to calibrate the instrument. Spectra were processed using MassLynx™ v4.0 (Waters Corporation) with the Maximum

Entropy method (MaxEnt1) employed for deconvolution of intact mass (1 Da resolution). Expected masses were calculated using ExPASy ProtParam tool on the Bioinformatics Resource Portal.

SDS-PAGE

The purity of proteins was analysed by PAGE. Gels were run on a mini-PROTEAN Tetra Electrophoresis System (Bio-Rad) at a constant potential of 200 V. Following electrophoresis, gels were stained for 20 min with InstantBlue™ Coomasie® stain (Expedeon). For denaturing PAGE analyses, samples were prepared by mixing with sample loading buffer (2×) and incubation at 100 °C for 3 min. All gels were loaded with one of the following molecular weight ladders: SeeBlue® prestained standard (Thermo Fisher Scientific); BLUEstain™ protein ladder, 11-245 kDa (Gold Biotechnology); PageRuler Plus prestained protein Ladder (Thermo Fisher Scientific); blue protein standard, broad range (NEB); colour protein standard, broad range (NEB).

Table S11. Gel compositions (quantities for 1 gel).

Reagent	Resolving gel		Stacking gel
	12.5 %	15 %	4 %
Tris-HCl (1.5 M, pH 8.8)	1.25 mL	1.25 mL	–
Tris-HCl (0.5 M, pH 6.8)	–	–	0.63 mL
Milli-Q water	1.57 mL	1.15 mL	1.57 mL
30 % (*w/v*) Acrylamide	2.08 mL	2.50 mL	0.25 mL
SDS (10 % *w/v*)	50 µL	50 µL	25 µL
10 % (*w/v*) APS	50 µL	50 µL	25 µL
TEMED	4 µL	4 µL	2.5 µL

Gels were prepared using 70 mm × 100 mm glass plates, with 0.75 mm spacers. TEMED and freshly prepared ammonium persulfate were added just prior to pouring the gels. The resolving gel was cast with the addition of a separate layer of methanol to ensure a level surface. Once the resolving layer had polymerized, methanol was removed and the stacking gel was cast.

Table S12. Compositions of buffers and stain for SDS-PAGE.

Buffer	Reagent	Composition	Amount required per litre
SDS-PAGE running buffer	Tris-HCl		30 g
	Glycine		144 g
	SDS		10 g
SDS-PAGE sample loading buffer (pH 6.8)	Tris-HCl	0.5 M	10 mL
	Bromophenol blue	0.2 % *w/v*	0.2 g
	SDS	10 % *w/v*	20 mL
	Glycerol		12 mL
	β-mercaptoethanol		5.0 mL
SDS-PAGE stain	Coomassie® brilliant blue R	0.25 %	2.5 mL
	acetic acid	10 %	100 mL
	methanol	30 %	300 mL

SDS-PAGE destain was of the same composition without the presence of Coomassie® brilliant blue R.

Circular Dichroism

For investigation of protein secondary structure using circular dichroism (CD), measurements were acquired using a Chirascan CD spectrometer (Applied Photophysics) with a Peltier temperature-controlled cell holder. All experiments were performed in a 0.1 cm path length cuvette using 0.1–0.2 mg/mL protein in 10 mM sodium phosphate buffer (pH 8.0). Data were recorded from 240 to 185 nm, at 0.5 nm intervals, and each data point was averaged for 3 sec. Spectra were base-line corrected and smoothed using the Savitzky-Golay filter.

1.6 X-Ray Crystallography Methods

1.6.1 Methods for 5MR8

DNA Construct and Protein Purification

The cDNA encoding human TRIM33 (UniProt: Q9UPN9-2) was synthesized by GenScript with codon optimized for *E. coli.* expression. cDNA sequence was used as a template to amplify the tandem module PHD-Bromodomain (residues 883-1073) region. This was further subcloned into a pET15 derived expression vector (pSUMO), using ligation independent cloning. This vector expressed the recombinant

protein with a 6×His tag fused with small ubiquitin like modifier (SUMO)-1 fusion tag at the N-terminus.

Expression construct was transformed into *E. coli* competent BL21 (DE3)-R3-pRARE2 cells (phage-resistant derivative with a pRARE plasmid encoding rare codon tRNAs). Cells were grown at 37 ºC in Terrific Broth (Sigma) from overnight cultures until A600 reached between 0.8-1.1, then the media was cooled and 0.2 mM isopropyl-β-D-thiogalactopyranoside (IPTG) plus 0.1 mM $ZnCl_2$ was added to induce the protein expression at 18 ºC for 16 hours. *E. coli* overexpressed cells were lysed using an EmulsiFlex-C5 high-pressure homogenizer in lysis buffer from the nickel affinity column (HisTrap Chelating FF 5mL) in the presence of Protease Inhibitor Cocktail EDTA-free (Roche). Lysates were cleared by centrifugation and the supernatant was loaded onto a nickel column and eluted in an imidazole linear gradient. The eluted protein was collected and treated overnight with SENP1 (SUMO endoprotease-1) at 4 ºC to remove the N-terminal tag. Digested protein was loaded onto a nickel column again to remove the cleaved 6×His-SUMO tag and the hexa-histidine expression tag protease used. The flow through containing the untagged TRIM33 isoform B protein was collected and further dialyzed and purified by an ion exchange chromatography (RESOURCE Q 6 ml GE Healthcare Life Sciences). Finally, a polishing step was performed through a size exclusion chromatography (HiLoad 16/600 Superdex 75). Electro-spray Mass Spectrometry (ESI-TOF) analysis of purified sample showed single peak and confirmed mass of 22245.12 Da.

Crystallization

Histone peptide H3K9Ac complex crystallization with tandem TRIM33 isoform B: protein was concentrated up to 18.7 mg/mL and co-crystallized with 1:10 M excess of the 9-mer peptide in 100 nL + 200 nL sitting drop at 293.15 K. Prism-like crystals appeared in four days in optimized crystallization conditions of 0.1 M Tris pH 8.5, 25% PEG 3350. The protein buffer was 20 mM Hepes pH 7.5, 200 mM NaCl, 2 mM DTT. The crystals were flash-frozen at 100 K in a nitrogen gas stream in the cryoprotectant with 15 % ethylene glycol.

Data Collection and Structure Determination

A dataset was collected for the co-crystal of TRIM33 PHD-Bromodomain isoform B in complex with histone H3K9ac peptide at Diamond Light Source (beamline I03) and processed to 1.74 Å using XDS and Aimless/SCALA. Crystal structure was elucidated by molecular replacement using the apo TRIM33 isoform A (3UM5.pdb) structure as template with PHASER. Omit map showed clear electron density to fit all residues from the ARTKQTARKAc peptide sequence. A total of 50 cycles of automated protein chain tracing starting from experimental phases was computed using ARP/wARP. Further manual building and refinement were carried out using Coot and REFMAC5 plus final refinement cycles with PHENIX. Model validation was carried out using MolProbity. All data collections and refinement statistics are shown in Table 29.

1.6.2 Methods for 7ZDD

DNA Construct and Protein Purification

Protein expression construct encoding the human TRIM33ß (882-1073) (Uniprot accession number: Q9UPN9-2) was obtained as a synthetic cDNA codon (Genscript, Nanjing, China) optimized for *E. coli* expression and cloned into the pET-28a vector (GE Healthcare, Chicago, IL, USA). Protein overexpression was performed using *E. coli* BL21 (DE3) competent cells (New England Biolabs, MA, USA) in 2xYT media (Teknova, CA, USA) supplemented with 50 $\mu g.mL^{-1}$ kanamycin at 37 °C until the optical density at 600 nm (OD600) reached 0.3. The temperature was then decreased to 16 °C and when OD600 reached 1.0, the expression was induced by adding 0.1 mM IPTG and 0.2 mM $ZnCl_2$. Expression induction was conducted at 16 °C with 200 rpm shaking overnight.

Bacterial cells were harvested by centrifugation at 4000 *g* for 20 min using an Avanti – HC centrifuge (Beckman Coulter, Pasadena, CA, USA), resuspended in lysis buffer (50 mM HEPES pH 7.6, 500 mM NaCl, 10 mM β-mercaptoethanol, 10% *v/v* glycerol, 0.1 mM $ZnCl_2$, 10 $U.mL^{-1}$ benzonase, protease inhibitor tablets without EDTA (Roche, Basel, Switzerland)) and lysed by cell disruption with 2 cycles at 20 kPsi using a continuous flow (CF1) cell disruptor (Constant Systems Ltd., Daventry, UK). Lysates

were clarified by centrifugation at 235 000 *g* for 45 min using an Optima XPN-80 ultracentrifuge (Beckman Coulter, Pasadena, CA, USA).

The His-tagged TRIM33ß protein was purified with a HisTrap crude FF column (GE Healthcare), previously equilibrated in 50 mM HEPES pH 7.6 buffer, 500 mM NaCl, 1 mM DTT, 10 % *v/v* glycerol, 25 mM imidazole). TRIM33β protein was eluted using an imidazole gradient (25 – 500 mM) in the same equilibration buffer. The pooled fractions were dialyzed into affinity chromatography equilibration buffer overnight in the presence of TEV protease (ratio enzyme: protein 1:40) to remove the His tag. Cleaved protein was retrieved using subtractive immobilized metal affinity chromatography with a HisTrap crude FF column (GE Healthcare, IL, US) by recovering the flow-through. TRIM33β protein was then further purified by size exclusion chromatography on a Superdex S200 16/600 column (GE Healthcare, IL, US) equilibrated in 19 mM HEPES pH 7.5, 145 mM sodium chloride, 1 mM TCEP, 0.1 mM zinc chloride and 25mM sodium citrate and eluted as a single peak.

Crystallization

The protein was concentrated to 22 mg/mL and incubated with the H3K10Ac peptide at 10.5 mM final concentration for 1 h on ice. Both the protein and the peptide stocks were stored in 19 mM HEPES pH 7.5, 145 mM sodium chloride, 1 mM TCEP, 0.1 mM zinc chloride and 25 mM sodium citrate. Crystallization was carried out with by mixing an equal volume of protein and reservoir solution in sitting drop at 295.15 K. Crystals appeared after one day in a crystallization condition made of 0.2 M ammonium chloride, 0.1 M sodium acetate pH 5.0 and 20% *w/v* PEG 6000. Crystals were flash-frozen at 100 K in liquid nitrogen after addition of 15% *v/v* ethylene glycol as cryoprotectant.

Data Collection and Structure Determination

A dataset was collected for the co-crystal of TRIM33 PHD-Bromodomain isoform B in complex with histone H3K10Ac peptide at Diamond Light Source (i03 beamline) and processed to 1.62 Å (anisotropic dataset: 1.56, 2.36 and 1.71 Å) using autoPROC/Staraniso (XDS and Aimless). The crystal structure was elucidated by molecular replacement using TRIM33 PHD-Bromodomain isoform B (PDB code 5MR8, only the TRIM33 chain was used) structure as template in Phaser. Clear density enabled the

modelling of the H3K9ac histone peptide. Manual model building and refinement were carried out using Coot and BUSTER. Model validation carried out using MolProbilty. All data collection and refinement statistics are shown in Table 29.

1.7 Assay Procedures

1.7.1 AlphaScreen™

General Procedure

Assay buffer was freshly prepared each day by supplementing HEPES buffer with BSA, the pH was adjusted to 7.6 and filter sterilized through a 0.22 µm filter, and stored at 4 °C until use. For incubation steps, the plate was sealed, shaken for 10 sec at 600 rpm on a plate oscillator, and incubated at room temperature in the dark. Compounds (5 µL) were dispensed into a ProxiPlate-384 Plus (Perkin Elmer) in triplicate, as point concentrations or serial buffer dilutions from 50 mM DMSO solutions. Protein-peptide mix (7 µL) was dispensed into each well using a Thermo-Fisher electronic multi-channel pipette, and incubated for 30 min. AlphaScreen bead mix (8 µL; 25 µg/mL of acceptor and donor beads) was added to each well, taking adequate precautions to avoid light exposure, before being incubated for 1 h. The plate was read using a Synergy™ 2 MultiMode Microplate Reader using the built-in AlphaScreen 384 ProxiPlate function: excitation 680 nm, 0.18 sec; emission 570 nm, 0.37 sec.

Table S13. Composition of AlphaScreen™ buffer.

Buffer	Reagent	Composition	Amount required per litre
AlphaScreen™ buffer (pH 7.6)	HEPES	25 mM	5.95 g
	NaCl	100 mM	5.84 g
	CHAPS	0.05 % *w/v*	0.5 g
	BSA	0.1 *w/v*	1 g

Single shot screening was performed in triplicate. Compounds were tested at 30 µM and 150 µM with <0.5% DMSO. Inhibition is reported as a reduction in signal arising from peptide-bromodomain interaction, normalised to a DMSO control. Signal response curves were obtained for peptides or compounds in triplicate, with serial 1:2 dilutions. Samples containing buffer or DMSO in place of

compound were included in all assays for normalisation. Data were processed by fitting a four-parameter equation shown below to calculate IC_{50} values using GraFit version 7.0.3[2] or GraphPad Prism 7 software.

$$Y = Minimum + \frac{Maximum - minimum}{1 + 10^{(\log IC_{50} - X)(Hill\ slope)}}, where\ X = \log\ (compound\ concentration)$$

Signal Response Assay

Assay buffer was prepared as above. For incubation steps, the plate was sealed, shaken for 10 sec at 600 rpm on a plate oscillator, and incubated at room temperature in the dark. Peptides (5 μL) were dispensed into a ProxiPlate-384 Plus (Perkin Elmer) in triplicate, as point concentrations or serial buffer dilutions from 100 μM HEPES buffer stock solutions. Protein (7 μL) was dispensed into each well using a Thermo-Fisher electronic multi-channel pipette, and incubated for 30 min. AlphaScreen bead mix (8 μL; 25 μg/mL of acceptor and donor beads) was added to each well, avoiding light exposure, before being incubated for 1 h. The plate was read using a Synergy™ 2 MultiMode Microplate Reader using the built-in AlphaScreen 384 ProxiPlate function: excitation 680 nm, 0.18 sec; emission 570 nm, 0.37 sec.

Assay Composition

Ni^{2+} chelate acceptor and streptavidin donor beads were prepared as a mixture in a 1:300 or 1:400 dilution as required (0.005–0.007 mg/mL FAC); biotinylated peptide employed, and final assay concentrations were as detailed in table below. Assay well volume was 20 μL.

Table S14. Final assay concentrations of protein, biotinylated peptides, and beads for AlphaScreen™. The biotin is conjugated to the ε-nitrogen of K27, except for $H3_{5\text{-}24}$K14Ac where it is conjugated to the ε-nitrogen of K23.

**Protein (His_6-)**	**FAC protein (nM)**	**Peptide**	**Sequence**	**FAC peptide (nM)**	**FAC beads (µg/mL)**
TRIM24	100	**B1**	$H3_{1\text{-}27}K9Me_3$	4	5
	25	**B2**	$H3_{1\text{-}27}$K18Ac	8	5
	25	**B3**	$H3_{1\text{-}27}$K23Ac	4	5
	25	**B4**	$H3_{1\text{-}27}K9Me_3$K18Ac	4	5
	25	**B5**	$H3_{1\text{-}27}K9Me_3$K23Ac	4	5
	25	**B6**	$H3_{5\text{-}24}$K14Ac	15	5
	25	**B7**	$H3_{1\text{-}27}$K14Ac	15	5
	25	**UB7**	$H3_{1\text{-}27}$K18AcK23Ac	4	5
	25	**UB8**	$H3_{1\text{-}27}K9Me_3$K18AcK23Ac	4	5
	25	**UB9**	$H3_{1\text{-}27}$	8	5
TRIM33α	500	**B1**	$H3_{1\text{-}27}K9Me_3$	15	5
	500	**B2**	$H3_{1\text{-}27}$K18Ac	15	5
	250	**B3**	$H3_{1\text{-}27}$K23Ac	4	5
	250	**B4**	$H3_{1\text{-}27}K9Me_3$K18Ac	8	5
	250	**B5**	$H3_{1\text{-}27}K9Me_3$K23Ac	4	5
	250	**B6**	$H3_{5\text{-}24}$K14Ac	30	5
	250	**B7**	$H3_{1\text{-}27}$K14Ac	15	5
	250	**UB7**	$H3_{1\text{-}27}$K18AcK23Ac	8	5
	250	**UB8**	$H3_{1\text{-}27}K9Me_3$K18AcK23Ac	4	5
	250	**UB9**	$H3_{1\text{-}27}$	8	5
TRIM33β	125	**B1**	$H3_{1\text{-}27}K9Me_3$	8	5
	125	**B2**	$H3_{1\text{-}27}$K18Ac	15	5
	125	**B3**	$H3_{1\text{-}27}$K23Ac	4	5
	125	**B4**	$H3_{1\text{-}27}K9Me_3$K18Ac	8	5
	125	**B5**	$H3_{1\text{-}27}K9Me_3$K23Ac	4	5
	125	**B6**	$H3_{5\text{-}24}$K14Ac	15	5
	125	**B7**	$H3_{1\text{-}27}$K14Ac	15	5
	125	**UB7**	$H3_{1\text{-}27}$K18AcK23Ac	8	5
	125	**UB8**	$H3_{1\text{-}27}K9Me_3$K18AcK23Ac	4	5
	125	**UB9**	$H3_{1\text{-}27}$	8	5

Peptides

Peptides were supplied as 1–9 mg quantities with >95% purity (as determined by HPLC; Cambridge Research Biochemicals or Genscript). Residual water and counter ions were predicted to comprise 20% of the net mass, and peptides were dissolved to 10, 1.0, and 0.1 mM stocks in buffer (50 mM HEPES, 500 mM NaCl; pH 7.6) before being stored at −80 °C in 5 µL aliquots. A terminal tyrosine residue was incorporated into the peptide structure to allow for accurate quantification of concentration.

Table S15. Sequences of unbiotinylated peptides.

Peptide	Sequence	Amino acid sequence
U1	$H3_{1\text{-}27}K14Ac$	ARTKQTARKSTGGK(Ac)APRKQLATKAARKY
U2	$H3_{1\text{-}27}K18Ac$	ARTKQTARKSTGGKAPRK(Ac)QLATKAARKY
U3	$H3_{1\text{-}27}K23Ac$	ARTKQTARKSTGGKAPRKQLATK(Ac)AARKY
U4	$H3_{1\text{-}27}K9Me3$	ARTKQTARK(Me_3)STGGKAPRKQLATKAARKY
U5	$H3_{1\text{-}27}K9Me3K18Ac$	ARTKQTARK(Me_3)STGGKAPRK(Ac)QLATKAARKY
U6	$H3_{1\text{-}27}K9Me3K23Ac$	ARTKQTARK(Me_3)STGGKAPRKQLATK(Ac)AARKY
U7	$H3_{1\text{-}27}K18AcK23Ac$	ARTKQTARKSTGGKAPRK(Ac)QLATK(Ac)AARKY
U8	$H3_{1\text{-}27}K9Me3K18AcK23Ac$	ARTKQTARK(Me_3)STGGKAPRK(Ac)QLATK(Ac)AARKY
U9	$H3_{1\text{-}27}$	ARTKQTARKSTGGKAPRKQLATKAARKY
U10	$H3_{5\text{–}24}K14Ac$	QTARKSTGGK(Ac)APRKQLATKAY

Figure S1. Peptide binding curves from AlphaScreen (data shown in Table 1)

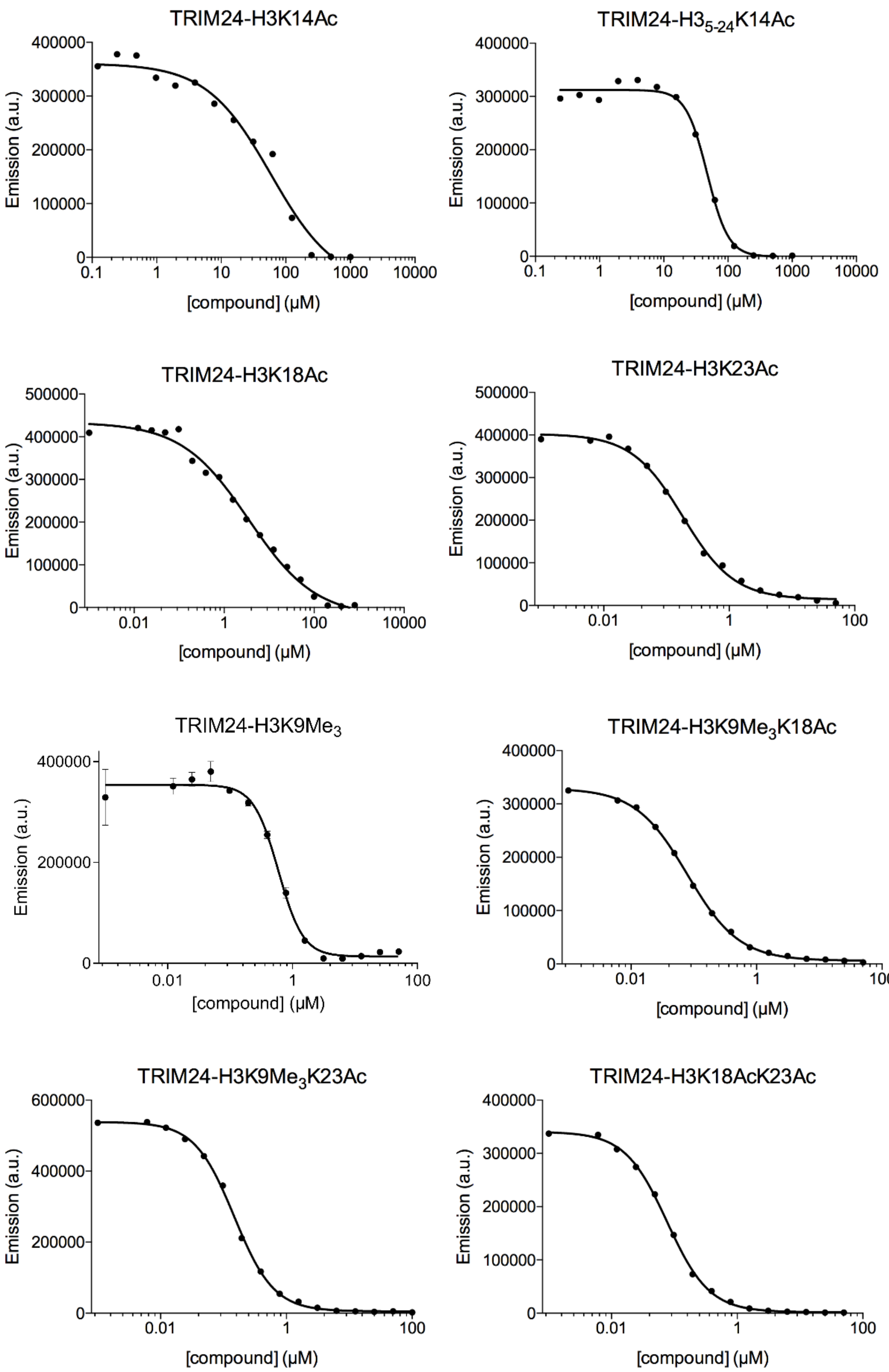

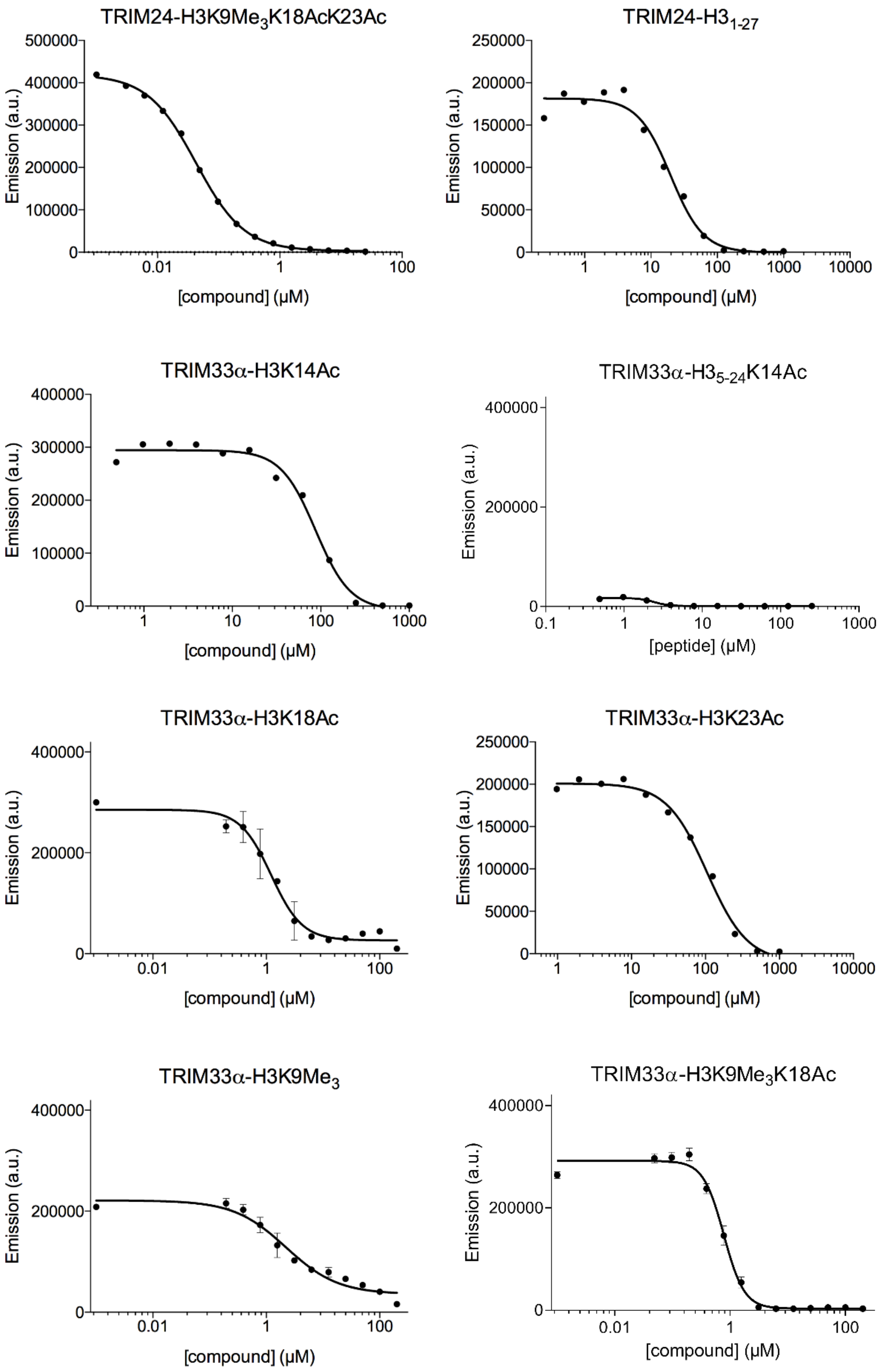

TRIM24-H3K9Me3K18AcK23Ac
TRIM24-H31-27
TRIM33α-H3K14Ac
TRIM33α-H35-24K14Ac
TRIM33α-H3K18Ac
TRIM33α-H3K23Ac
TRIM33α-H3K9Me3
TRIM33α-H3K9Me3K18Ac
Emission (a.u.)
[compound] (μM)
[peptide] (μM)

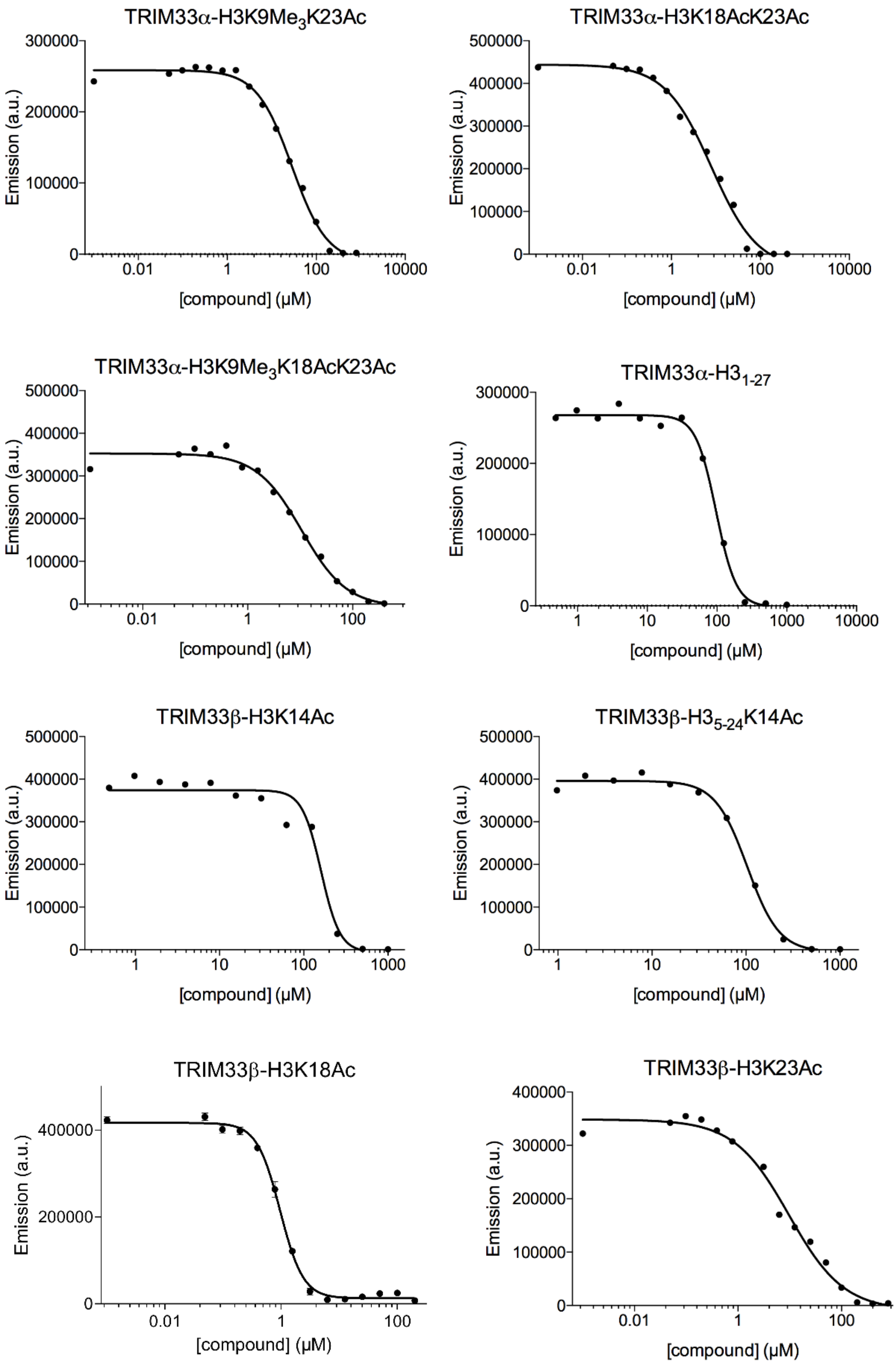
TRIM33α-H3K9Me3K23Ac
TRIM33α-H3K18AcK23Ac
TRIM33α-H3K9Me3K18AcK23Ac
TRIM33α-H31-27
TRIM33β-H3K14Ac
TRIM33β-H35-24K14Ac
TRIM33β-H3K18Ac
TRIM33β-H3K23Ac
Emission (a.u.)
[compound] (μM)

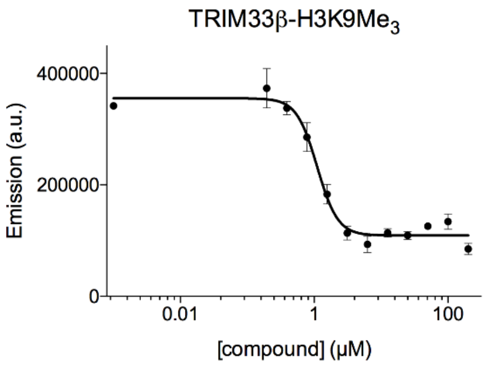
TRIM33β-H3K9Me3
Emission (a.u.)
400000
200000
0
0.01
1
100
[compound] (μM)

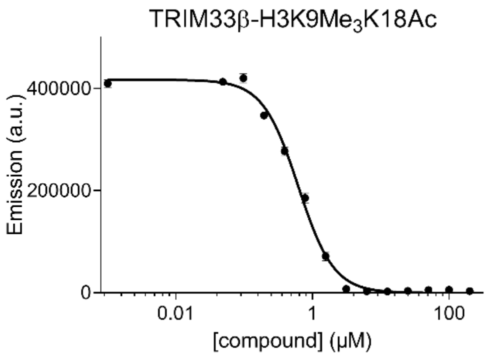
TRIM33β-H3K9Me3K18Ac
Emission (a.u.)
400000
200000
0
0.01
1
100
[compound] (μM)

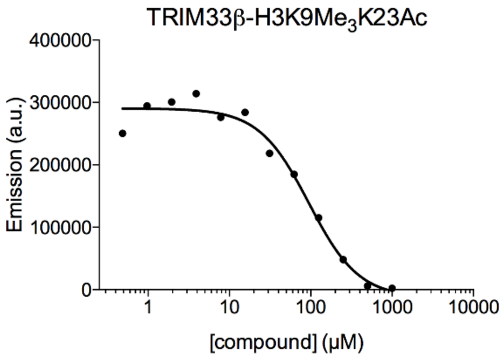
TRIM33β-H3K9Me3K23Ac
Emission (a.u.)
400000
300000
200000
100000
0
1
10
100
1000
10000
[compound] (μM)

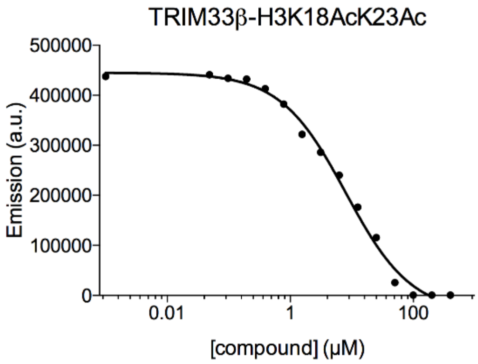
TRIM33β-H3K18AcK23Ac
Emission (a.u.)
500000
400000
300000
200000
100000
0
0.01
1
100
[compound] (μM)

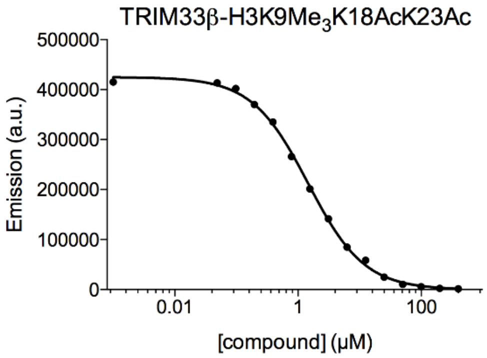
TRIM33β-H3K9Me3K18AcK23Ac
Emission (a.u.)
500000
400000
300000
200000
100000
0
0.01
1
100
[compound] (μM)

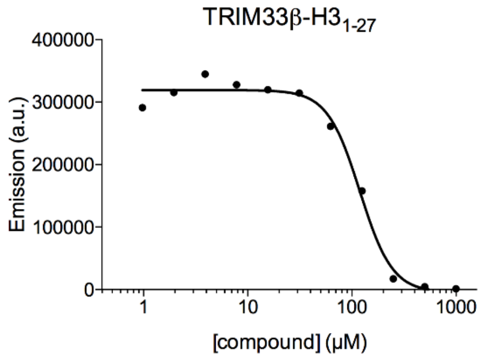
TRIM33β-H31-27
Emission (a.u.)
400000
300000
200000
100000
0
1
10
100
1000
[compound] (μM)

Figure S2. Mutant Protein AlphaScreen.

Percentage activity was calculated using the following equation:[3]

$$\%\,\boldsymbol{activity} = \mathbf{100} \times \frac{\boldsymbol{Emission(compound) - Emission\ (average\ background)}}{\boldsymbol{Emission(control) - Emission\ (average\ background)}}$$

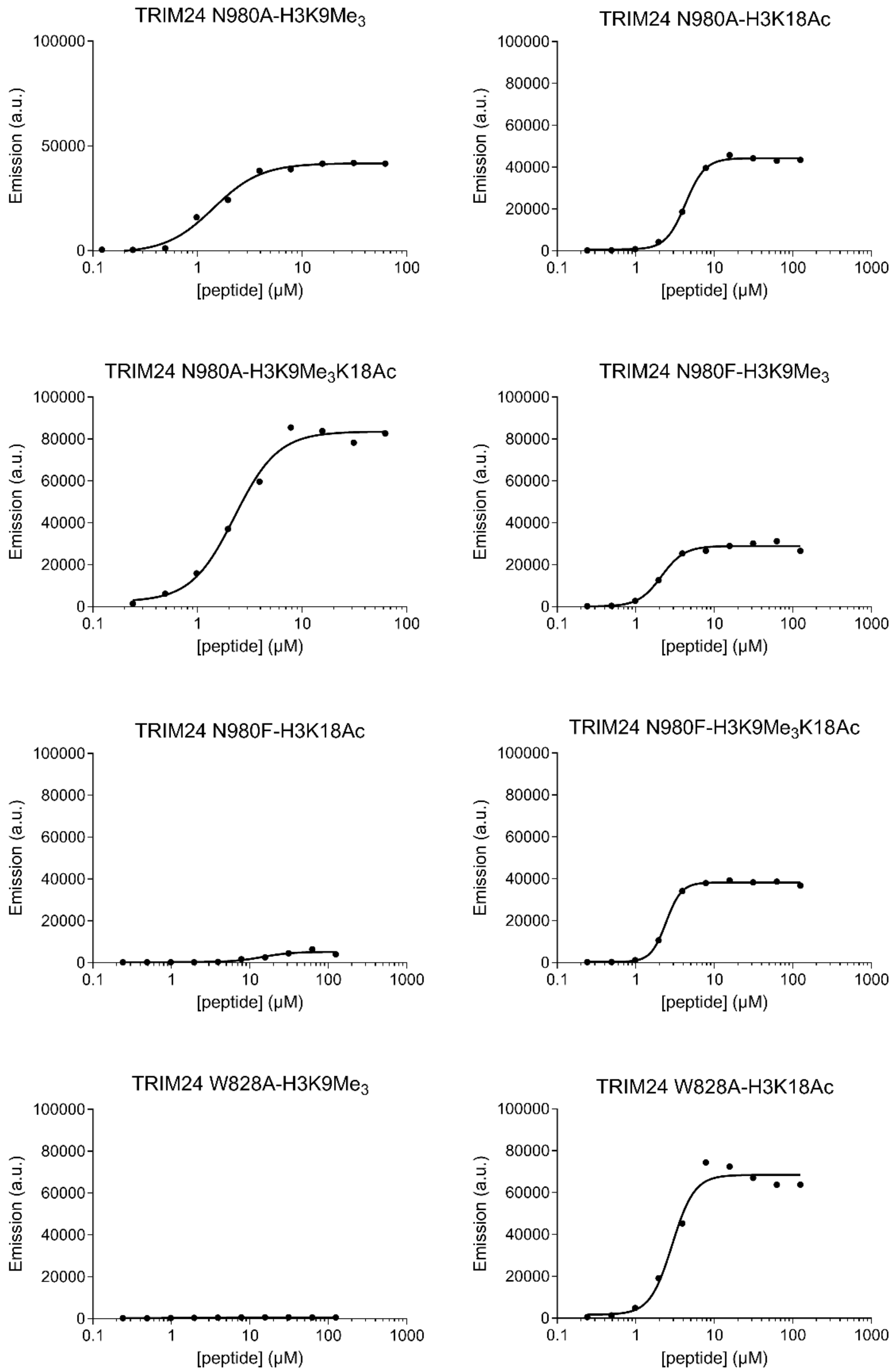

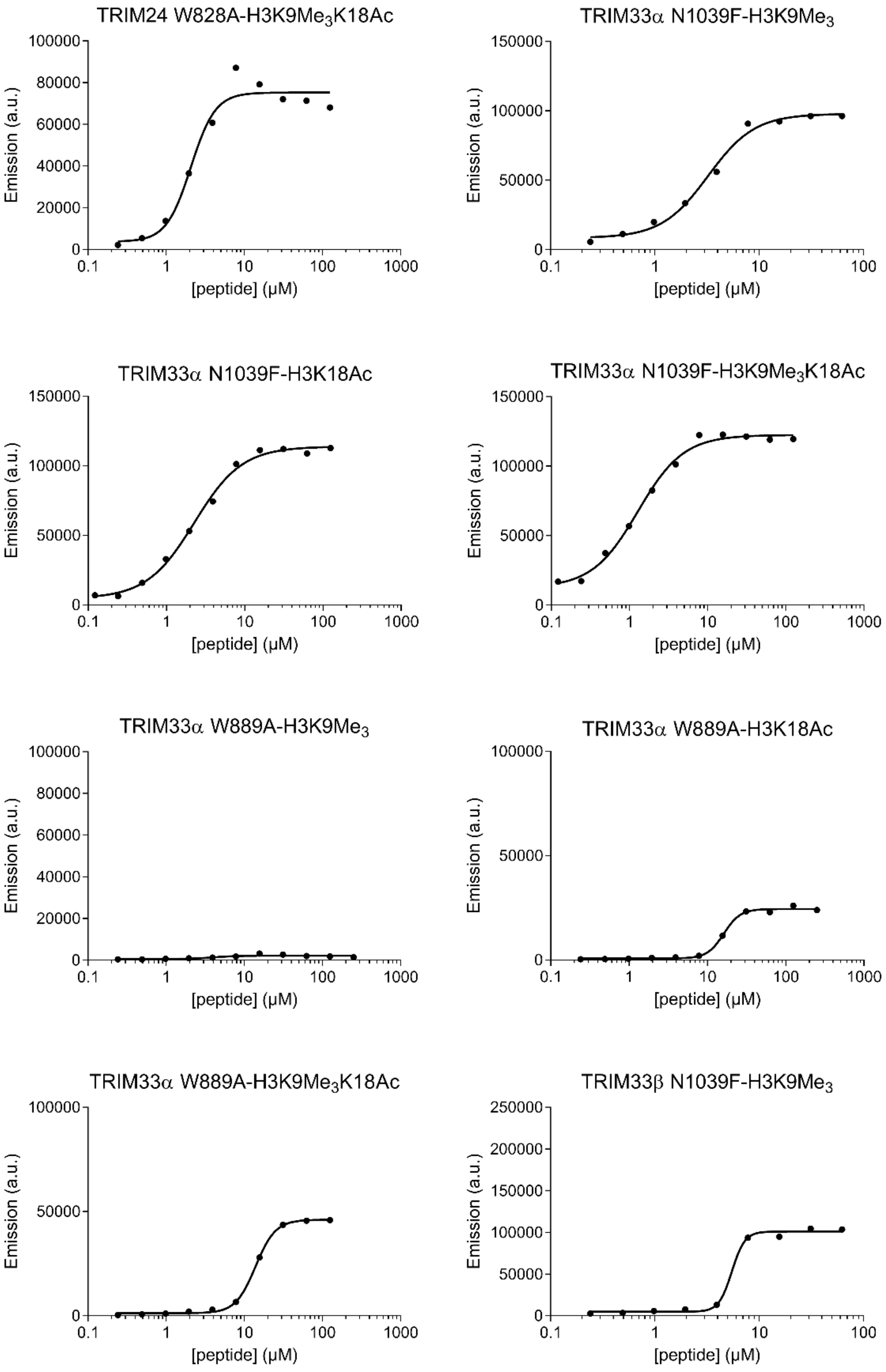
TRIM24 W828A-H3K9Me3K18Ac
TRIM33α N1039F-H3K9Me3
TRIM33α N1039F-H3K18Ac
TRIM33α N1039F-H3K9Me3K18Ac
TRIM33α W889A-H3K9Me3
TRIM33α W889A-H3K18Ac
TRIM33α W889A-H3K9Me3K18Ac
TRIM33β N1039F-H3K9Me3
Emission (a.u.)
[peptide] (μM)

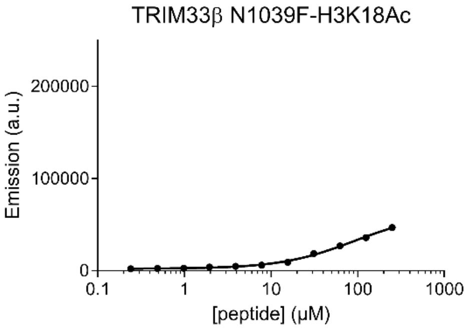
TRIM33β N1039F-H3K18Ac
Emission (a.u.)
200000
100000
0
0.1
1
10
100
1000
[peptide] (μM)

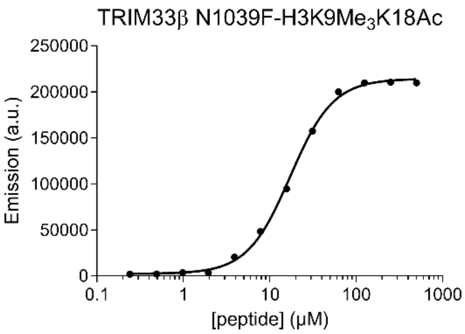
TRIM33β N1039F-H3K9Me3K18Ac
Emission (a.u.)
250000
200000
150000
100000
50000
0
0.1
1
10
100
1000
[peptide] (μM)

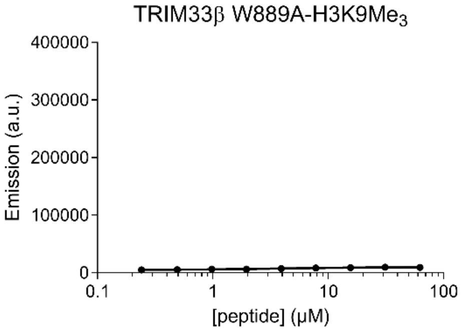
TRIM33β W889A-H3K9Me3
Emission (a.u.)
400000
300000
200000
100000
0
0.1
1
10
100
[peptide] (μM)

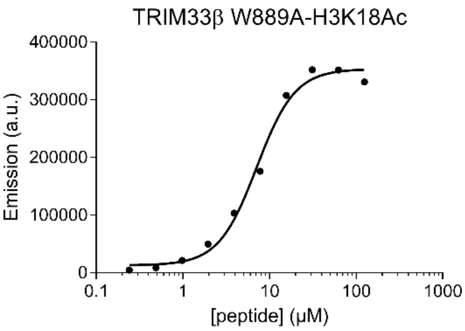
TRIM33β W889A-H3K18Ac
Emission (a.u.)
400000
300000
200000
100000
0
0.1
1
10
100
1000
[peptide] (μM)

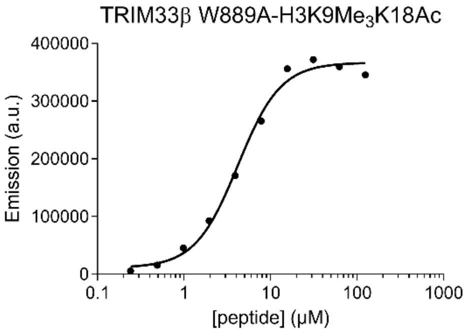
TRIM33β W889A-H3K9Me3K18Ac
Emission (a.u.)
400000
300000
200000
100000
0
0.1
1
10
100
1000
[peptide] (μM)

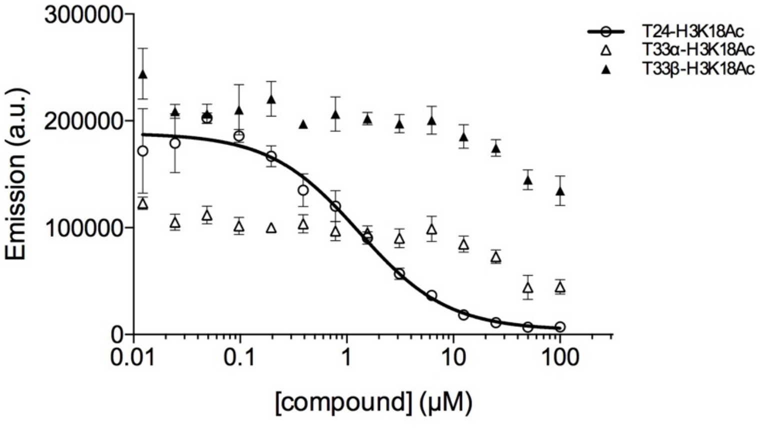
Emission (a.u.)
300000
200000
100000
0
0.01
0.1
1
10
100
[compound] (μM)
T24-H3K18Ac
T33α-H3K18Ac
T33β-H3K18Ac

Compound Screening

Compounds were provided in, or transferred to, LabCyte 384-LDV source plates, which were compatible with an Echo acoustic liquid handler (Labcyte), and stored in controlled environments under nitrogen with <0.5% O_2 and <0.5% humidity when not in use. All transfers to assay plates were backfilled with DMSO to the same final volume (100 nL). Assays was performed according to the general procedure.

201 compounds from the Maybridge collection were provided as solubilised stock (30 mM in DMSO) on LabCyte 384-LDV source plates. Samples of 20 or 100 nL were transferred to assay plates in duplicate to test at 30 or 150 μM FAC. 16 compounds were subsequently provided as solid samples of 1-2 mg, and solubilised in DMSO (50 mM; c07 – 40 mM; c14 – 30 mM). Titrations were manually prepared using a Matrix electronic multichannel pipette (Thermo Scientific) as dilutions in fresh DMSO from stock concentrations, and transferred to LabCyte 384-LDV source plates. 1536 compounds from the PPI-net compound collection were provided (10 mM in DMSO) in 96-well V-bottomed microplates (Greiner). These compounds were first transferred to 20 × LabCyte 384-LDV plates for use with the Echo acoustic liquid handler (Labcyte) using a Bravo Automated Liquid-Handling Platform (Agilent). Samples of 20 or 100 nL were transferred to assay plates in duplicate to test at 10 or 50 μM FAC.

1.7.1 AlphaScreen™ TruHits

Compounds (5 μL) were dispensed into wells as serial 1:2 dilutions from the specified concentrations from 50 mM DMSO stock solutions. Assay beads (15 μL) were pre-incubated for 30 minutes and added to the first plate to attain a final assay volume of 20 μL, and then incubated for a further 10 minutes. To the second plate, streptavidin donor beads (7.5 μL) were added to the compounds and incubated for 30 minutes. Biotinylated acceptor beads (7.5 μL) were then added to the second plate to attain a final assay volume of 20 μL, and then incubated for a further 30 minutes. Data were processed by calculating the percentage decrease in emission intensity with respect to the average of the DMSO control wells on each respective plate, which were set as 100%.

1.7.2 Isothermal Titration Calorimetry (ITC)

All calorimetric experiments were performed on a MicroCal iTC200 or a MicroCal PEAQ-ITC Automated (Malvern) and analysed with the MicroCal PEAQ-ITC Analysis software (Malvern 1.1.0.1262) using a single binding site model. The first data point was excluded from the analysis. Proteins and peptides containing a C-terminal tyrosine were dialysed at 4 °C overnight in a Slide-A-Lyzer™ MINI Dialysis Device (2000 MWCO; Thermo Scientific Life Technologies) into 50 mM HEPES, 150 mM NaCl; pH 7.4. Samples were centrifuged to remove aggregates (2 min, 3000 rpm, 25 °C), and concentrations were determined by measuring the absorbance at 280 nm using a Nanodrop® ND-1000 spectrophotometer (Nanodrop® Technologies Inc.) with the 'Protein A280' program module according to the manufacturer's instructions (tyrosine ε_{280} = 1490 $M^{-1}cm^{-1}$). Samples were diluted to the required concentration using dialysis buffer. The cell was stirred at 750 rpm, with reference power set to 5 µcal/sec and temperature held at 298 K. After an initial delay of 60 sec, 20×2 µL injections (first injection 0.4 µL) were performed with a spacing of 150 sec. Heats of dilution were measured under the same conditions and subtracted for analysis.

Protein solutions in the calorimetric cell (420 µL) were titrated with the peptide solutions in the syringe (150 µL).

Table S16. Concentrations used for ITC. Protein concentrations are in bold, and peptide concentration are not bold.

	$H3_{1-27}K9Me_3$	$H3_{1-27}K18Ac$	$H3_{1-27}K9Me_3K18Ac$
**TRIM24**	**50 µM**, 500 µM	**50 µM**, 750 µM	**50 µM**, 750 µM
**TRIM33α**	**100 µM**, 1000 µM	**100 µM**, 1000 µM	**100 µM**, 1000 µM
**TRIM33β**	**90 µM**, 900 µM	**90 µM**, 900 µM	**90 µM**, 900 µM

1.7.3 *ITC data for H3K9Me$_3$, H3K18Ac, H3K9Me$_3$K18Ac*

Table S17. Raw data for ITC of TRIM24, TRIM33α, and TRIM33β with modified peptides.

	**H3K9Me$_3$**	**H3K18Ac**	**H3K9Me$_3$K18Ac**
TRIM24	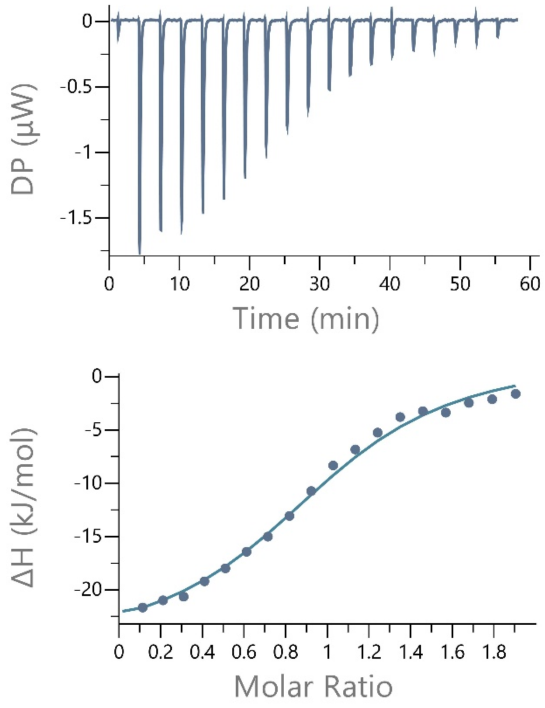	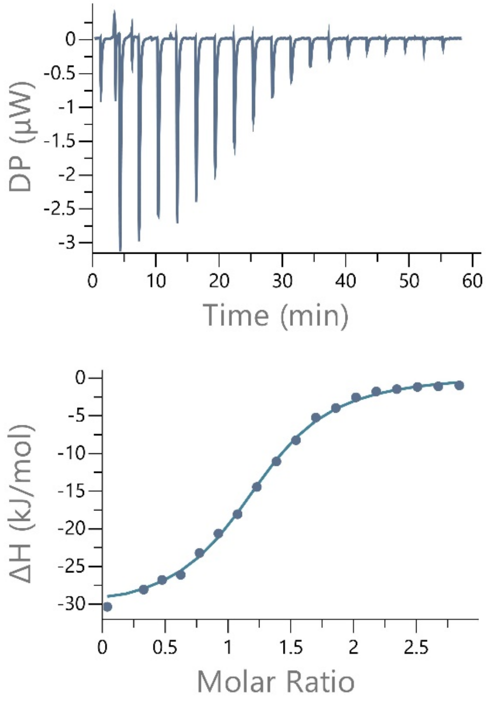	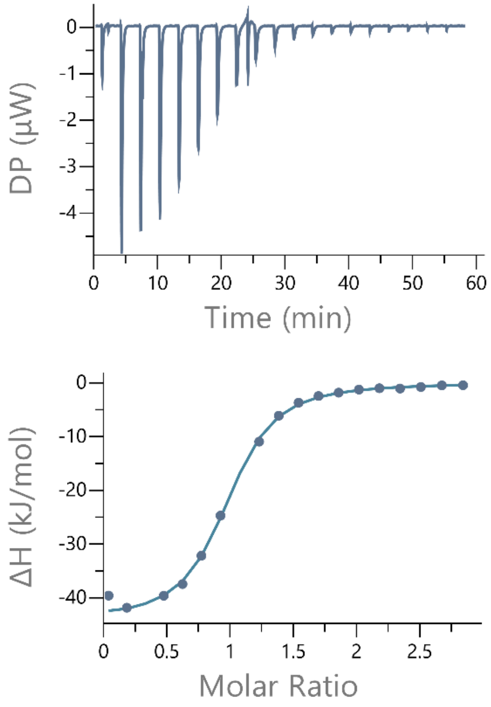

TRIM33

α

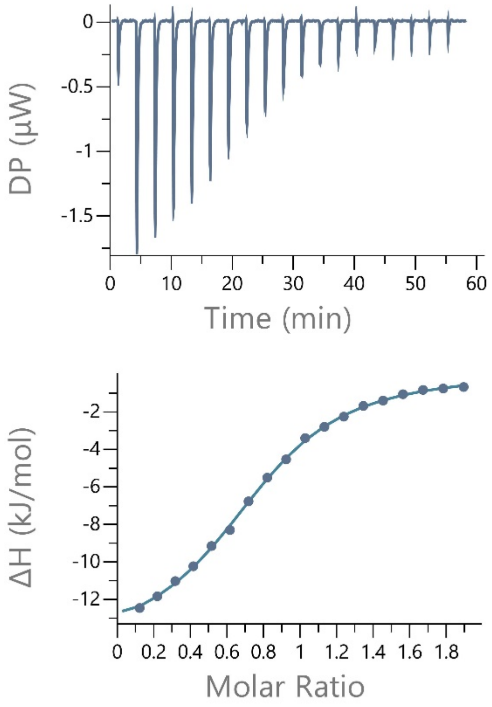
DP (μW)
Time (min)
ΔH (kJ/mol)
Molar Ratio

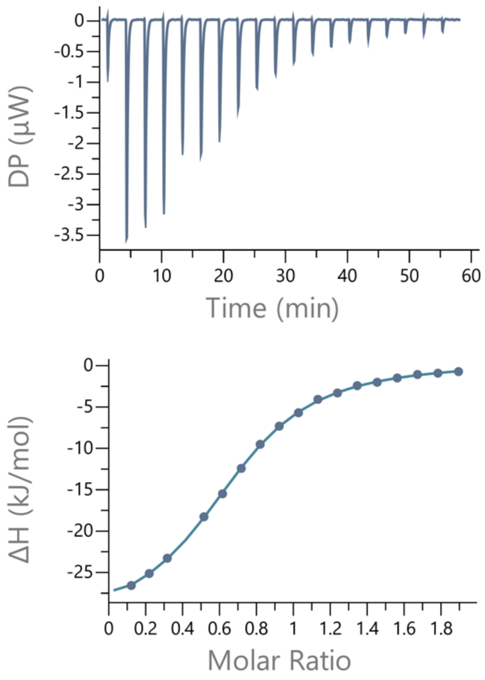
DP (μW)
Time (min)
ΔH (kJ/mol)
Molar Ratio

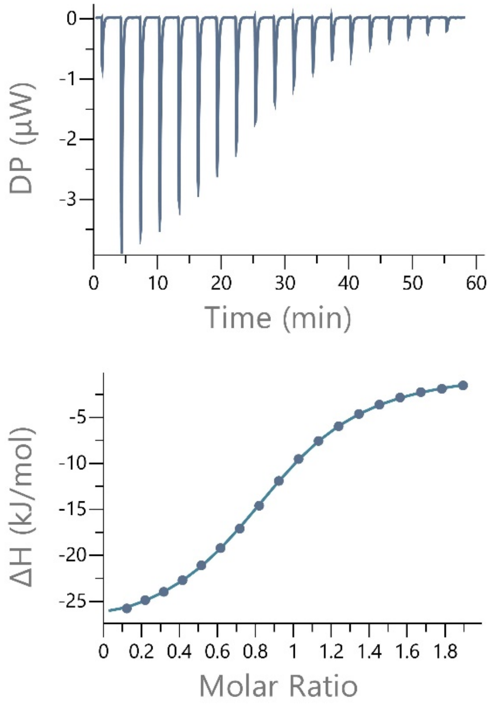
DP (μW)
Time (min)
ΔH (kJ/mol)
Molar Ratio

TRIM33 β

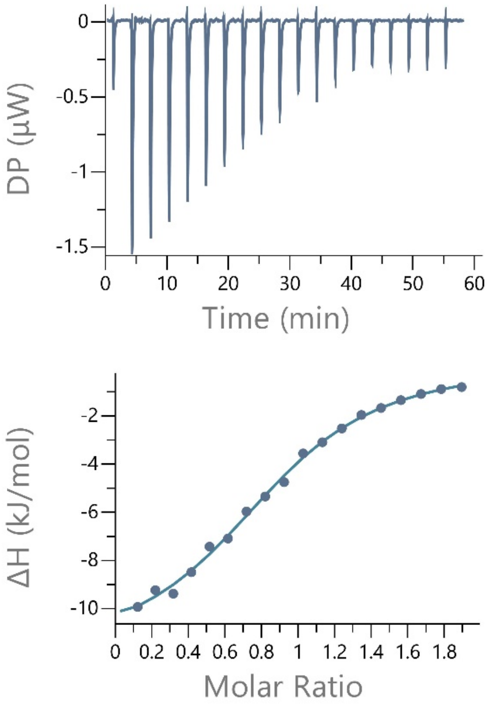
DP (μW)
Time (min)
ΔH (kJ/mol)
Molar Ratio

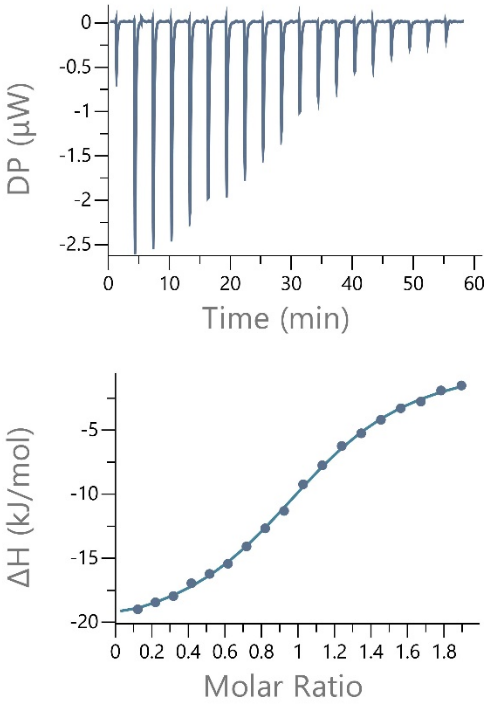
DP (μW)
Time (min)
ΔH (kJ/mol)
Molar Ratio

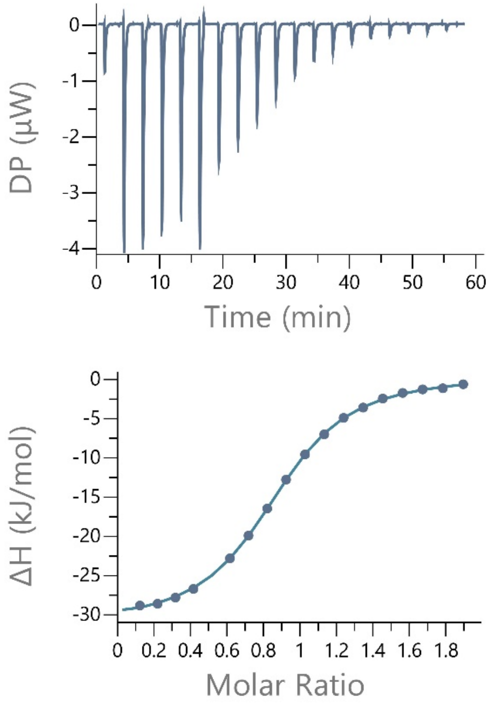
DP (μW)
Time (min)
ΔH (kJ/mol)
Molar Ratio

Table S18. ITC experimental values.

		$H3K9Me_3$	H3K18Ac	$H3K9Me_3K18Ac$
TRIM24	N	0.892	1.21	0.939
	K_D	3.90 ± 0.423 μM	4.37 ± 0.430 μM	1.94 ± 0.111 μM
	ΔH (kJ/mol)	-23.1 ± 0.628	-31.8 ± 0.745	-44.3 ± 0.447
	ΔG (kJ/mol)	-30.9	-30.6	-32.6
	-TΔS (kJ/mol)	-7.82	1.14	11.6
TRIM33α	N	0.757	0.670	0.883
	K_D	11.2 ± 0.760 μM	10.0 ± 0.319 μM	9.28 ± 0.214 μM
	ΔH (kJ/mol)	-14.7 ± 0.295	-31.9 ± 0.286	-29.2 ± 0.182
	ΔG (kJ/mol)	-28.3	-28.6	-28.8
	-TΔS (kJ/mol)	-13.6	3.35	0.427
TRIM33β	N	0.893	1.03	0.868
	K_D	9.81 ± 2.33 μM	8.95 ± 0.643 μM	4.78 ± 0.200 μM
	ΔH (kJ/mol)	-10.7 ± 0.735	-21.2 ± 0.440	-31.7 ± 0.279
	ΔG (kJ/mol)	-28.6	-28.9	-30.4
	-TΔS (kJ/mol)	-17.9	-7.61	1.29

1.7.4 WaterLOGSY

Table S19. WaterLOGSY buffer composition (10 mM Na/K phosphate buffer, PBS, pH 7.6).

Reagent	Amount required per litre (g)
KH_2PO_4	0.26
Na_2HPO_4	1.15
NaCl	8.71

The buffer was filtered under vacuum through a 0.2 µm filter paper (Sartorius UK) before use and stored at 4 °C. Proteins were buffer exchanged from HEPES storage buffer into PBS buffer (0.9% NaCl, pH 7.6) using GE Healthcare PD SpinTrap G-25 columns. For each compound, a water-suppressed ^{1}H spectrum was recorded as a reference, followed by waterLOGSY spectra of the compound in the absence and presence of the specified protein. Samples were prepared to a total sample volume of 160 µL in PBS buffer (pH 7.6), with 500 µM compound (unless otherwise specified) from 50 mM DMSO-D_6 stock solutions, 10% D_2O for signal locking, and 20 µM protein (unless otherwise specified) for samples with protein. After loading into 3 mm Hilgenberg NMR tubes for Bruker Match™ system, samples were briefly centrifuged in a manual benchtop rotor. Spectra were recorded on a Bruker AVIII 700 MHz spectrometer with inverse TCI cryoprobe using a standard waterLOGSY pulse sequence.[4] Peaks were assigned using DMSO-D_6 as a reference peak at 2.5 ppm.

Mutant WaterLOGSY for Screening Hits

Mutant waterLOGSY studies were performed under the same conditions as the waterLOGSY experiment described above, using a Bruker AVIII 700 MHz spectrometer.

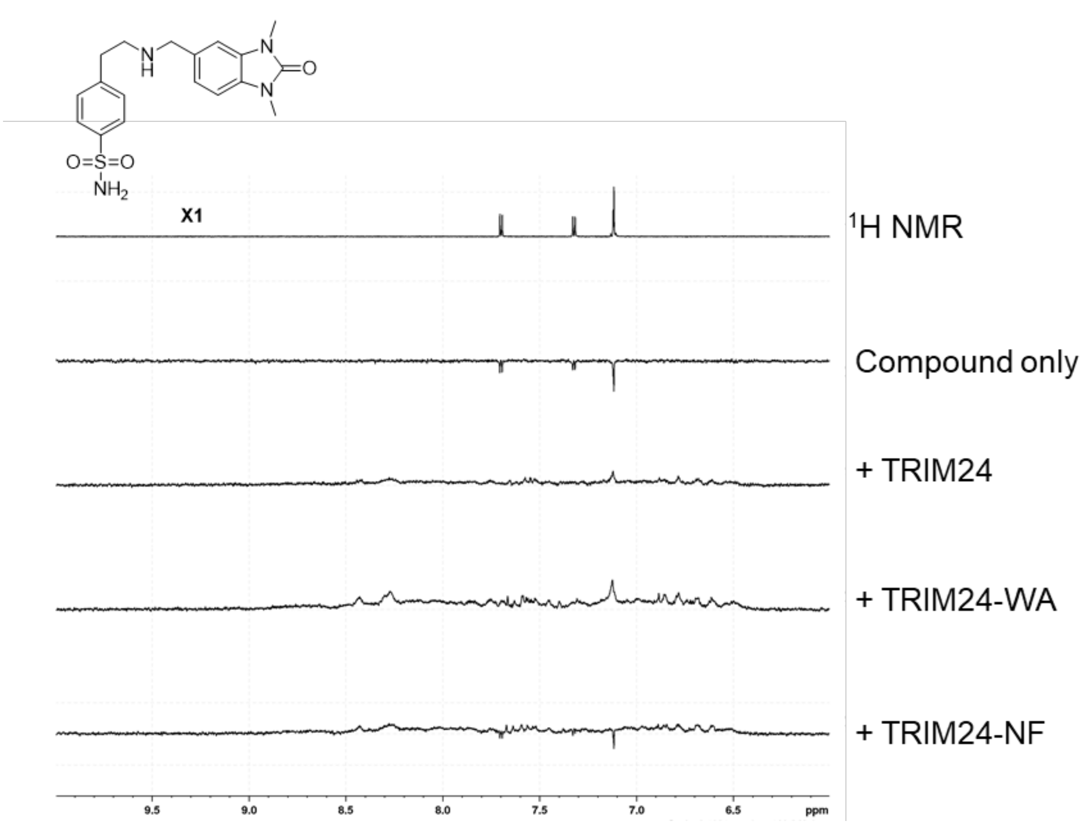
X1
^{1}H NMR
Compound only
+ TRIM24
+ TRIM24-WA
+ TRIM24-NF
9.5
9.0
8.5
8.0
7.5
7.0
6.5
ppm

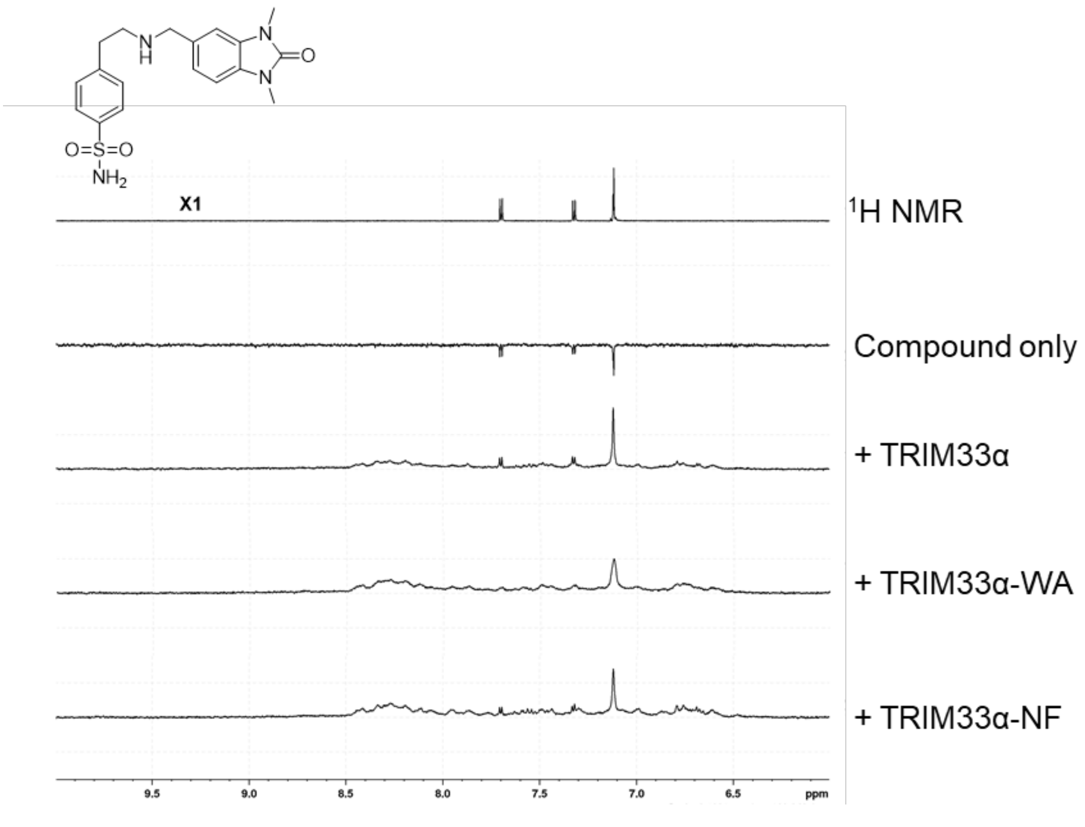
X1
^{1}H NMR
Compound only
+ TRIM33α
+ TRIM33α-WA
+ TRIM33α-NF
9.5
9.0
8.5
8.0
7.5
7.0
6.5
ppm

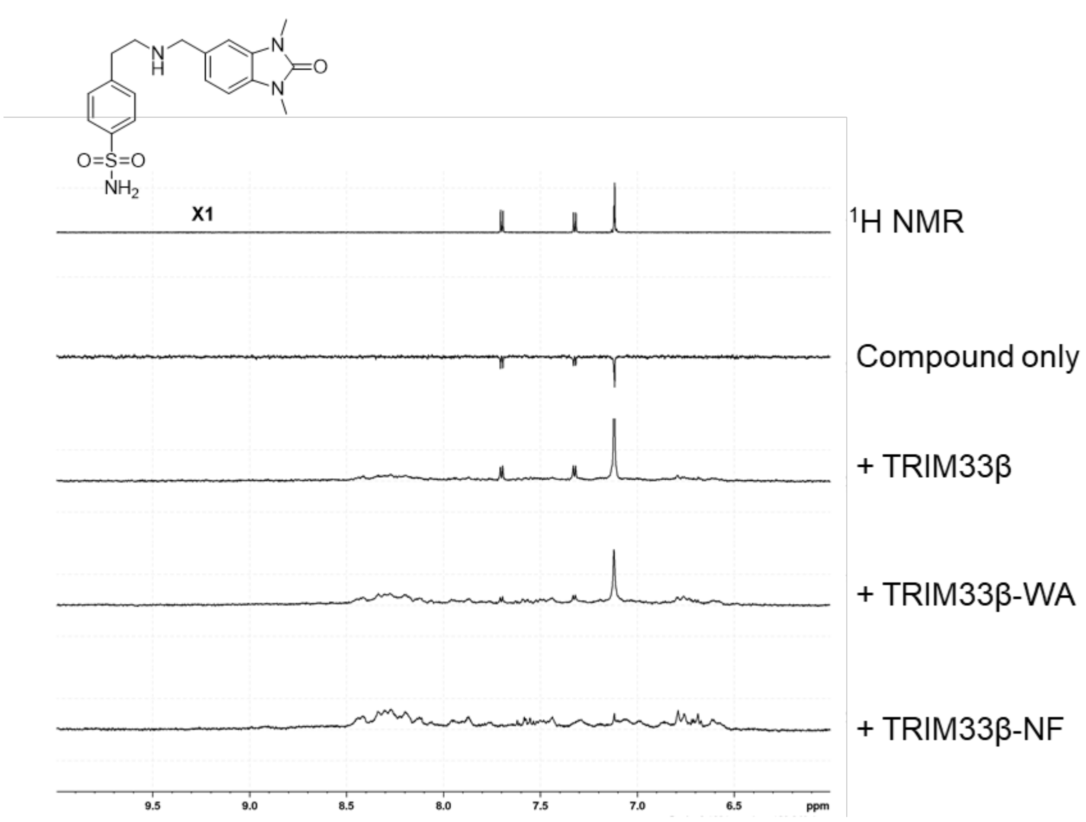
X1
1H NMR
Compound only
+ TRIM33β
+ TRIM33β-WA
+ TRIM33β-NF
9.5
9.0
8.5
8.0
7.5
7.0
6.5
ppm

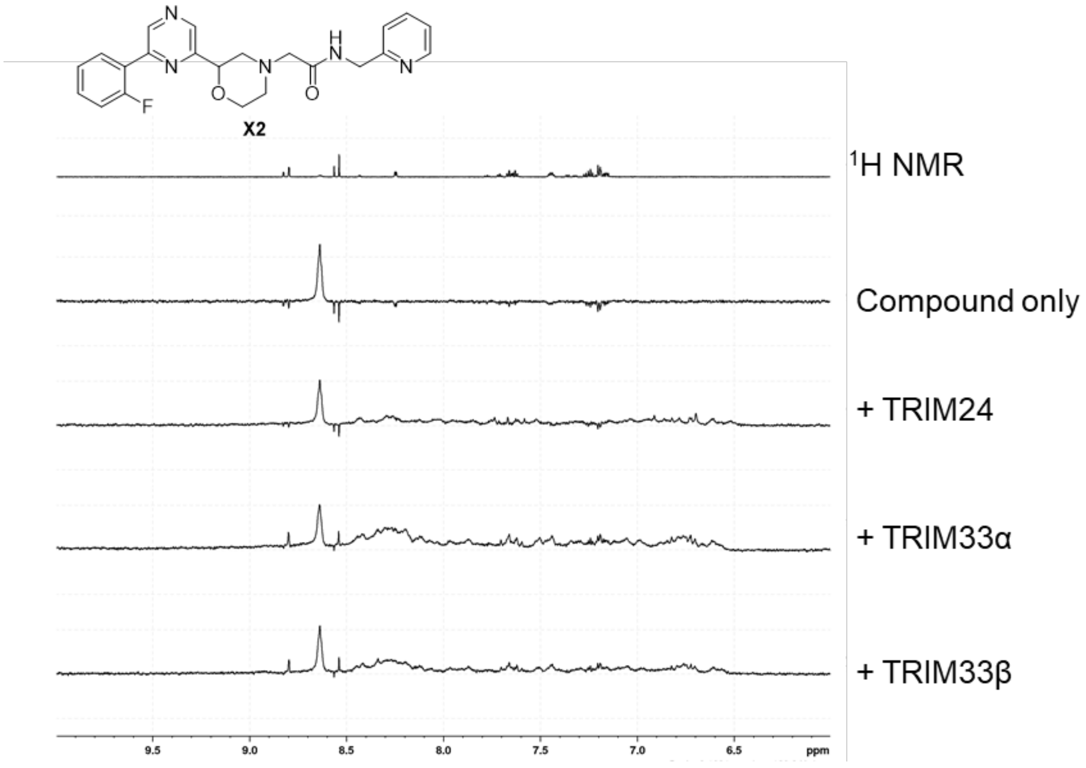
X2
1H NMR
Compound only
+ TRIM24
+ TRIM33α
+ TRIM33β
9.5
9.0
8.5
8.0
7.5
7.0
6.5
ppm

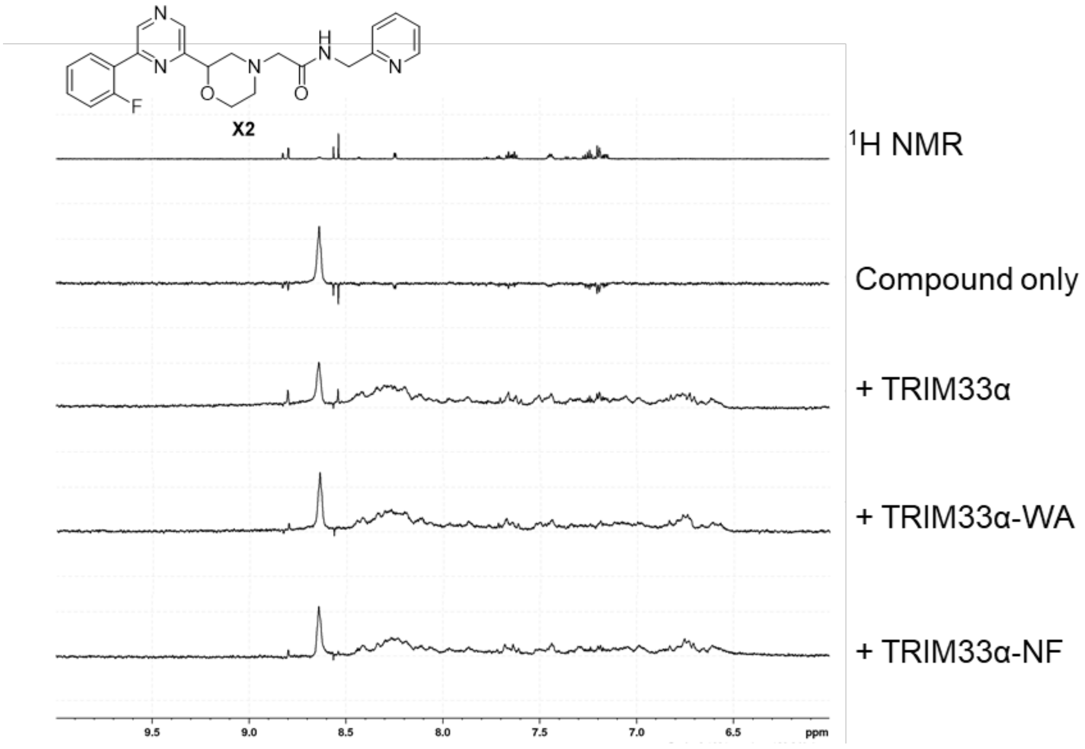
N
N
H
N
N
F
O
N
O
X2
¹H NMR
Compound only
+ TRIM33α
+ TRIM33α-WA
+ TRIM33α-NF
9.5
9.0
8.5
8.0
7.5
7.0
6.5
ppm

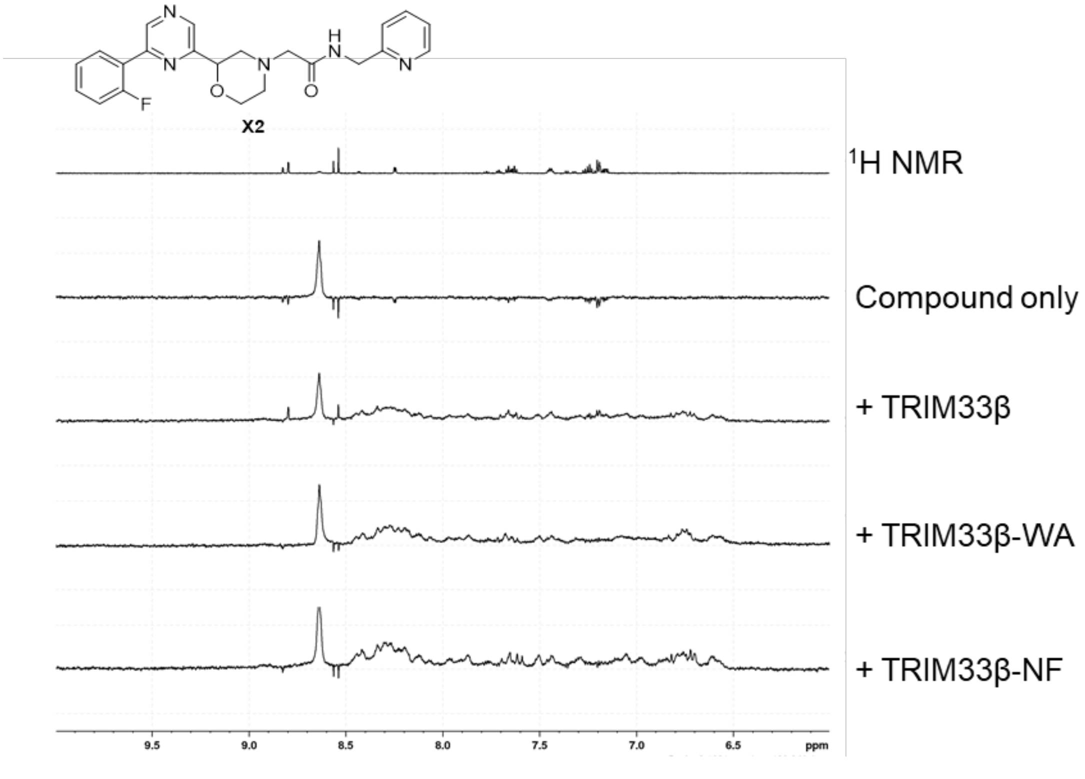
N
N
H
N
N
F
O
N
O
X2
¹H NMR
Compound only
+ TRIM33β
+ TRIM33β-WA
+ TRIM33β-NF
9.5
9.0
8.5
8.0
7.5
7.0
6.5
ppm

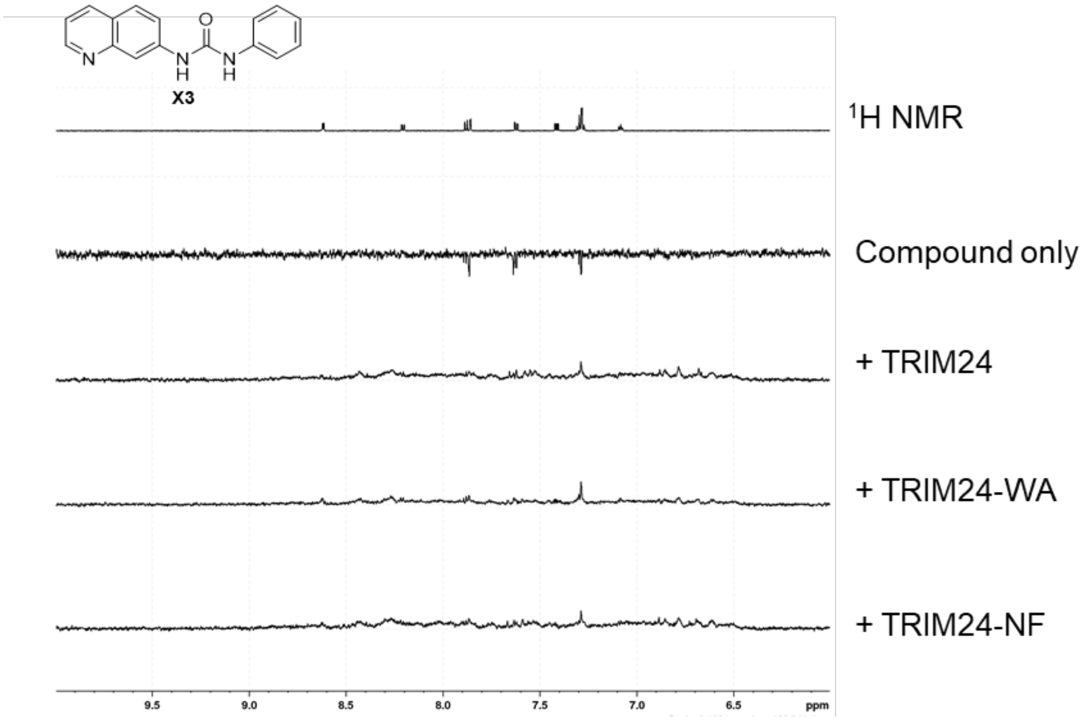

X3
^{1}H NMR
Compound only
+ TRIM24
+ TRIM24-WA
+ TRIM24-NF
9.5
9.0
8.5
8.0
7.5
7.0
6.5
ppm

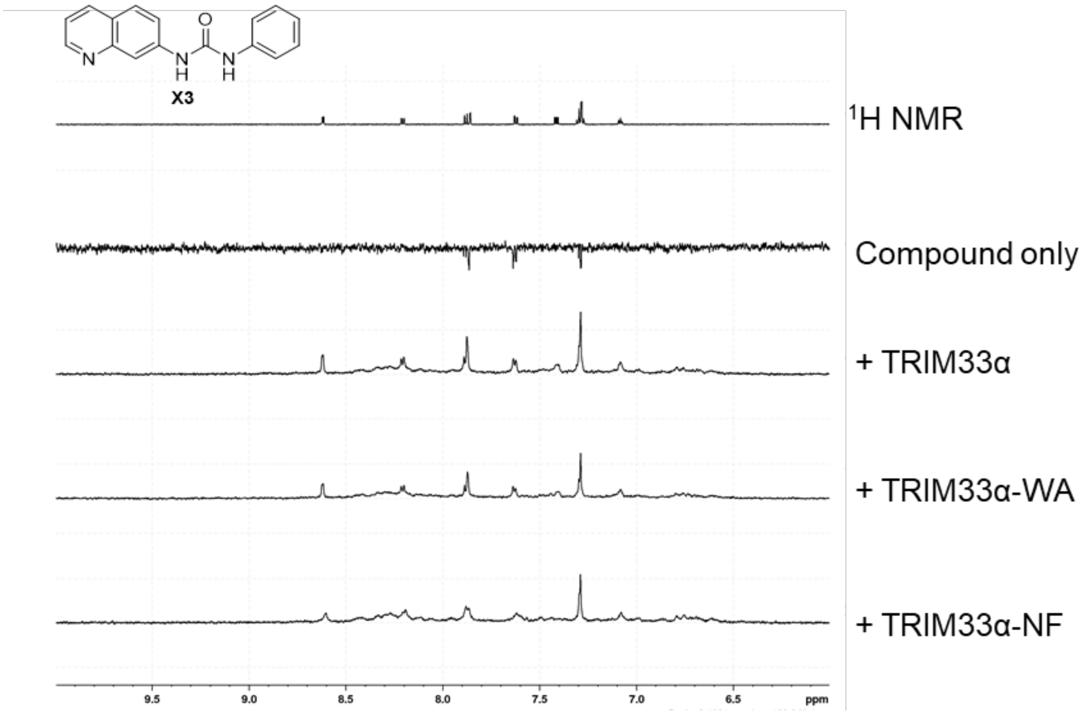

X3
^{1}H NMR
Compound only
+ TRIM33α
+ TRIM33α-WA
+ TRIM33α-NF
9.5
9.0
8.5
8.0
7.5
7.0
6.5
ppm

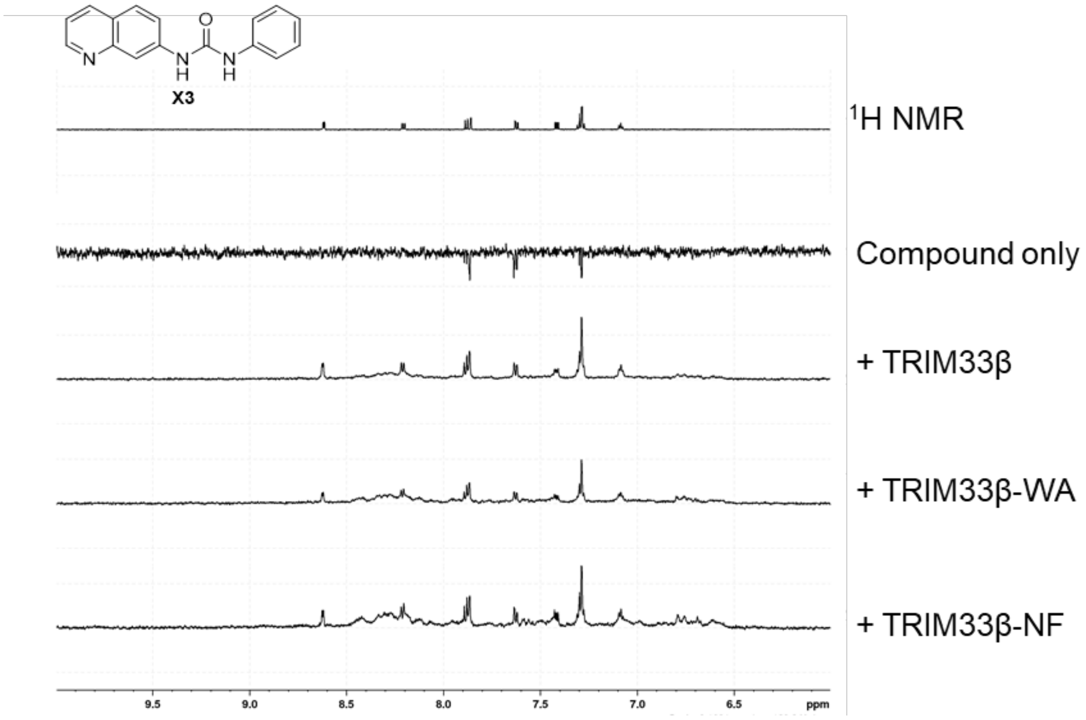
X3
¹H NMR
Compound only
+ TRIM33β
+ TRIM33β-WA
+ TRIM33β-NF
9.5
9.0
8.5
8.0
7.5
7.0
6.5
ppm

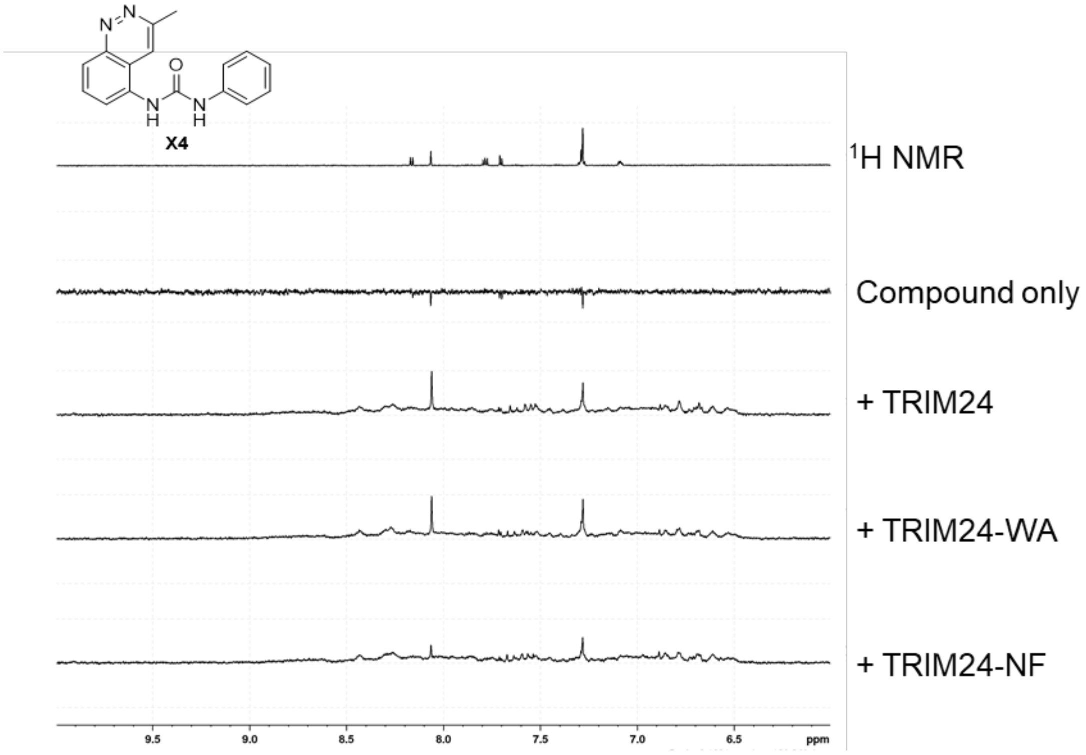
X4
¹H NMR
Compound only
+ TRIM24
+ TRIM24-WA
+ TRIM24-NF
9.5
9.0
8.5
8.0
7.5
7.0
6.5
ppm

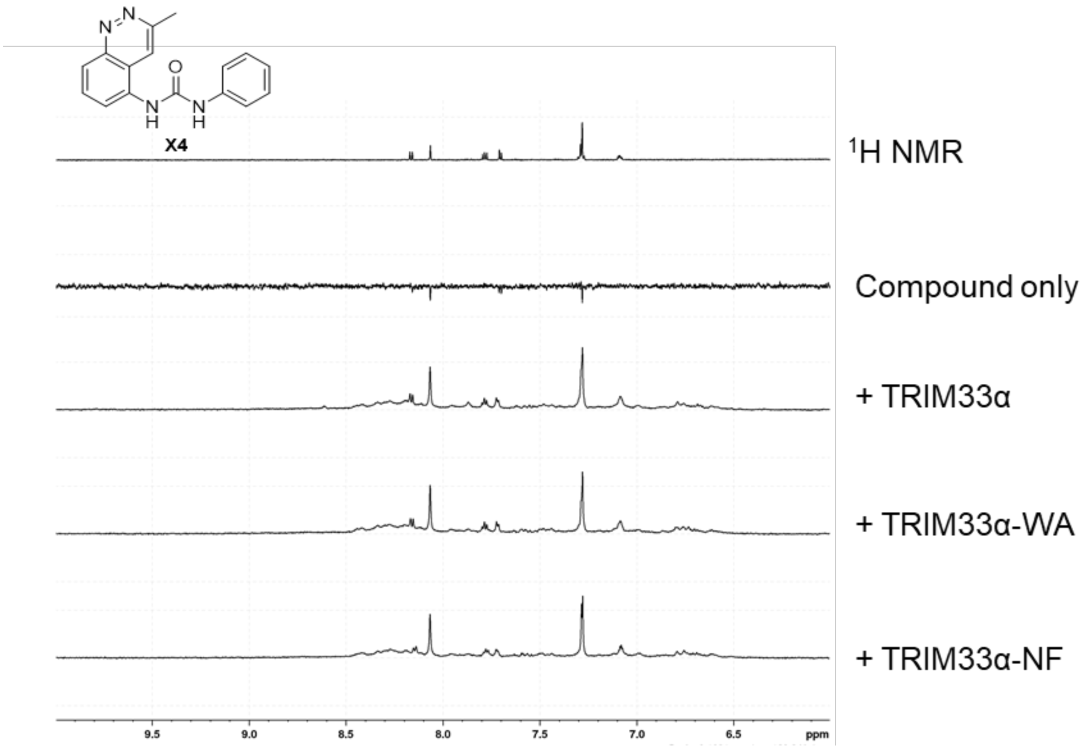
X4
¹H NMR
Compound only
+ TRIM33α
+ TRIM33α-WA
+ TRIM33α-NF
9.5
9.0
8.5
8.0
7.5
7.0
6.5
ppm

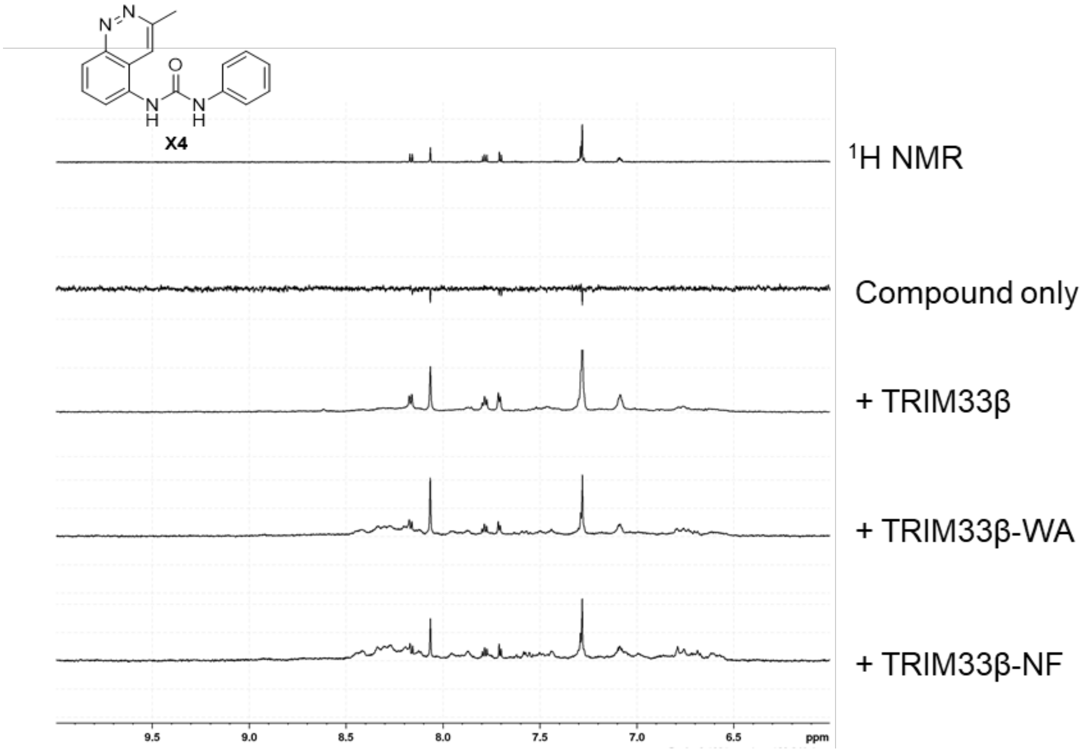
X4
¹H NMR
Compound only
+ TRIM33β
+ TRIM33β-WA
+ TRIM33β-NF
9.5
9.0
8.5
8.0
7.5
7.0
6.5
ppm

2 Chemistry Experimental Section

2.1 General Experimental Section

Reactions were carried out under a nitrogen or argon atmosphere in oven-dried or flame-dried glassware unless otherwise stated. Standard inert atmosphere techniques were used in handling all air and moisture sensitive reagents. All data were gathered at 300 K unless otherwise stated.

Solvents and reagents were used as supplied by major chemical suppliers. Petroleum ether refers to distilled light petroleum of fraction (30 °C – 40 °C). Inorganic solutions are saturated aqueous solutions unless otherwise stated. Brine refers to a saturated aqueous sodium chloride solution. Concentrations of solvents and solutions in general procedures are given relative to 1 eq of starting material.

Silica gel chromatography was carried out using VWR Kieselgel 60 silica gel (60-63 μm) under a positive pressure of nitrogen. Thin-layer chromatography (TLC) was carried out using Merck Kieselgel 60 F254 (230-400 mesh) aluminium-supported, fluorescent treated silica, visualised under UV light (λ_{max} 254 and 365 nm), followed by thermal development after dipping in one of: a) aqueous solution of ninhydrin, acetic acid, and *n*-butanol; b) aqueous solution of potassium permanganate and hydrochloric acid.

Melting points were obtained using a Gallenkamp MF-370 capillary tube melting point apparatus (Registered Design No. 889339) and are uncorrected.

Infrared spectra were recorded on a Bruker Tensor 27 FT-IR spectrometer from a thin film deposited onto a sodium chloride plate or a diamond ATR module. Only selected maximum absorbances (ν_{max}) of the most intense peaks are reported (cm^{-1}): br = broad, s = strong.

^{1}H NMR spectra were measured on a Bruker AVIII HD 400 (400 MHz), Bruker AVII 500 (500 MHz) or Bruker AVIII HD 500 (500 MHz) spectrometer in the solvent stated as a reference for the internal deuterium lock. The chemical shift data for each signal are given as δ in units of parts per million (ppm) relative to tetramethylsilane (TMS), where δ (TMS) = 0.00. The spectra are calibrated using the solvent peak with the data provided by Fulmer *et al.*[5] The number of protons (n) for a given resonance signal is

indicated by nH. The multiplicity of each signal is indicated by: s (singlet); br s (broad singlet); d (doublet); t (triplet); q (quartet); m (multiplet); or combinations thereof. Where appropriate, coupling constants (*J*) are quoted in Hz, recorded to the nearest 0.1 Hz, and were determined by analysis using Bruker TopSpin v3.2 software and MestReNova v12.0.4 software. Identical coupling constants were averaged. Spectra were assigned using COSY, HSQC, HMBC and NOE experiments, as appropriate.

^{13}C NMR spectra were measured on a Bruker AVII 500 (126 MHz) spectrometer in the solvent stated as a reference for the internal deuterium lock. The chemical shift data for each signal are given as δ in units of parts per million (ppm) relative to tetramethylsilane (TMS) where δ (TMS) = 0.00. The spectra are calibrated using the solvent peak with the data provided by Fulmer *et al.*[5] Signals are quoted to 1 decimal place unless peaks are indistinguishable, in which case 2 decimal places are used. The multiplicity of each signal is singlet unless otherwise stated. Spectra were assigned using HSQC and HMBC experiments, as appropriate.

Mass spectra were acquired on either an Agilent 6120 spectrometer (low resolution) or Bruker micrOToF spectrometer (high resolution) using electrospray ionisation (ESI) from solutions of methanol or water. *m/z* values are reported in Daltons and followed by their percentage abundance in parentheses.

Analytical high-performance liquid chromatography (HPLC) was performed on a PerkinElmer Flexar system with a Binary LC Pump and UV/VIS LC Detector set at 254 nm (unless otherwise stated). For determination of purity, a Dionex Acclaim® 120 C18 reverse phase column [5 µm, 12 Å, 150 mm × 4.6 mm] was used with a constant flow rate of 1.5 mL min^{-1} and gradient method (10 min [95:5 H_2O:acetonitrile (0.1% TFA) to 5:95 H_2O:acetonitrile (0.1% TFA)]; 5 min hold). Samples injected were prepared by dissolving in methanol, water or acetonitrile, and filtered. Samples run on an Atlantis™ C18 column are indicated. Atlantis™ reverse phase column [3 µM, 100 Å, 150 × 4.6 mm] was used with a constant flow rate of 1.0 mL min^{-1} and gradient method (2 min hold; 13 min [100:0 H_2O:acetonitrile (0.1% TFA) to 5:95 H_2O:acetonitrile (0.1% TFA)]; 5 min hold). Samples injected were prepared by dissolving in H_2O, and filtered.

2.2 Experimental Details

2-(4''-Methyl-1*H*-imidazol-5''-yl)-*N*-[3'-(morpholin-4'-yl)ethyl]imidazo[1,2-a]pyridin-3-amine (2)

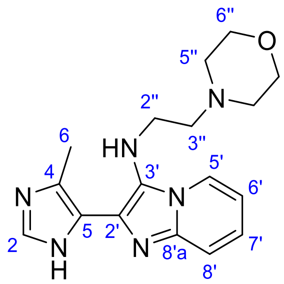

2-Aminopyridine (150 mg, 1.59 mmol, 1.0 eq.) was dissolved in anhydrous dimethyl sulfoxide (3.00 mL). 4-Methyl-5-imidazolecarboxaldehyde (263 mg, 2.39 mmol, 1.5 eq.), 2-morpholinoethyl isocyanide (255 µL, 1.85 mmol, 1.16 eq.) and scandium(III) triflate (62.7 mg, 0.127 mmol, 0.08 eq.) were added. The reaction mixture was heated to 100 °C and stirred for 17 h. After this time TLC analysis indicated complete consumption of starting material (1:9 MeOH/CH_2Cl_2). The reaction was cooled to rt and quenched with H_2O (40 mL). The reaction mixture was extracted with CH_2Cl_2 (3 × 20 mL). The organic fractions were combined, washed sequentially with a saturated aqueous solution of $NaHCO_3$ (30 mL), and brine (30 mL), dried (Na_2SO_4), filtered, and concentrated *in vacuo*. The product was purified using silica flash column chromatography (elution with 1% NH_4OH, 5–15% EtOH/$CHCl_3$ gradient) to yield the title compound as a yellow oil (100 mg, 19%): R_f 0.35 (1% NH_4OH, 10% EtOH/$CHCl_3$); $\bar{\nu}_{max}$/cm^{-1} 3108 (s), 2923 (s), 2857 (s), 2813 (s), 2360 (w), 1650 (m), 1634 (m), 1552 (m), 1500 (m), 1453 (m), 1355 (m), 1298 (m), 1276 (m), 1234 (m), 1203 (m), 1142 (m), 1115 (m), 1034 (w), 917 (m), 857 (m), 755 (m), 733 (m), 636 (w); ^{1}H NMR (500 MHz, $CDCl_3$) δ 8.03 (1H, dt, *J* 6.8, 1.3, H-5'), 7.60 (1H, s, H-2), 7.47 (1H, dd, *J* 9.1, 1.3, H-8'), 7.09 (1H, ddd, *J* 9.1, 6.7, 1.3, H-7'), 6.76 (1H, ddd, *J* 6.8, 6.7, 1.1, H-6'), 3.68 (4H, t, *J* 4.7, H-6''), 3.14 (2H, t, *J* 6.5, H-2''), 2.57 (3H, s, H-6), 2.52 – 2.40 (6H, m, H-5'', H-3''); ^{13}C NMR (126 MHz, $CDCl_3$) δ 141.5 (C-8'a), 133.3 (C-6'), 129.7 (C-4), 129.4 (C-5), 126.1 (C-2'), 125.9 (C-3'), 123.6 (C-7'), 122.4 (C-5'), 117.1 (C-8'), 111.8 (C-6'), 66.8 (C-5''), 58.0 (C-3''), 53.4 (C-6''), 43.3 (C-2''), 12.1 (C-6); HRMS *m/z* (ESI$^+$) [Found: 327.19264, $C_{17}H_{23}ON_6$ requires [M+H]$^+$ 327.19279]; LRMS *m/z* (ESI$^+$) 354 ([M+H]$^+$, 100%); HPLC retention time

1.5 min, 89.0%, retention time 1.9 min, 8.6% (thought to be two protonation states of compound as peaks are broad).

6'-Bromo-*N*-(4''-methoxyphenyl)-2'-(4-methyl-1*H*-imidazol-5-yl)imidazo[1,2-*a*]pyridin-3'-amine (3)

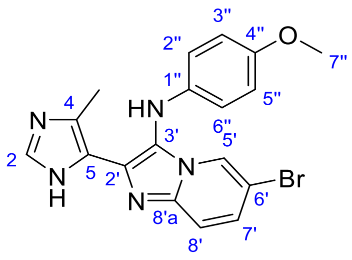

2-Amino-5-bromopyridine (100 mg, 0.578 mmol, 1.0 eq.) was dissolved in anhydrous dimethyl sulfoxide (1.60 mL). 4-Methoxyphenyl isocyanide (92.4 mg, 0.694 mmol, 1.2 eq.), 4-methyl-5-imidazolecarboxaldehyde (95.5 mg, 0.867 mmol, 1.5 eq.) and scandium triflate (28.4 mg, 0.0878 mmol, 0.1 eq.) were added. The reaction mixture was heated to 100 °C and stirred for 16.5 h. After this time TLC analysis indicated complete consumption of starting material (1:9 MeOH/CH_2Cl_2). The reaction was cooled to rt and quenched with H_2O (20 mL). The reaction mixture was extracted with CH_2Cl_2 (3 × 20 mL). The organic fractions were combined, washed sequentially with a saturated aqueous solution of $NaHCO_3$ (30 mL), and brine (30 mL), dried (Na_2SO_4), filtered, and concentrated *in vacuo*. The product was purified using silica flash column chromatography (elution with 0−10% MeOH/CH_2Cl_2 gradient) to yield the title compound as a yellow oil (138 mg, 60%): R_f 0.45 (1:2:12 NH_4OH/EtOH/EtOAc); $\bar{\nu}_{max}$/cm^{-1} 2537 (br), 2160 (s), 2033 (s), 1977 (s), 1602 (w), 1509 (m), 1460 (w), 1408 (w), 1325 (w), 1281 (w), 1246 (m), 1179 (w), 1128 (w), 1038 (w), 948 (w); ^{1}H NMR (500 MHz, CD_3OD) δ 7.96 (1H, dd, *J* 2.0, 0.9, H-5'), 7.60 (1H, s, H-2), 7.44 (1H, d, *J* 9.5, H-8'), 7.30 (1H, dd, *J* 9.5, 2.0, H-7'), 6.71 – 6.64 (2H, m, H-2'', H-6''), 6.46 – 6.39 (2H, m, H-3'', H-5''), 3.63 (3H, s, H-7''), 2.34 (3H, s, H-6); ^{13}C NMR (126 MHz, CD_3OD) δ 154.8 (H-4''), 141.9 (H-8'a), 139.6 (H-1''), 135.7 (H-2), 134.0 (H-5), 131.2 (H-4), 129.4 (H-7'), 126.0 (H-3'), 124.4 (H-2'), 122.4 (H-5'), 118.4 (H-8'), 116.0 (H-2'', H-6''), 115.8 (H-3'', H-5''), 108.0 (H-6'), 56.0 (H-7''), 12.0 (H-6); HRMS *m/z* (ESI$^+$) [Found: 398.06078, $C_{18}H_{17}{}^{79}BrN_5O$ requires [M+H]$^+$ 398.06110; Found: 400.05859, $C_{18}H_{17}{}^{81}BrN_5O$

requires $[M+H]^+$ 400.05905]; LRMS *m/z* (ESI^+) 306.2 ($[M+H]^+$, 100%); HPLC retention time 5.9 min, 95.6%.

***N*-Cyclohexyl-2'-(4-methyl-1*H*-imidazol-5-yl)imidazo[1,2-*a*]pyridin-3'-amine (4)**

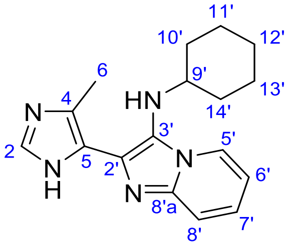

2-Aminopyridine (0.10 g, 1.1 mmol, 1.0 eq.) was dissolved in anhydrous dimethyl sulfoxide (3.0 mL). Cyclohexyl isocyanide (0.16 mL, 1.3 mmol, 1.2 eq.), 4-methyl-5-imidazole carboxaldehyde (0.18 g, 1.6 mmol, 1.5 eq.) and scandium triflate (52 mg, 0.11 mmol, 0.1 eq.) were added. The reaction mixture was heated to 100 °C and stirred for 17 h. After this time TLC analysis indicated complete consumption of starting material (1:9 MeOH/CH_2Cl_2). The reaction was cooled to rt and quenched with H_2O (20 mL). The reaction mixture was extracted with CH_2Cl_2 (3 × 20 mL). The organic fractions were combined, washed sequentially with a saturated aqueous solution of $NaHCO_3$ (30 mL), and brine (30 ml), dried (Na_2SO_4), filtered, and concentrated *in vacuo*. The product was purified using silica flash column chromatography (elution with 1:6 EtOH/EtOAc isocratic then 1:2:12 NH_4OH/EtOH/EtOAc isocratic) to yield the title compound as a yellow oil (98 mg, 31%): R_f 0.63 (1:2:12 NH_4OH/EtOH/EtOAc); $\bar{\nu}_{max}/cm^{-1}$ 2981 (br), 2462 (br), 2160 (s), 2030 (s), 1978 (s), 1694 (w), 1613 (w), 1541 (w), 1445 (w), 1339 (w), 1238 (w), 1164 (w), 1053 (m), 1026 (m); ^1H NMR (500 MHz, $CDCl_3$) δ 8.02 (1H, dd, *J* 6.8, 1.3, H-5'), 7.56 (1H, s, H-2), 7.47 (1H, dd, *J* 9.0, 1.1, H-8'), 7.07 (1H, ddd, *J* 9.0, 6.8, 1.3, H-7'), 6.76 (1H, ddd, *J* 6.8, 6.8, 1.1, H-6'), 2.95 – 2.85 (1H, m, H-9'), 2.60 (3H, s, H-6), 1.85 – 1.78 (2H, m, H-10'[a], H-14'[a]), 1.72 – 1.63 (2H, m, H-11'[a], H-13'[a]), 1.58 – 1.51 (1H, m, H-12'[a]), 1.28 – 1.10 (5H, m, H-10'[b], H-11'[b], H-12'[b], H-13'[b], H-14'[b]); ^{13}C NMR (126 MHz, $CDCl_3$) δ 141.3 (C-3'), 133.2 (C-2), 130.1 (C-4), 127.9 (C-2'), 127.4 (C-5), 126.2 (C-8'a), 123.3 (C-7'), 122.7 (C-5'), 116.9 (C-8'), 111.6 (C-6'), 56.4 (C-9'), 34.0 (C-10', C-14'), 25.9 (C-12'), 25.1 (C-11', C-13'), 11.9 (C-6); HRMS *m/z* (ESI^+) [Found:

296.18649, $C_{17}H_{22}N_5$ requires $[M+H]^+$ 296.18697]; LRMS *m/z* (ESI^+) 296.2 ($[M+H]^+$, 100%); HPLC retention time 5.5 min, 99.7%.

***N-tert*-Butyl-2'-(1*H*-pyrrolo[2,3-*b*]pyridin-3-yl)imidazo[1,2-*a*]pyridin-3'-amine (5)**

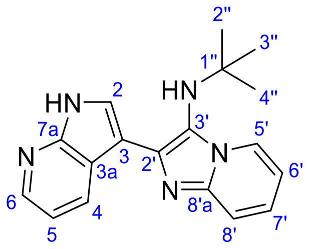

2-Aminopyridine (100 mg, 1.06 mmol, 1.5 eq.) was dissolved in anhydrous dimethyl sulfoxide (3.00 mL). *tert*-Butyl isocyanide (80.0 µL, 0.706 mmol, 1.0 eq.), 7-azaindole-3-carboxaldehyde (232 mg, 1.59 mmol, 2.3 eq.) and scandium triflate (52.2 mg, 0.106 mmol, 0.2 eq.) were added. The reaction mixture was heated to 100 °C and stirred for 17 h. After this time TLC analysis indicated complete consumption of starting material (1:9 MeOH/CH_2Cl_2). The reaction was cooled to rt and quenched with H_2O (20 mL). The reaction mixture was extracted with CH_2Cl_2 (3 × 20 mL). The organic fractions were combined, washed sequentially with a saturated aqueous solution of $NaHCO_3$ (30 mL), and brine (30 mL), dried (Na_2SO_4), filtered, and concentrated *in vacuo*. The product was purified using silica flash column chromatography (elution with 0−9% MeOH/CH_2Cl_2 gradient) to yield the title compound as a pale green solid (102 mg, 47%): R_f 0.52 (1:2:12 NH_4OH/EtOH/EtOAc); $\bar{\nu}_{max}/cm^{-1}$ 3068 (br), 2963 (br), 2568 (br), 2160 (m), 2031 (m), 1978 (m), 1629 (w), 1574 (w), 1527 (w), 1501 (w), 1453 (w), 1423 (w), 1390 (w), 1363 (w), 1339 (w), 1320 (w), 1284 (w), 1269 (w), 1221 (w), 1191 (w), 1141 (w), 1120 (w), 1015 (w); ^1H NMR (500 MHz, $CDCl_3$) δ 10.14 (1H, br s, N-H), 8.55 (1H, d, *J* 7.9, H-4), 8.38 (1H, d, *J* 4.3, H-6), 8.23 (1H, d, *J* 6.8, H-5'), 7.84 (1H, s, H-2), 7.57 (1H, d, *J* 9.1, H-8'), 7.19 (1H, dd, *J* 7.9, 4.3, H-5), 7.14 (1H, ddd, *J* 9.1, 6.8, 1.3, H-7'), 6.79 (1H, ddd, *J* 6.8, 6.8, 1.1, H-6'), 3.06 (1H, br s, N-H), 1.05 (9H, s, H-2'', H-3'', H-4''); ^{13}C NMR (126 MHz, $CDCl_3$) δ 148.8 (C-7a), 143.4 (C-6), 142.4 (C-8'a), 135.4 (C-3'), 130.4 (C-4), 123.92 (C-7'), 123.85 (C-2), 123.3 (C-5'), 123.1 (C-2'), 119.4 (C-3a), 117.1 (C-8'), 116.5 (C-5), 111.5 (C-6'), 110.1 (C-3), 56.6 (C-1''), 30.5 (C-2'', C-3'', C-4'');

HRMS *m/z* (ESI^+) [Found: 306.17127, $C_{18}H_{20}N_5$ requires $[M+H]^+$ 306.17132]; LRMS *m/z* (ESI^+) 306.2 ($[M+H]^+$, 100%); HPLC retention time 5.6 min, 99.8%.

Methyl 3-(cyclohexylamino)-2-(2’’-methyl-1*H*-imidazol-4’’-yl)imidazo[1,2-a]pyridine-6-carboxylate (6)

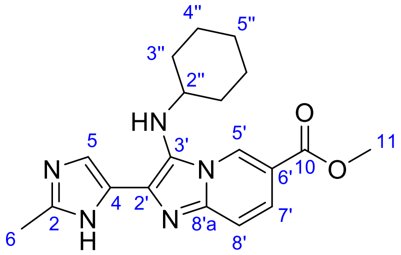

Methyl 6-aminopyridine-3-carboxylate (100 mg, 0.657 mmol, 1.0 eq.) was dissolved in anhydrous dimethyl sulfoxide (3.00 mL). 2-Methyl-1*H*-imidazole-4-carbaldehyde (109 mg, 0.986 mmol, 1.5 eq.), cyclohexyl isocyanide (94.7 µL, 0.762 mmol, 1.16 eq.) and scandium(III) triflate (25.9 mg, 0.0526 mmol, 0.08 eq.) were added. The reaction mixture was heated to 100 °C and stirred for 17 h. After this time TLC analysis indicated complete consumption of starting material (1:9 $MeOH/CH_2Cl_2$). The reaction was cooled to rt and quenched with H_2O (20 mL). The reaction mixture was extracted with CH_2Cl_2 (3 × 20 mL). The organic fractions were combined, washed sequentially with a saturated aqueous solution of $NaHCO_3$ (30 mL), and brine (30 mL), dried (Na_2SO_4), filtered, and concentrated *in vacuo*. The product was purified using silica flash column chromatography (elution with 10% EtOH/EtOAc, followed by 1:2:12 NH_4OH/EtOH/EtOAc isocratic) and further purified using silica flash column chromatography (elution with 2–6% $MeOH/CH_2Cl_2$ gradient) to yield the title compound as a yellow oil (23.1 mg, 10%): R_f 0.03 (8% $MeOH/CH_2Cl_2$); $\bar{v}_{max}$/cm^{-1} 3148 (w), 2928 (m), 2853 (w), 2343 (w), 1724 (m, C=O), 1630 (w), 1556 (w), 1437 (w), 1413 (w), 1339 (w), 1290 (s), 1194 (w), 1127 (w), 1062 (w), 1002 (w), 907 (w), 817 (w), 764 (m), 732 (m); ^{1}H NMR (500 MHz, $CDCl_3$) δ 8.81 (1H, dd, *J* 1.8, 1.0, H-5’), 7.63 (1H, dd, *J* 9.4, 1.8, H-7’), 7.40 (1H, d, *J* 9.4, H-8’), 7.37 (1H, s, H-5), 3.96 (3H, s, H-11), 3.07 (1H, tt, *J* 10.4, 3.8, H-2’’), 2.41 (3H, s, H-6), 1.91 – 1.83 (2H, m, H-3’’^{a}), 1.78 – 1.68 (2H, m, H-4’’^{a}), 1.63 – 1.54 (1H, m, H-5’’^{a}), 1.40 – 1.28 (2H, m, H-3’’^{b}), 1.28 – 1.15 (3H, m, H-4’’^{b}, H-5’’^{b}); ^{13}C NMR (126 MHz, $CDCl_3$) δ 165.9 (C-10), 144.8 (C-2), 142.0 (C-8’a), 131.0 (C-4, C-2’),

127.0 (C-5'), 126.2 (C-3'), 123.3 (C-7'), 118.7 (C-5), 115.9 (C-8'), 115.7 (C-6'), 56.7 (C-2''), 52.5 (C-11), 34.2 (C-3''), 25.8 (C-5''), 25.0 (C-4''), 14.3 (C-6); HRMS *m/z* (ESI^+) [Found: 354.19223, $C_{19}H_{24}O_2N_5$ requires $[M+H]^+$ 354.19245]; LRMS *m/z* (ESI^+) 354 ($[M+H]^+$, 100%); HPLC retention time 5.9 min, 97.8%.

Methyl 1,3-dimethyl-2-oxo-2,3-dihydro-1*H*-benzimidazole-5-carboxylate

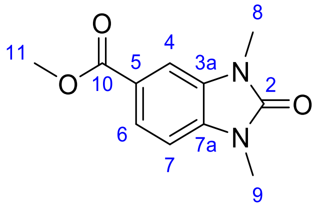

3,4-Diaminobenzoic acid (5.00 g, 32.9 mmol, 1.0 eq.) was suspended in anhydrous CH_2Cl_2 (40.0 mL) and 1,1'-carbonyldiimidazole (11.6 g, 71.5 mmol, 2.2 eq.) was added. The reaction mixture was stirred at rt for 1 h. After this time TLC analysis indicated complete consumption of starting material (3:7 EtOH/CH_2Cl_2). MeOH (30.0 mL) was added, and the reaction mixture stirred at rt for 2 h. After this time TLC analysis indicated complete consumption of the intermediate (1:9 MeOH/CH_2Cl_2). The reaction mixture was cooled on ice and the precipitate collected *via* filtration. The filter pad was washed with H_2O. The precipitate was suspended in MeOH (30.0 mL) and the solvent removed *in vacuo*. The solid was suspended in *N,N*-dimethylformamide (60.0 mL) and K_2CO_3 (18.1 g, 132 mmol, 4.0 eq.) was added. The reaction mixture was cooled to 0 °C and methyl iodide (28.7 mL, 461 mmol, 14.0 eq.) was added dropwise. The reaction mixture was warmed to rt and stirred for 17 h. After this time TLC analysis indicated complete consumption of the intermediate (1:9 MeOH/CH_2Cl_2). The reaction was quenched with H_2O (50 mL) and extracted with EtOAc (50 mL). The organic fraction was washed sequentially with an aqueous solution of LiCl (0.5 M, 4 × 50 mL), and brine (50 mL). The organic fraction was dried ($MgSO_4$), filtered, and concentrated *in vacuo* to yield the title compound as a light brown solid (4.69 g, 21.3 mmol, 65%): R_f 0.24 (2:3 petroleum ether/EtOAc); m.p. 145–147 °C (from EtOAc); $\bar{\nu}_{max}/cm^{-1}$ 3063 (br), 2917 (br), 1698 (s), 1622 (m), 1509 (m), 1463 (m), 1445 (m), 1225 (s), 1103 (s), 977 (m); ^1H NMR (400 MHz, $CDCl_3$) δ 7.87 (1H, dd, *J* 8.2, 1.6, H-6), 7.66 (1H, d, *J* 1.6, H-4), 6.99 (1H, d, *J* 8.2, H-7), 3.93 (3H, s, H-11), 3.46 (3H, s, H-8), 3.45 (3H, s, H-9); ^{13}C NMR (101 MHz,

$CDCl_3$) δ 167.2 (C-10), 154.9 (C-2), 133.8 (C-3a), 129.9 (C-7a), 124.0 (C-6), 123.3 (C-5), 108.6 (C-4), 106.7 (C-7), 52.2 (C-2), 27.41 (C-8/9), 27.35 (C-8/9); HRMS *m/z* (CI^+) [Found: 221.0920, $C_{11}H_{12}N_2O_3$ requires $[M+H]^+$ 221.0921]; not detected by LRMS; HPLC retention time 7.3 min, 98.2%.

5-(Hydroxymethyl)-1,3-dimethyl-1,3-dihydro-2*H*-benzimidazol-2-one

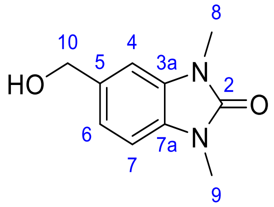

Method 1

Methyl 1,3-dimethyl-2-oxo-2,3-dihydro-1*H*-benzimidazole-5-carboxylate (473 mg, 2.15 mmol, 1.0 eq.) was dissolved in anhydrous tetrahydrofuran (10.0 mL). Lithium borohydride (2 M, THF) (2.15 mL, 4.30 mmol, 2.0 eq.) and MeOH (250 µL) were added dropwise. The reaction mixture was heated to 80 °C and stirred for 19 h. After this time TLC analysis indicated complete consumption of starting material (2:3 EtOAc/petroleum ether). The reaction was cooled to rt, quenched with a saturated aqueous solution of $NaHCO_3$ (20 mL) and extracted with CH_2Cl_2 (2 × 20 mL). The organic fractions were combined and washed sequentially with H_2O (20 mL), and brine (20 mL). The organic fraction was dried ($MgSO_4$), filtered, and concentrated *in vacuo* to yield the title compound as a light brown solid (325 mg, 1.69 mmol, 79%): R_f 0.65 (4:1 CH_2Cl_2/EtOH); m.p. 142–144 °C (from EtOAc); $\bar{\nu}_{max}$/cm^{-1} 3368 (br), 2862 (br), 1675 (s), 1508 (m), 1459 (m), 1398 (m), 1272 (w), 1194 (w), 1139 (w), 1027 (m), 988 (m); ^{1}H NMR (400 MHz, $CDCl_3$) δ 7.06 (1H, dd, *J* 7.9, 1.5, H-6), 6.97 (1H, d, *J* 1.5, H-4), 6.86 (1H, d, *J* 7.9, H-7), 4.70 (2H, s, H-10), 3.35 (3H, s, H-8), 3.34 (3H, s, H-9), 1.85 (1H, br s, O-H); ^{13}C NMR (126 MHz, $CDCl_3$) δ 154.9 (C-2), 134.8 (C-5), 130.1 (C-3a), 129.5 (C-7a), 120.4 (C-6), 107.1 (C-7), 106.5 (C-4), 65.6 (C-10), 27.32 (C-8/9), 27.27 (C-8/9); HRMS *m/z* (ESI^+) [Found: 193.09728, $C_{10}H_{12}N_2O_2$ requires $[M+H]^+$ 193.09715]; LRMS *m/z* (ESI^+) 193.0 ($[M+H]^+$, 100%); HPLC retention time 5.4 min, 98.9%.

Method 2

Methyl 1,3-dimethyl-2-oxo-2,3-dihydro-1*H*-benzimidazole-5-carboxylate (1.00 g, 4.54 mmol, 1.0 eq.) was dissolved in anhydrous toluene (40.0 mL) and cooled to –78 °C. Diisobutylaluminium hydride (1 M, hexanes) (45.4 mL, 45.4 mmol, 10 eq.) was added dropwise and the reaction mixture stirred at –78 °C for 5.5 h. After this time TLC analysis indicated complete consumption of starting material (3:2 EtOAc/petroleum ether). The reaction was quenched at –78 °C with aqueous HCl (1 M, 30 mL) and warmed to rt. The reaction mixture was diluted with H_2O (50 mL) and extracted with EtOAc (3 × 50 mL). The organic fractions were combined, dried ($MgSO_4$), filtered, and concentrated *in vacuo* to yield the title compound as a light brown solid (780 mg, 4.06 mmol, 89%). The spectroscopic data are identical to those obtained using Method 1, above.

1,3-Dimethyl-2-oxo-2,3-dihydro-1*H*-benzimidazole-5-carbaldehyde

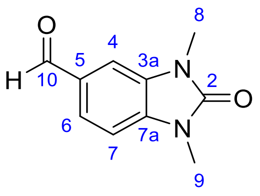

Method 1

5-(Hydroxymethyl)-1,3-dimethyl-1,3-dihydro-2*H*-benzimidazol-2-one (20 mg, 0.10 mmol, 1.0 eq.) was dissolved in tetrahydrofuran (0.80 mL) and Dess-Martin periodinane (88 mg, 0.21 mmol, 2.0 eq.) was added. The reaction mixture was stirred at rt for 3 h. After this time TLC analysis indicated complete consumption of starting material (1:9 MeOH/CH_2Cl_2). The reaction was quenched with an aqueous solution of Na_2SO_3 (2 M, 10 mL) and extracted with EtOAc (10 mL). The organic fraction was washed sequentially with a saturated aqueous solution of $NaHCO_3$ (10 mL), and brine (10 mL). The organic fraction was dried ($MgSO_4$), filtered, and concentrated *in vacuo*. The product was purified using silica flash column chromatography (elution with 0–10% EtOAc/petroleum ether gradient) to yield the title compound as a colourless solid (13 mg, 0.066 mmol, 63%): R_f 0.37 (19:1 CH_2Cl_2/EtOH); m.p. 142–144 °C (from CH_2Cl_2); $\bar{\nu}_{max}$/cm^{-1} 3044 (br), 2941 (br), 1712 (s), 1670 (s), 1620 (m), 1600 (m), 1509 (m), 1481 (s), 1448 (s), 1397 (m), 1353 (m), 1270 (m), 1190 (s), 914 (w); ^{1}H NMR (400 MHz, $CDCl_3$)

δ 9.95 (1H, s, H-10), 7.65 (1H, dd, *J* 8.0, 1.5, H-6), 7.54 (1H, d, *J* 1.5, H-4), 7.09 (1H, d, *J* 8.0, H-7), 3.48 (6H, s, H-8 and H-9); ^{13}C NMR (126 MHz, $CDCl_3$) δ 191.3 (C-10), 154.7 (C-2), 135.3 (C-7a), 130.7 (C-5), 130.6 (C-3a), 126.7 (C-6), 107.0 (C-4), 106.4 (C-7), 27.5 (C-8/9), 27.4 (C-8/9); HRMS *m/z* (EI^+) [Found: 190.0731, $C_{10}H_{10}N_2O_2$ requires $[M]^+$ 190.0737]; unable to detect by LRMS; HPLC retention time 6.3 min, 99.2%.

Method 2

5-(Hydroxymethyl)-1,3-dimethyl-1,3-dihydro-2*H*-benzimidazol-2-one (942 mg, 4.90 mmol, 1.0 eq.) was suspended in CH_2Cl_2 (100 mL) and MnO_2 (12.8 g, 147 mmol, 30.0 eq.) was added. The reaction mixture was stirred at rt for 2 h. After this time TLC analysis indicated complete consumption of starting material (1:9 MeOH/CH_2Cl_2). The reaction mixture was filtered through a pad of Celite® and the filtrate concentrated *in vacuo* to yield the title compound as a colourless solid (763 mg, 3.99 mmol, 81%). The spectroscopic data are identical to those obtained using Method 1, above.

4'-(2''-{[(1,3-Dimethyl-2-oxo-2,3-dihydro-1*H*-benzimidazol-5-yl)methyl]amino}ethyl)benzene-1'-sulfonamide (8)

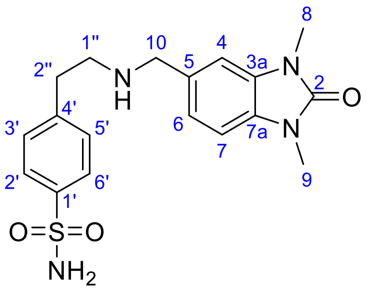

1,3-Dimethyl-2-oxo-2,3-dihydro-1*H*-benzimidazole-5-carbaldehyde (100 mg, 0.526 mmol, 1.0 eq.) was dissolved in anhydrous 1,2-dichloroethane (13.0 mL) and the reaction mixture cooled to 0 °C. 4-(2-Aminoethyl)benzenesulfonamide (211 mg, 1.05 mmol, 2.0 eq.) and acetic acid (60.0 µL, 1.05 mmol, 2.0 eq.) were added and the reaction mixture stirred at 0 °C for 6 h. After this time TLC analysis indicated partial imine formation (1:9 MeOH/CH_2Cl_2). $NaBH(OAc)_3$ (557 mg, 2.63 mmol, 5.0 eq.) was added at 0 °C. The reaction mixture was warmed to rt and stirred for 15 h. The reaction was quenched with a saturated aqueous solution of $NaHCO_3$ (15 mL) and extracted with CH_2Cl_2 (2 ×

15 mL). The aqueous fractions were extracted with EtOAc (15 mL). The organic fractions were combined, dried ($MgSO_4$), filtered, and concentrated *in vacuo*. The product was purified using silica flash column chromatography (elution with 100% EtOH isocratic) to yield the title compound as a colourless solid (50.0 mg, 0.134 mmol, 25%): R_f 0.12 (EtOH); m.p. >160 °C (from EtOH); $\bar{\nu}_{max}/cm^{-1}$ 3280 (N-H, br), 2852 (br), 1679 (s), 1513 (w), 1464 (w), 1334 (m), 1161 (S=O, s), 1121 (m), 1097 (m), 1019 (w), 990 (w); ^{1}H NMR (500 MHz, D_6-DMSO) δ 7.71 (2H, d, *J* 8.3, H-2', H-6'), 7.39 (2H, d, *J* 8.3, H-3', H-5'), 7.27 (2H, br s, NH_2), 7.09–7.02 (2H, m, H-4, H-7), 6.99 (1H, dd, *J* 7.9, 1.5, H-6), 3.73 (2H, s, H-10), 3.30 (3H, s, H-8), 3.29 (3H, s, H-9), 2.83–2.76 (2H, m, H-1''), 2.77–2.69 (2H, m, H-2''); ^{13}C NMR (126 MHz, D_6-DMSO) δ 154.0 (C-2), 145.0 (C-4'), 141.7 (C-1'), 133.9 (C-5), 129.6 (C-3a), 129.1 (C-3', C-5'), 128.4 (C-7a), 125.6 (C-2', C-6'), 120.6 (C-6), 107.2 (C-4), 107.1 (C-7), 53.0 (C-10), 49.8 (C-1''), 35.6 (C-2''), 26.9 (C-8, C-9); HRMS *m/z* (ESI^+) [Found: 375.14846, $C_{18}H_{22}N_4O_3S$ requires $[M+H]^+$ 375.14854]; LRMS *m/z* (ESI^-) 373.1 ($[M–H]^-$, 100%); not detected by LRMS; HPLC retention time 5.5 min, 97.7%.

Table S20. Dose-response AlphaScreen™ IC_{50} values of patent compounds **2–6** for all nine protein-peptide combinations. Compounds were serially diluted 1:2 from 500 µM highest compound concentration. PHD refers to peptide $H3_{1–27}K9Me_3$, BRD refers to peptide $H3_{1–27}K18Ac$, and DUAL refers to peptide $H3_{1–27}K9Me_3K18Ac$. The colour scale is shown below, with low IC_{50} values shown in red, and high IC_{50} values shown in green.

Compound		AlphaScreen IC_{50} (µM)								
		TRIM33β			TRIM33α			TRIM24		
		$K9Me_3K18Ac$	K18Ac	$K9Me_3$	$K9Me_3K18Ac$	K18Ac	$K9Me_3$	$K9Me_3K18Ac$	K18Ac	$K9Me_3$
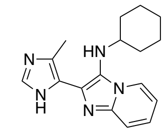	**5**	3.111 ± 0.342	3.158 ± 0.545	3.369 ± 0.619	1.680 ± 0.407	2.737 ± 0.529	2.712 ± 0.166	3.325 ± 0.226	4.206 ± 0.665	3.570 ± 0.521
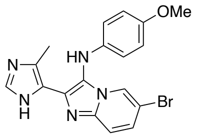	**3**	1.822 ± 0.206	1.491 ± 0.218	1.397 ± 0.194	1.146 ± 0.120	1.583 ± 0.165	1.890 ± 0.118	1.462 ± 0.084	1.763 ± 0.100	1.857 ± 0.182
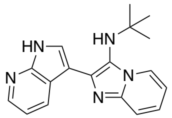	**7**	>250	>250	>250	>250	>250	>250	58.04 ± 20.35	43.76 ± 10.86	105.3 ± 26.71
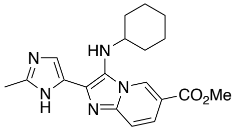	**6**	8.858 ± 2.409	7.908 ± 1.843	6.695 ± 1.794	75.14 ± 20.54	16.59 ± 6.104	>250	11.13 ± 1.811	19.95 ± 2.945	17.12 ± 6.153
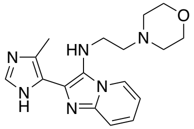	**4**	58.69 ± 5.996	55.28 ± 4.68	>250	>250	>250	>250	23.09 ± 0.740	18.61 ± 0.727	52.03 ± 5.561

Weaker binding 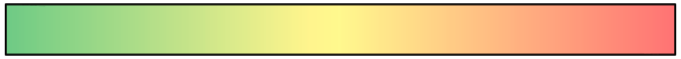 **Stronger binding**

Table S21. AlphaScreen™ TruHits of compounds **3–7**. Assay 1 requires incubation of compound at varying concentrations with streptavidin donor beads and biotinylated acceptor beads in the absence of protein and peptide. The signal difference from the DMSO average, normalised against the standard deviation of DMSO control, is calculated. Values greater than 2 (highlighted in pink) indicates significant assay interference. Assay 2 involves pre-incubation of compound at varying concentrations with streptavidin donor beads in the absence of protein and peptide. Acceptor beads are subsequently added. Biotin mimetic compounds will bind to the streptavidin donor beads and generate a signal loss. Again, value greater than 2 (highlighted in pink) indicates significant assay interference.

Compound		Difference from DMSO average, normalised against standard deviation of DMSO control									
		Assay 1: Compound concentration (µM) with pre-					Assay 2: Compound concentration (µM) with pre-				
		31.25	62.5	125	250	500	31.25	62.5	125	250	500
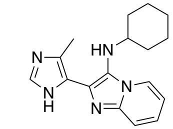	**5**	40.86631	42.33376	42.97065	43.37071	43.63590	3.844188	3.941630	4.025278	4.041796	4.061472
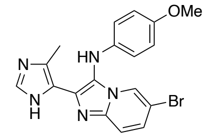	**3**	42.86535	43.34006	43.58664	43.68103	43.68017	3.997283	4.035840	4.059712	4.063040	4.064318
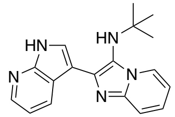	**7**	2.873695	8.918652	17.81176	25.27034	32.73463	0.791912	1.271922	1.653936	2.277407	3.225081
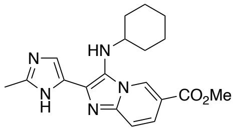	**6**	21.00550	19.76253	23.80389	29.86232	35.27239	2.089855	2.480513	3.087756	3.547185	3.836279
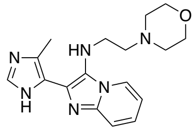	**4**	1.972622	2.577211	3.961041	2.641797	4.384560	2.073506	2.892511	3.158311	3.003649	3.661506

Isothermal Titration Calorimetry with TRIM33β

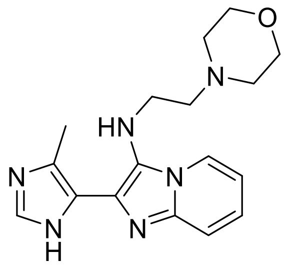

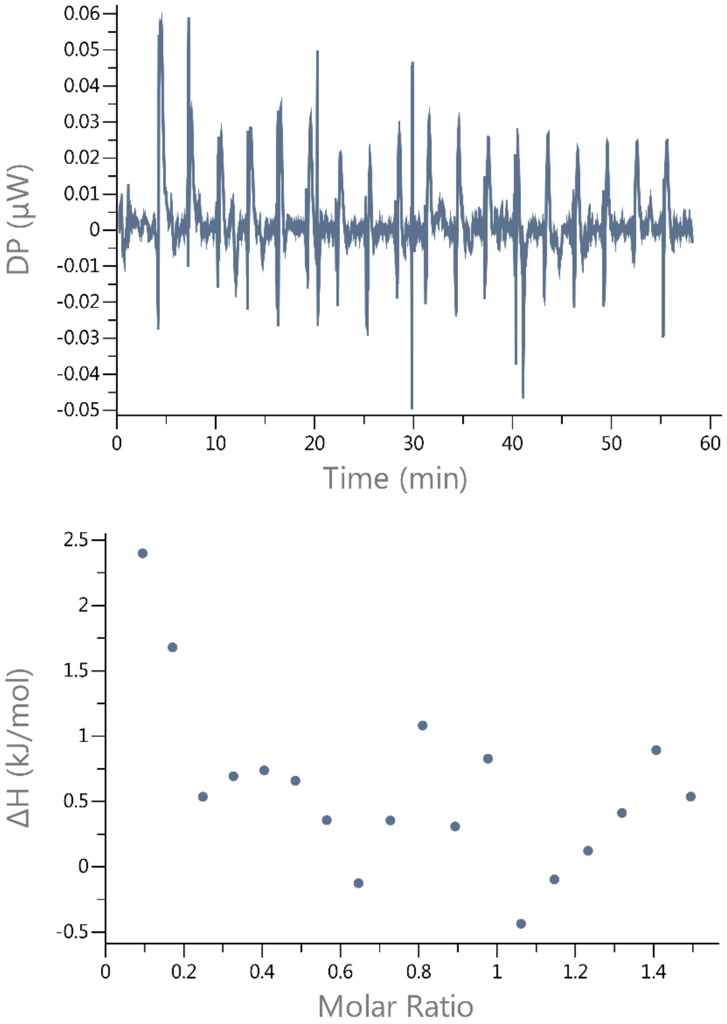

Figure S3. ITC trace of compound **4**, obtained using MicroCal PEAQ-ITC (Malvern). The cell contained **4** at a concentration of 40 μM, and the syringe contained TRIM33β at a concentration of 316 μM.

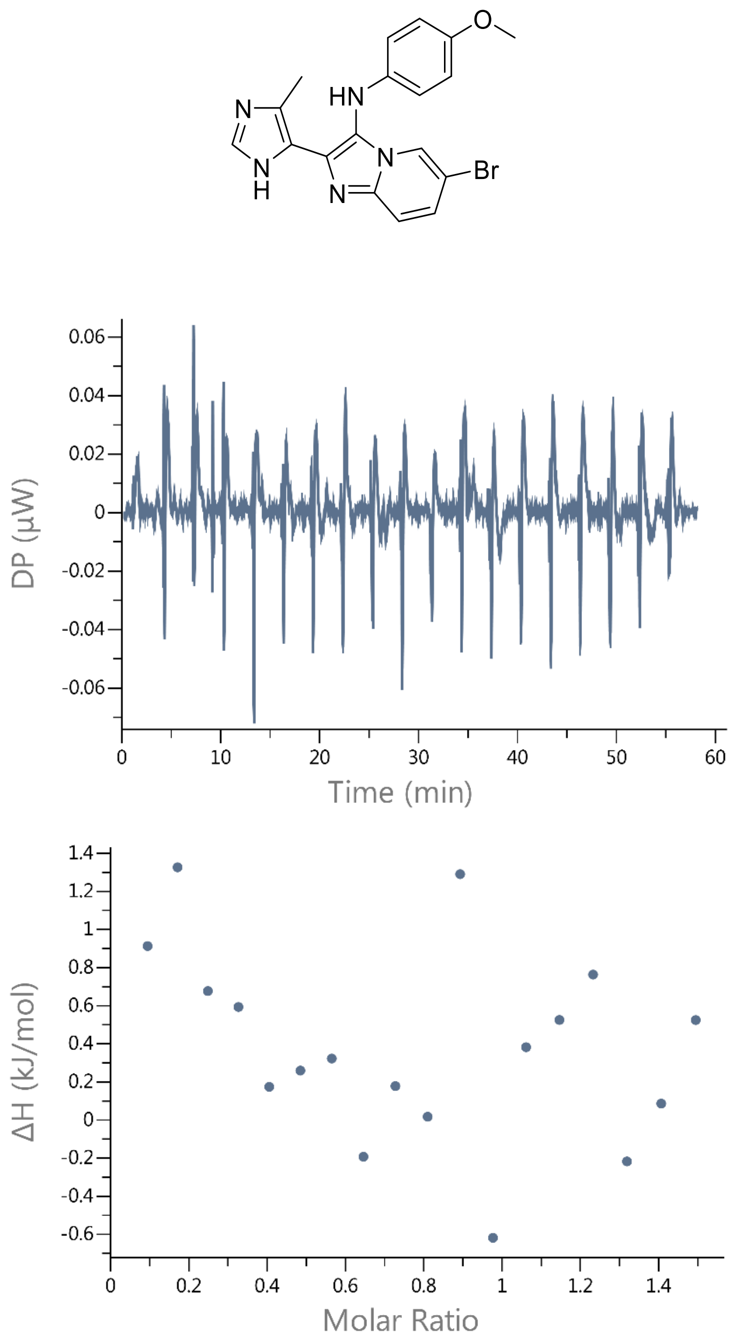

Figure S4. ITC trace of compound **3**, obtained using MicroCal PEAQ-ITC (Malvern). The cell contained **3** at a concentration of 40 μM, and the syringe contained TRIM33β at a concentration of 316 μM.

WaterLOGSY with TRIM33β.

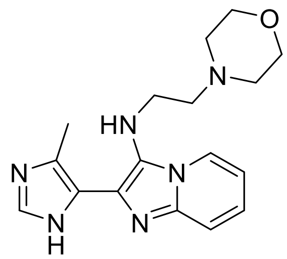

2

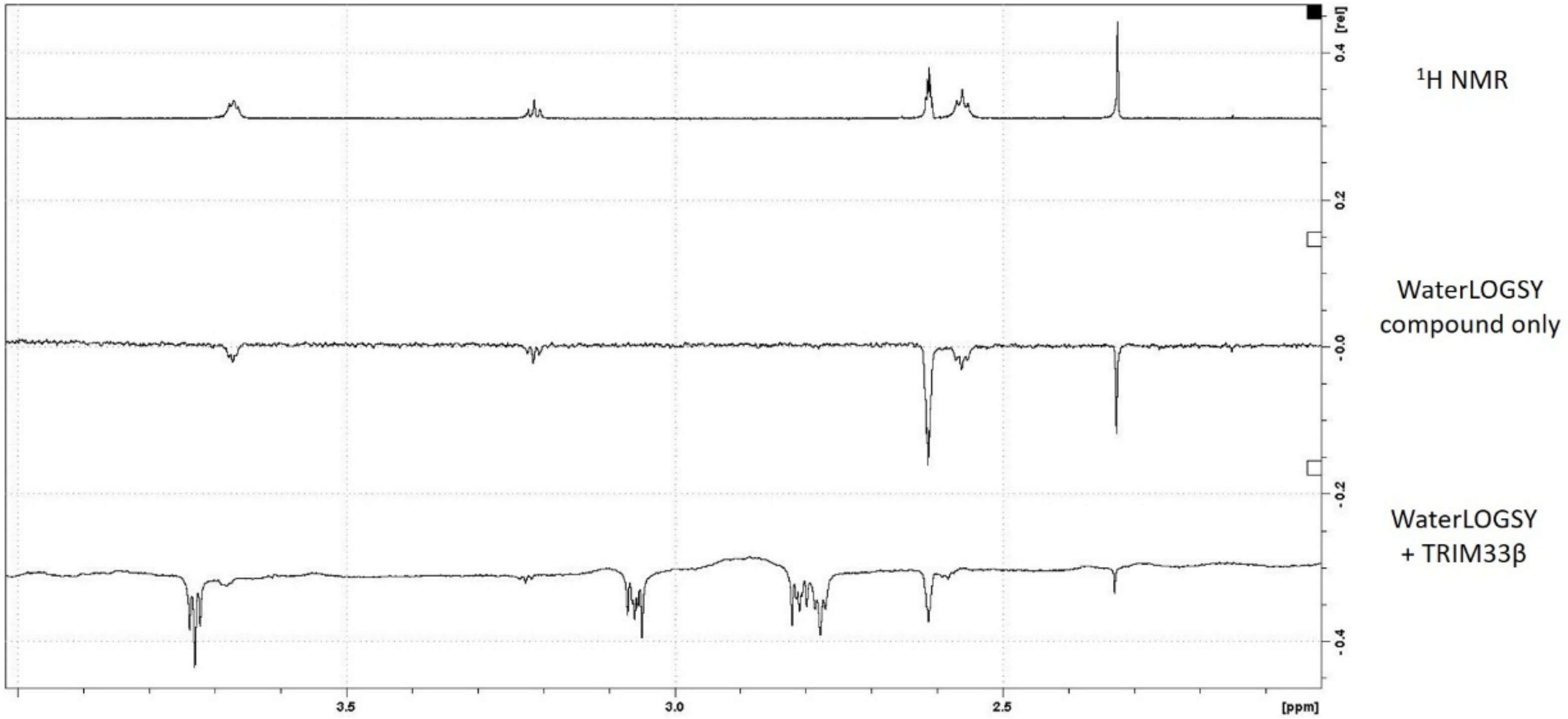

Figure S5. WaterLOGSY spectra for **2**. On addition of TRIM33β, the signal remained negative, indicating that **2** is not binding.

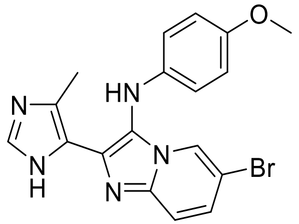

3

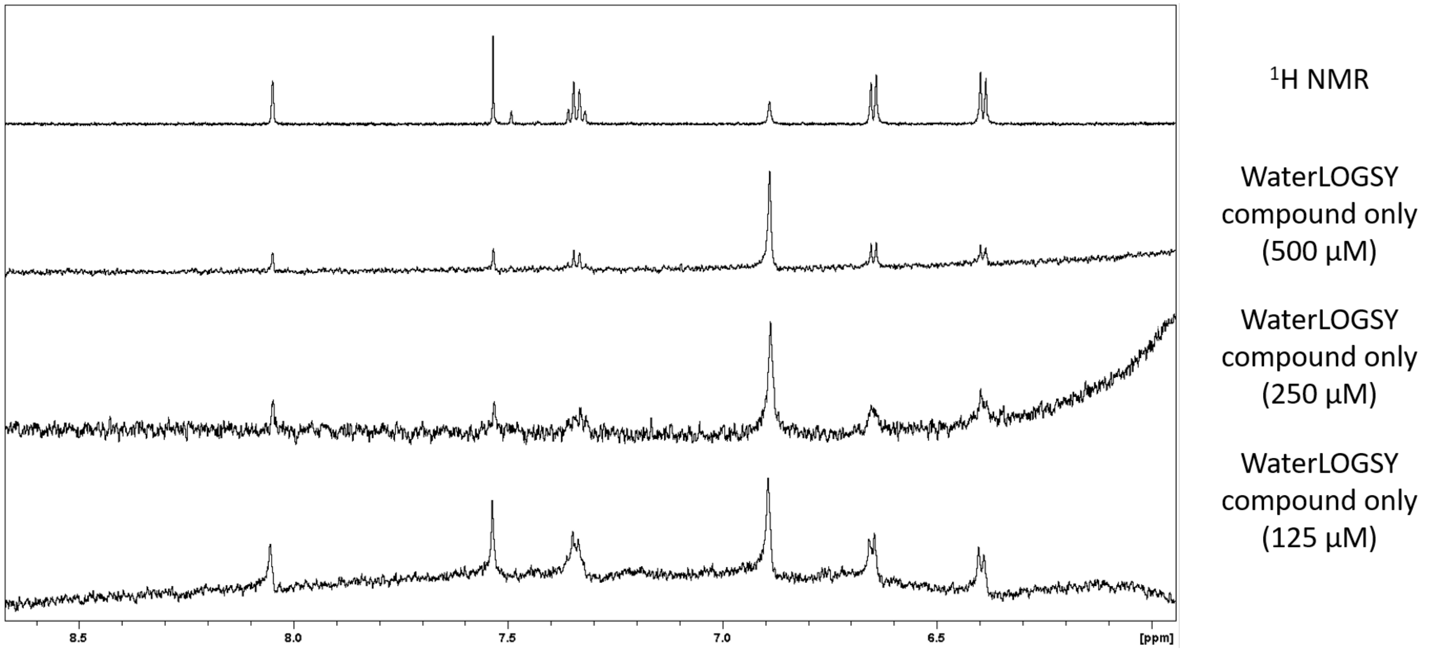

Figure S6. WaterLOGSY spectra for **3**. The signals were positive during the WaterLOGSY experiment without protein present, indicating aggregation was occurring. Despite multiple compound dilutions, aggregation was still occurring, indicating this assay is not suitable to determine binding to TRIM33.

Figure S7. Dose response AlphaScreen for X1 and X2

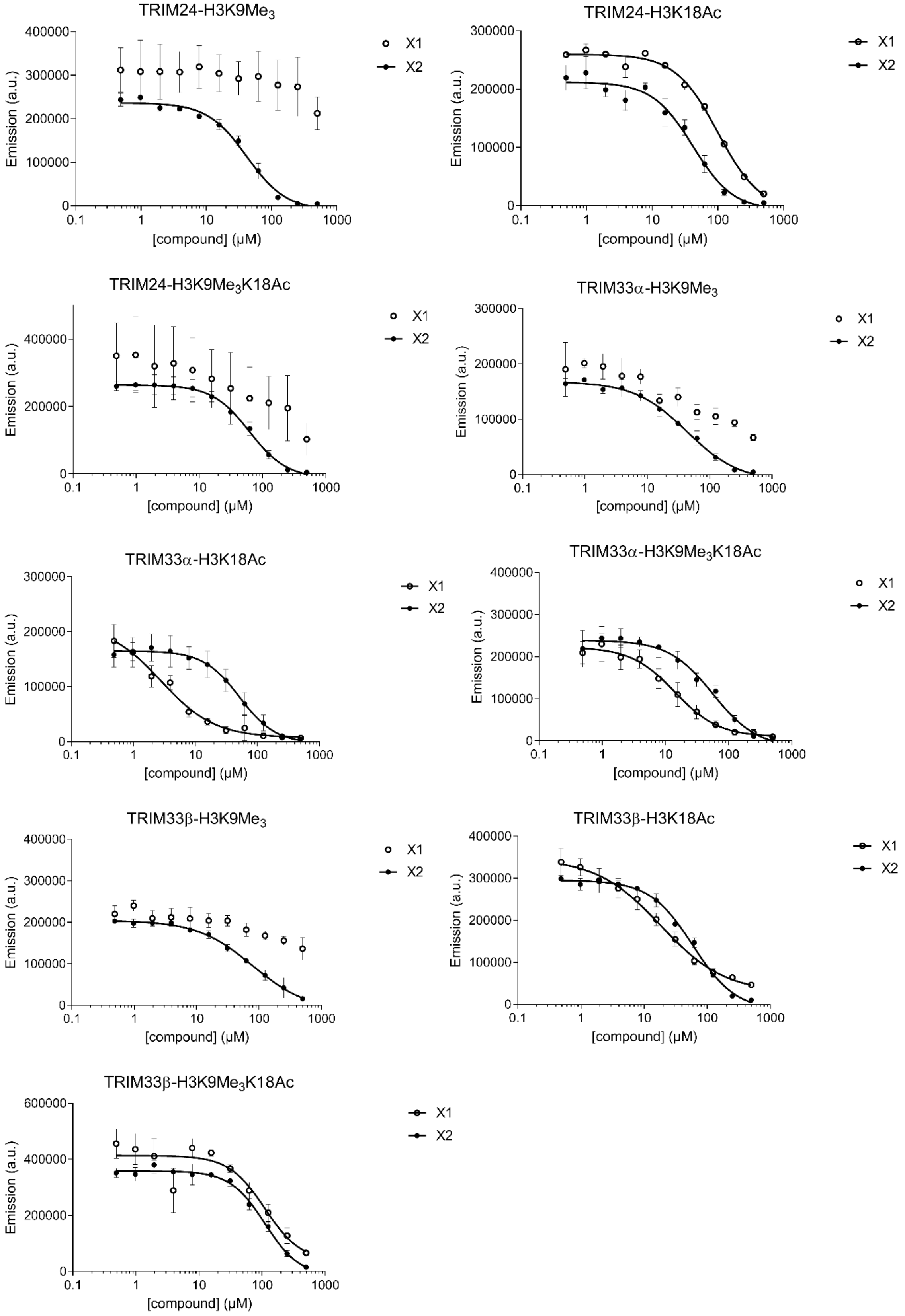

Figure S8A. AlphaScreen dose-response curves for compound **8** and positive control OXFBD02 binding to BRD4(1) in combination with $H4_{1\text{–}20}(KAc)_4$. Compounds were serially diluted 1:2 from 500 µM highest compound concentration. Experimental details as described in Hewings *et al.*[6] and Philpott *et al.*[7] OXFBD02 has an IC_{50} value of 485 nM, which is in line with the literature values. Compound **8** shows only weak binding to BRD4(1) and does not plateau. **B**. The chemical structures of OXFBD02 and **8**.

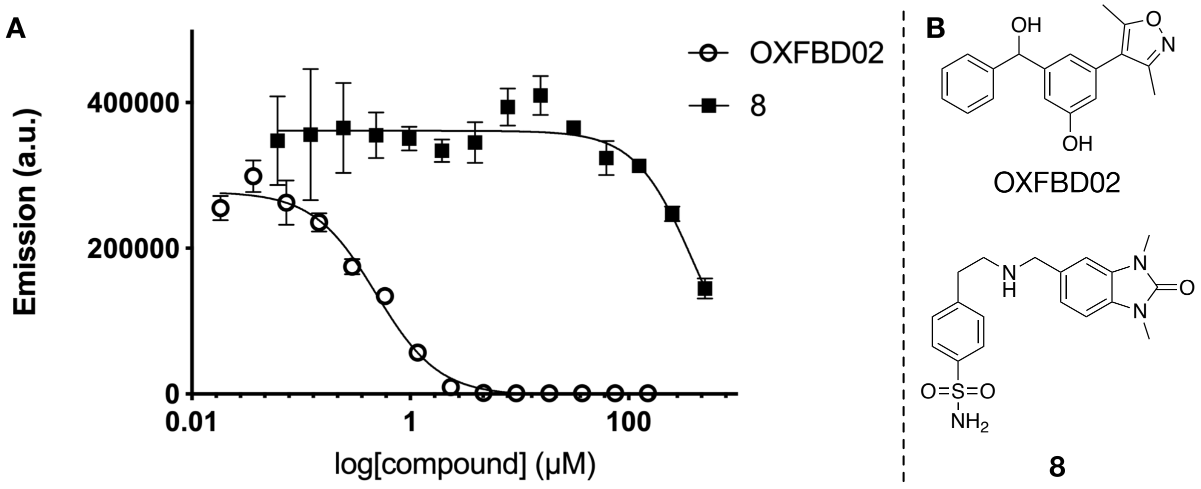

Figure S9. Circular Dichroism spectra from TRIM24 (left) and TRIM33 (right) cleaved from various constructs, in comparison to polyhistidine labelled protein (His_6-). Smoothed average of results in triplicate with 0.2 nm step size, 0.5 sec/step, 10 mm path length, 0.2 mg/mL protein in NaF buffered phosphate buffer (pH 7.8). –His_6: TEV cleavage from pNIC28-Bsa4. –GST: TEV cleavage from pGTvL1-SGC. –SUMO: SENP1 cleavage from pETM11-SUMO3.

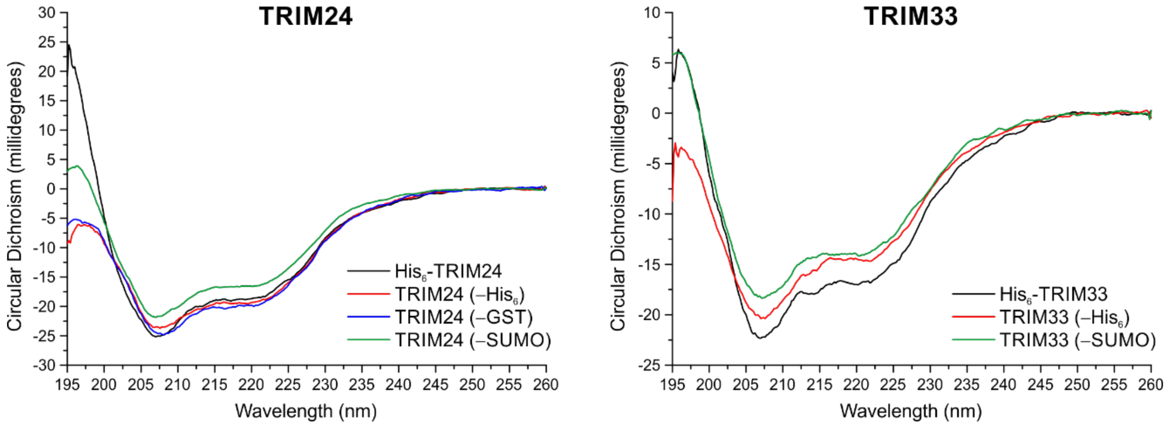

3 Computational Methods

3.1 Virtual Screening Workflow

3.1.1 File Preparation

Receptor Files

The TRIM24 receptor file was prepared from chain A of the *apo*-crystal structure of the protein (PDB: 3O33). Water molecules were assessed for likelihood of displacement using WaterClassifier,[8] with scores appended to PDB files as a new property (in place of the B-factor) for graphical representation in PyMOL. Water molecules located in the base of the KAc pocket were manually corrected to account for the hydrogen bonding network, before those with a displaceability of >80 % were removed using the built in `extract` function.

The TRIM33α receptor file was prepared from chain A of the *apo*-crystal structure of the protein (PDB: 3U5M). Water molecule positions were not resolved in this crystal structure of TRIM33α due to low resolution, and were modelled using WaterDock2.1.[8] Reliability of this method, particularly for hydrogen bonded networks, was not validated, so comparable water positions were overlaid from the TRIM24 receptor hydration state. Water positions for each domain – PHD finger and bromodomain – were aligned separately in PyMOL using the `cealign` function.

Protonation states were set for pH 7.0 using the AMBER force field in PROPKA's online server (http://nbcr.net/pdb2pqr), including optimisation of hydrogen bonding and steric restrictions on proximity of atoms. Charges were merged for non-polar hydrogens and lone pairs using the AutoDockTools script `prepare_receptor4.py`.[9]

Ligand Files

The Maybridge Fragment Collection, provided by Fisher Scientific, contained 31,973 molecules. Charges were merged for non-polar hydrogens and lone pairs using the AutoDockTools script `prepare_ligand4.py`.

3.1.2 Docking

Docking was performed using AutoDock Vina.[10] Simulations generated 20 modes per computation and the lowest energy conformation was retained.

3.1.3 Results Analysis

The top 1000 results from each screen were extracted, and the predicted affinity data were extracted from docking results, and the distributions visualised as histograms. Energetic cut-offs were selected to reduce the number to a sum suitable for manual curation. Reduction criteria and resultant numbers of molecules were: TRIM24 KAc, –7.3 kcal/mol, 381 molecules; TRIM24 cleft, –6.4 kcal/mol, 430 molecules; TRIM33α KAc, –7.5 kcal/mol, 435 molecules; TRIM33α cleft, –6.6 kcal/mol, 294 molecules.

The collection of all identified compounds was combined (1540), reduced to remove duplicates (1312), and manually reduced to a collection of the top 400 putative hits based on predicted affinity, assessed reliability of binding mode, and synthetic tractability. These top 400 results were extracted from the Maybridge fragment screening collection, loaded into PyMOL and underwent manual inspection.

3.2 Propensity Map Generation

The Cambridge Crystallographic Data Centre's (CCDC) SuperStar (2019 v.1) was used to predict cavities.[11] An *apo* structure of the PHD/BRD from TRIM33b was taken from the PDB file 5MR8. Cavity detection was performed over the entire protein at physiological pH, using the 'shallow/normal' cavity type, a grid spacing of 0.7 Å and a minimum cavity volume of 10 Å^3.

3.3 Molecular Dynamics Simulations

3.3.1 Protein and Peptide Parameterisation

The AMBER99sb-ILDN force field was used to parameterise the protein and peptide. Additional parameters required are described below.[12]

Parameterisation of Zinc Ions

The standard AMBER99sb-ILDN parameters for zinc can lead to unstable zinc coordination and poor reproduction of Zn–atom distances. To remedy this, zinc parameters developed by Procacci and co-workers were used.[13] This paper also introduced new residue types (CYZ/HDZ/HEZ) to represent zinc binding cysteine and histidine (HID/HIE) residues. The parameters of these new residue types better reflect the polarisation induced by the metal ion.

These parameters were incorporated into the AMBER99sb-ILDN force field, following the protocol described by Procacci and co-workers.

Parameterisation of Non-standard Amino Acids

Parameters for KAc and KMe_3 are not available in the AMBER99sb-ILDN force field. Instead, they were obtained from Forcefield_PTM, which introduces a new set of partial charges for KAc and KMe_3.[14] These parameters were originally developed for the ff03 force field.[15] In ff03, partial charges were calculated in the condensed phase, while ff99SB-ILDN is originally derived from ff94, where the charges were calculated in the gas phase. In the paper, it was shown that the charges on the modified amino acids generally correlated well with ff94, although there are some absolute differences. It was decided that it was an acceptable to use these modified parameters with the AMBER99sb-ILDN force field, and the parameters for KAc and KMe_3 were merged with Amber99sb-ILDN in tleap during peptide preparation in AmberTools20.

Protein and Peptide Preparation

Crystal structures of TRIM24, TRIM33α and TRIM33β were obtained from the Protein Data Bank (TRIM24: 3O33; TRIM33α: 3U5O; TRIM33β: 5MR8).[16,17] No crystal structures of TRIM24 and

TRIM33β in complex with the peptide of interest ($H3_{1-20}K9Me_3K18Ac$) were available. Thus, the peptide from the TRIM33α crystal structure (3U5O, chain I) was overlaid on the crystal structures of TRIM24 (PDB: 3O33, chain B) and TRIM33β (PDB: 5MR8), with the assumption that the peptide adopts a similar binding mode to all 3 proteins.

With TRIM24 and TRIM33β, 5 crystallographic waters at the bromodomain were retained during simulation setup. For TRIM33α, there were missing loop residues at both the interdomain linker and BC loop. Consequently, a homology model for this protein was generated using SWISS-MODEL to produce a model with no missing loop regions.[18,19] In this case, no crystallographic waters were present in the starting structure. In addition, in simulations of TRIM33α with the $H3_{1-20}K9Me_3K18Ac$ peptide, the first two residues of the TRIM33α model (D883, D884) were removed, such that the TRIM33α and TRIM33β structures both started from the same residue (P885). This was performed as D884 could potentially interact with the peptide. On the other hand, the C-terminus residues were on the opposite face of the protein, thus precluding any interactions with the peptide, so additional C-terminus residues were unlikely to affect peptide binding.

Molprobity was used to check for asparagine, glutamine and histidine flips, and to optimise hydrogen bonding networks.[20] The N and C-terminal residues of the proteins were capped with an acetyl and N-methyl group respectively. The proteins (TRIM24, TRIM33α and TRIM33β) were parameterised using the pdb2gmx tool in GROMACS 2019.4.[21]

For the H3 peptide, only the C-terminus was capped, and the peptide was parameterised using tleap in Ambertools20.[22] ACPYPE was subsequently used to convert the output files to GROMACS format.[23]

3.3.2 MD Simulations

All MD simulations were carried out using GROMACS 2019.2.[21]

The protein–peptide complex or protein was solvated with water (TIP3P)[24] in a rhombic dodecahedron box, with a minimum distance of 1.0 nm between the solute and the edge of the box. Then, the system was neutralised with Na^+ ions and the salt concentration adjusted to 150 mM NaCl.

Subsequently, energy minimisation using the steepest descend algorithm was performed. 5 independent MD simulations per system were initiated from this minimised structure, using random velocities derived from a Maxwell distribution at 300 K. 3 × 100 ps restrained NVT equilibration runs were performed, with position restraints set to 1000, 500 or 100 kJ mol^{-1} nm^{-2} respectively. The modified Berendsen thermostat[25] (velocity-rescale) was used for temperature coupling in these runs, and all subsequent runs. Following this, a 1 × 200 ps restrained NPT equilibration was performed, with position restraints set to 100 kJ mol^{-1} nm^{-2}, at a pressure of 1.0 bar. Pressure coupling was with the Berendsen barostat.[26] After equilibration, a 100 ns unrestrained MD simulation were performed, at 300 K and 1.0 bar. Pressure coupling was with the Parrinello-Rahman barostat.[27] 3D periodic boundary conditions were applied, and the Particle Mesh Ewald scheme used for calculating long-range electrostatics. All bonds involving hydrogen were constrained with the LINCS algorithm.[28]

A summary of these steps is presented in Table 22.

Table S22. Simulation setup parameters.

	EM	NVT-1	NVT-2	NVT-3	NPT	MD
Number of steps	Until $F_{max} < 1000$ kJ mol^{-1} nm^{-1}	50,000 per run			100,000	50,000,000
Step size	0.01 nm	2 fs				
Time (ps)	N/A	100 per run			200	100,000
Temperature (K)	0	300				
Pressure (bar)	N/A	N/A			1.0	
Random velocity generation	N/A	Yes	No			
Force constant for position restraints (kJ mol^{-1} nm^{-2})	N/A	1000	500	100		N/A

3.3.3 Data Analysis

Protein-Ligand Interaction Profiler

The Protein-Ligand Interaction Profiler (PLIP) software (v2.1.9)[29] was used for the detection of contacts made between the protein and peptide. Here, the PLIP command line tool (specifically, the Docker image) was used to enable batch processing in peptides mode.

Frames were extracted from each 100 ns trajectory at 1 ns intervals, to obtain 101 PDB files for analysis (505 PDB files for each protein, corresponding to 5 independent MD runs per system). Solvent was removed from these PDB files, and the files processed with PLIP in peptides mode. Default PLIP cut off values for hydrophobic interactions were used, while the hydrogen bond cut offs were adjusted to match the cut offs used with the VMD Hbonds tool (distance cut off of 3.5 Å, angle cut off of 150°, which corresponds to 30° in VMD).

Hydrogen Bonds/Salt bridges

Hydrogen bonds made between the peptide and protein were detected with the Hbonds tool in Visual Molecular Dynamics (VMD).[30] All frames from each trajectory were analysed, with a distance cut off of 3.5 Å and angle cut off of 30°. For acidic/basic residue pairs (e.g. H3R17–E981),

the occupancy of the acidic side chain–basic side chain hydrogen bond was used as a proxy for salt bridge occupancy.

Analysis of waters in the BRD pocket

In BRDs, a water network is present at the bottom of the binding pocket,[31] and these water molecules may contribute to stabilisation of K18Ac in the binding pocket. Examination of the crystal structures of TRIM24, TRIM33α and TRIM33β with the $H3_{1\text{–}20}K9Me_3K18Ac$ peptide overlaid indicated that there were up to five water molecules present at the base of the BRD pocket, with the possibility of a water-mediated hydrogen bond between the ZA loop tyrosine (Y935 in TRIM24, Y993 in TRIM33) and K18Ac (Figure S8).

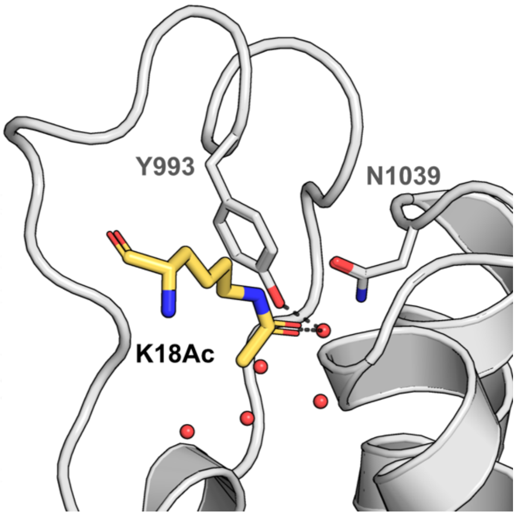

Figure S10. Possible water-mediated hydrogen bond (black dotted lines) between a ZA loop tyrosine and K18Ac, shown for the TRIM33β BRD (PDB: 5MR8 with chain I of PDB: 3U5O overlaid, protein in white, peptide in yellow). Crystallographic waters in the TRIM33β BRD pocket are shown as red spheres.

To identify regions of high water occupancy in the simulations, the density of water oxygen atoms (OW) in the simulation was calculated using the DensityAnalysis tool in MDAnalysis 2.1.0, with a bin size of 1.0 Å for the density grid. Frames for analysis taken from each trajectory at 200 ps intervals. The obtained density was converted to the density relative to the literature value of the

TIP3P water model. For the visualisation in PyMOL, the sigma level was set to 2.0 to identify regions with increased water occupancy relative to bulk TIP3P water.

Regions with high water occupancy during the MD simulations were compared against the waters present in crystal structures of TRIM24, TRIM33α and TRIM33β (Figure S11). In TRIM24 and TRIM33β, the water network was predicted to remain intact, with five water molecules indicated at the base of the pocket. In TRIM24, one of the five water molecules in the BRD pocket was not present in the crystal structure (compare TRIM24 against TRIM33α/β, Figure S11). Despite this, high water occupancy was predicted at this water position from the MD simulations, indicating that the waters inside the pocket could exchange with the bulk waters. In contrast, in TRIM33α the water network appeared to be disrupted. Only two out of five of the crystallographic waters were conserved during the MD simulation. Moreover, the water forming the putative water-mediated hydrogen bond between tyrosine and K18Ac appeared to have been displaced. This suggests that there is either minimal water-mediated hydrogen bonding between K18Ac and Y993, or that water-mediated hydrogen bonding between TRIM33α and K18Ac would occur *via* a different water molecule to that indicated in Figure S10.

While is possible that the TRIM33α simulations are affected by the lack of crystallographic waters in the homology model used, this seems unlikely considering that bulk water can easily enter the BRD pocket during the simulations, as noted in TRIM24.[32] Further investigations into the water networks of the two TRIM33 BRDs will be undertaken in the future to better understand the differences observed between TRIM33α and TRIM24/TRIM33β.[33]

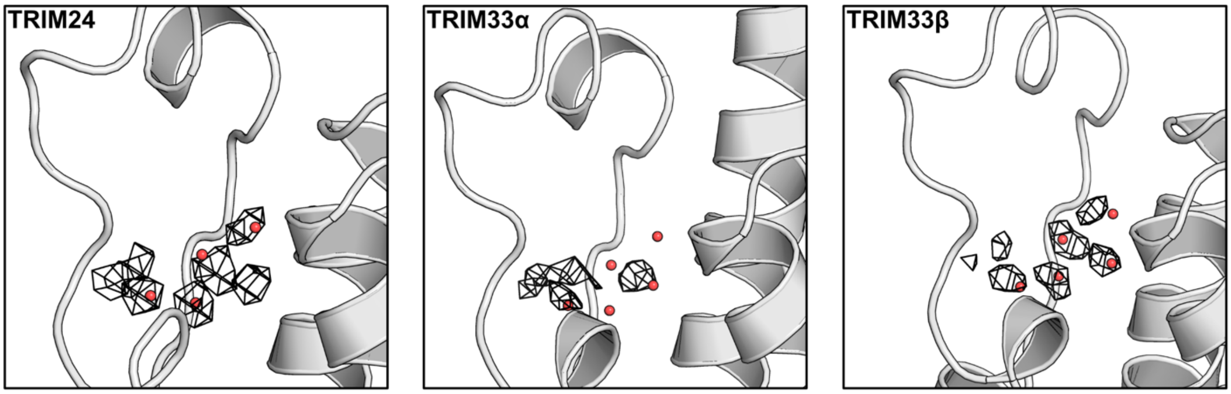

Figure S11. Regions of high water occupancy in the MD simulations of TRIM24, TRIM33α and TRIM33β complexed with $H3_{1\text{–}20}K9Me_3K18Ac$, compared against the crystallographic waters (red spheres). The crystallographic waters shown were taken from the following PDBs: 3O33 chain B for TRIM24, 3U5N chain B for TRIM33α, 5MR8 for TRIM33β. In the TRIM24 structure, only four of the five water molecules were present.

Further analysis of hydrophobic contacts

A further breakdown of the hydrophobic contacts between the H3 peptide and TRIM PHD-BRDs is provided in Tables S22–S28. Table S22 shows the aggregated PLIP analysis, while Tables S23-S28 show this analysis broken down for the individual peptide residues.

To probe whether F1038 fluctuates more when TRIM33α is complexed with an H3K18Ac-bearing peptide, additional MD simulations of apo TRIM24, TRIM33α and TRIM33β were performed (5 × 100 ns per system). In crystal structures of TRIM24, TRIM33α and TRIM33β, a side-on π-stacking interaction between Y935 and F979 (in TRIM24) or Y993 and F1038 (in TRIM33α/β) was observed. Measuring the distance between the side chains of these two residues during MD simulations showed that these two residues generally remain in close proximity to each other, both in the presence and absence of the H3K18Ac-bearing peptide (Figure S12). The exception to this was the peptide-bound form of TRIM33α, where greater fluctuations in the side chain distance were observed, beyond that seen in the apo form of the protein. This might indicate that F1038 in TRIM33α does indeed move to pack against H3K18Ac.

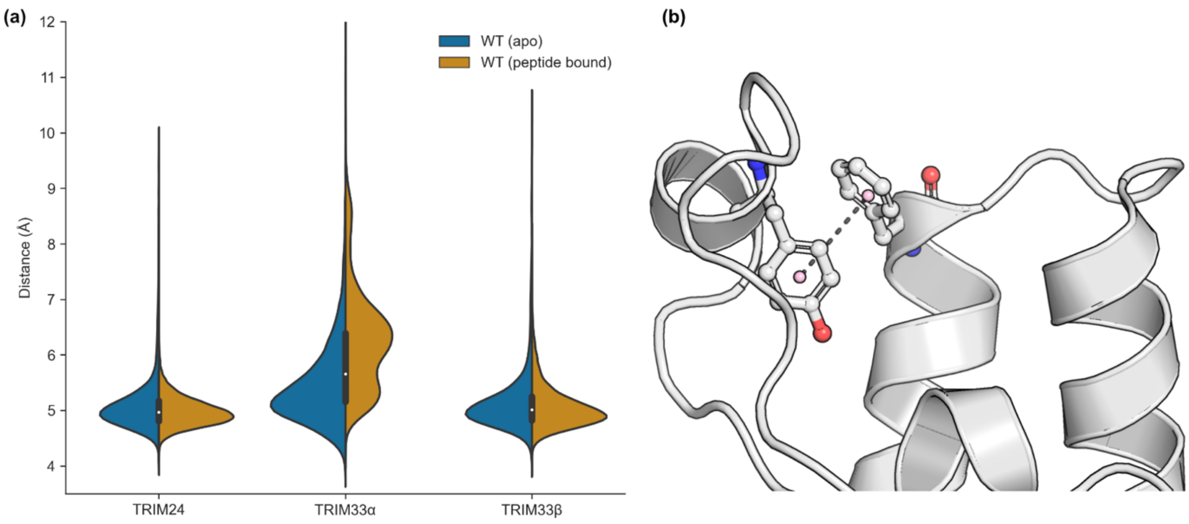

Figure S12. (a) Violin plots showing the distribution of distances between Y935–F979 (TRIM24) or Y993–F1038 (TRIM33α/β), as calculated from 5 × 100 ns MD simulations. The distance measured is between the centre of mass of the side chain of each amino acid residue, as shown for TRIM33β in (b).

Table S23. Total number of hydrophobic contacts made between peptide residues 15–20 and the TRIM proteins, as detected by PLIP (505 frames analysed).

Peptide residue	TRIM24	TRIM33α	TRIM33β
ALA15	242	423	308
PRO16	540	725	468
ARG17	38	30	25
ALY18	828	1572	799
GLN19	181	120	98
LEU20	146	524	215
Total	2085	3415	1983

Table S24. Hydrophobic contacts made between peptide residue A15 and specified residues of the TRIM proteins. Residue IDs are provided in the order TRIM24/TRIM33. When the residue IDs differ between TRIM33α and TRIM33β, the order then becomes TRIM24/TRIM33α/TRIM33β.

	**Peptide residue: A15**		
	**TRIM24**	**TRIM33α**	**TRIM33β**
E919/E977	6	4	14
M920/L978	2	124	45
L922/I980	193	294	171
A923/E981	8	1	71
E985/E1061/E1044	0	0	6
A989/A1065/A1048	33	0	1
Total	242	423	308

Table S25 Hydrophobic contacts made between peptide residue P16 and specified residues of the TRIM proteins. Residue IDs are provided in the order TRIM24/TRIM33. When the residue IDs differ between TRIM33α and TRIM33β, the order then becomes TRIM24/TRIM33α/TRIM33β.

	**Peptide residue: P16**		
	**TRIM24**	**TRIM33α**	**TRIM33β**
E919/E977	0	0	7
M920/L978	15	49	40
L922/I980	23	2	6
A923/E981	64	1	25
F924/F982	1	0	9
T1051 (TRIM33α)	N/A	11	N/A
E985/E1061/E1044	144	345	105
V986/V1062/V1045	129	67	79
A989/A1065/A1048	164	250	197
Total	540	725	468

Table S26. Hydrophobic contacts made between peptide residue R17 and specified residues of the TRIM proteins. Residue IDs are provided in the order TRIM24/TRIM33. When the residue IDs differ between TRIM33α and TRIM33β, the order then becomes TRIM24/TRIM33α/TRIM33β.

	**Peptide residue: R17**		
	**TRIM24**	**TRIM33α**	**TRIM33β**
L922/I980	16	16	2
A923/E981	2	14	6
E985/E1061/E1044	18	0	12
V986/V1062/V1045	2	0	5
Total	38	30	25

Table S27. Hydrophobic contacts made between peptide residue K18Ac and specified residues of the TRIM proteins. Residue IDs are provided in the order TRIM24/TRIM33. When the residue IDs differ between TRIM33α and TRIM33β, the order then becomes TRIM24/TRIM33α/TRIM33β.

	**Peptide: K18Ac**		
	**TRIM24**	**TRIM33α**	**TRIM33β**
M920/L978	0	0	6
A923/E981	44	217	118
F924/F982	117	188	81
V928/V986	271	101	159
P929/P987	0	1	3
V932/I990	65	122	109
P933/P991	0	0	9
Y935/Y993	112	115	59
I939/I997	0	3	6
I972/I1031	0	22	12
N975/N1034	0	11	4
F979/F1038	100	547	104
N980/N1039	0	1	0
E981/E1040	0	0	3
V986/V1062/V1045	119	240	104
A987/A1063/A1046	0	4	0
A989/A1065/A1048	0	0	20
Total	828	1572	797

Table S28. Hydrophobic contacts made between peptide residue Q19 and specified residues of the TRIM proteins. Residue IDs are provided in the order TRIM24/TRIM33. When the residue IDs differ between TRIM33α and TRIM33β, the order then becomes TRIM24/TRIM33α/TRIM33β.

	**Peptide residue: Q19**		
	**TRIM24**	**TRIM33α**	**TRIM33β**
V928/V986	0	16	2
P929/P987	77	25	8
T931/S989	1	0	0
V932/I990	86	60	53
P933/P991	3	1	0
F979/F1038	1	2	13
N980/N1039	0	0	4
E981/E1040	13	0	17
Q1046 (TRIM33α)	N/A	2	N/A
V1047 (TRIM33α)	N/A	1	N/A
Y1048 (TRIM33α)	N/A	3	N/A
A1049 (TRIM33α)	N/A	1	N/A
T1051 (TRIM33α)	N/A	6	N/A
I1054 (TRIM33α)	N/A	3	N/A
D983/D1059/D1042	0	0	1
Total	181	120	98

Table S29. Hydrophobic contacts made between peptide residue L20 and specified residues of the TRIM proteins. Residue IDs are provided in the order TRIM24/TRIM33. When the residue IDs differ between TRIM33α and TRIM33β, the order then becomes TRIM24/TRIM33α/TRIM33β.

	**Peptide residue: L20**		
	**TRIM24**	**TRIM33α**	**TRIM33β**
L922/I980	24	0	0
A923/E981	24	0	0
D926/E984	4	0	0
V928/V986	9	42	0
P929/P987	6	2	4
T931/S989	2	0	0
V932/I990	33	94	27
P933/P991	29	36	24
D934/N992	0	0	1
Y935/Y993	0	45	0
I938/I996	0	1	3
F979/F1038	9	68	83
N980/N1039	0	0	1
E981/E1040	5	0	5
M1042 (TRIM33α)	N/A	4	N/A
Q1046 (TRIM33α)	N/A	10	N/A
Y1048 (TRIM33α)	N/A	34	N/A
A1049 (TRIM33α)	N/A	32	N/A
T1051 (TRIM33α)	N/A	13	N/A
Q1052 (TRIM33α)	N/A	17	N/A
I1054 (TRIM33α)	N/A	15	N/A
N1055 (TRIM33α)	N/A	4	N/A
L1056 (TRIM33α)	N/A	11	N/A
K1057 (TRIM33α)	N/A	6	N/A
P982/A1058/A1041	0	2	0
D983/D1059/D1042	0	0	4
E985/E1061/E1044	1	0	26
V986/V1062/V1045	0	88	37
Total	146	524	215

Table S30: Crystallographic data collection and refinement statistics.

[a] Highest resolution shell (in Å) shown in parentheses.

PROTEIN ID	TRIM33B (PHD-BRD)	
**Ligand**	H3K9Ac	H3K10Ac
**Data collection**		
Space Group	P 21 21 21	P 21 21 21
Cell Dimensions		
a,b,c (Å)	50.88 57.99 71.49	36.13 52.89 105.70
α, β, γ (º)	90.00 90.00 90.0	90.0 90.0 90.0
Resolution (Å)	29.00 (1.74)[a]	37.39 – 1.62 (1.78 – 1.62)
Unique Observations	22066 (1164)[a]	16564 (829)
Completeness (%)	97.8 (29.00-1.74)	92.2 (57.7)
Redundancy	5.8 (5.1)[a]	6.2 (7.3)
R_{sym} or R_{merge}	0.035 (0.678)[a]	0.098 (1.184)
$I/\sigma I$	19.7 (2.1)[a]	12.0 (1.6)
Wavelength	0.9763	0.998 (0.595)
Phasing	MR	MR
**Refinement**		
R_{work} / R_{free} (%)	22.7 / 28.6	20.1 / 24.3
Wilson B-factor (Å^2)	32.7	20.4
Total number of atoms	1626	1493 / 24 / 182 (protein / water / other)
Average B, all atoms (Å^2)	58.0	23.2 / 27.1 / 34.8 / 31.6 (protein/peptide/water/other)
R.M.S.D. Bond (Å)	0.005	0.008
R.M.S.D. Angle (º)	0.792	0.860
Ramachandran outliers	0.5%	0%
Sidechain outliers	1.7%	1.8%
**PDB ID**	5MR8	7ZDD

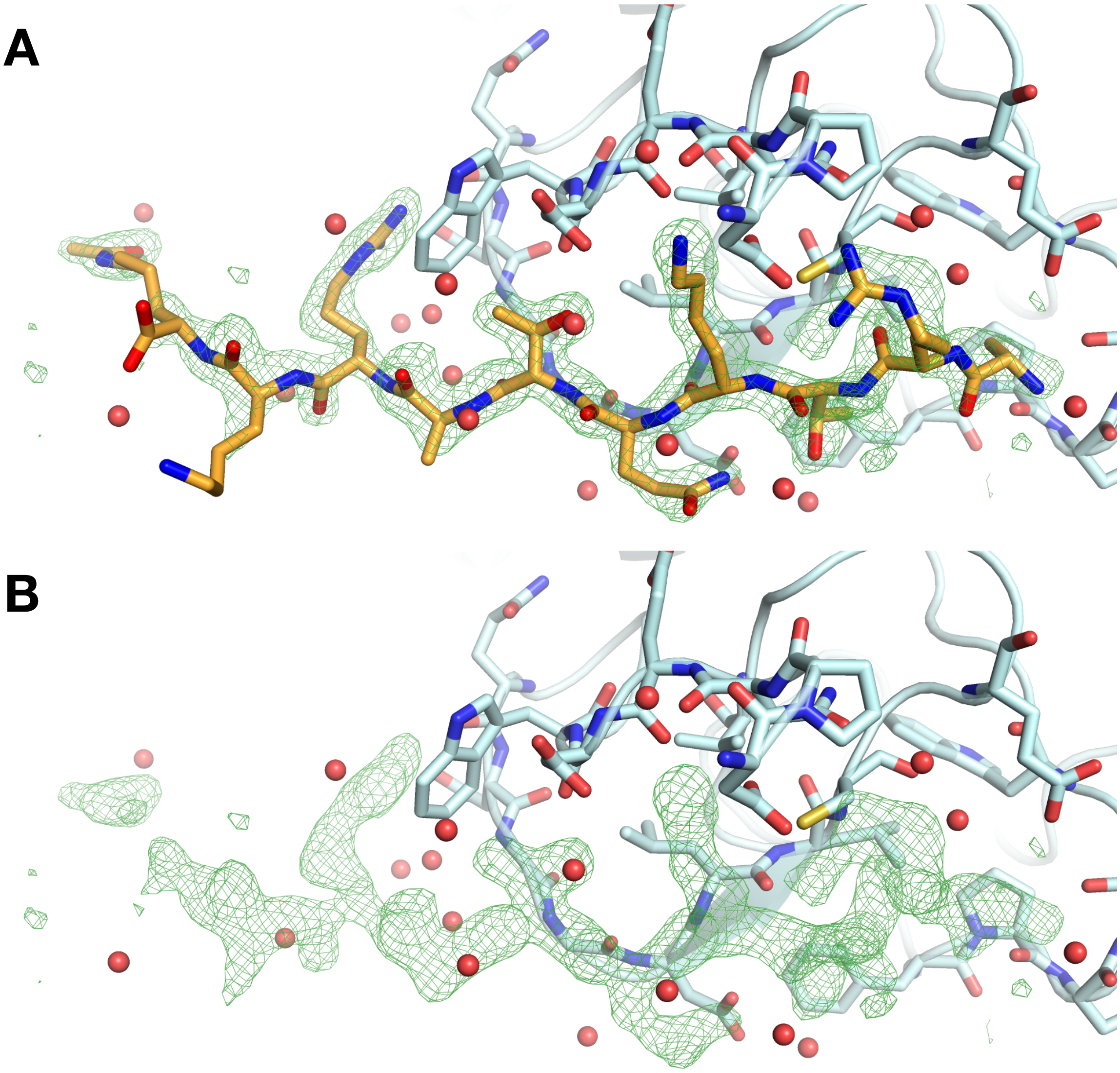

Figure S13A. Omit map (model refined without peptide) superimposed with final model. TRIM33β carbon = blue; $H3_{1\text{-}10}K10Ac$ carbon = orange. Fo-Fc map sigma level of 3. **B**. Omit map (model refined without peptide) superimposed with final model without peptide. TRIM33β carbon = blue. Fo-Fc map sigma level of 3.

4 NMR spectra and HPLC trace for compound 8

^{1}H NMR: 4’-(2’’-{[(1,3-Dimethyl-2-oxo-2,3-dihydro-1*H*-benzimidazol-5-yl)methyl]amino}ethyl)benzene-1’-sulfonamide (**8**)

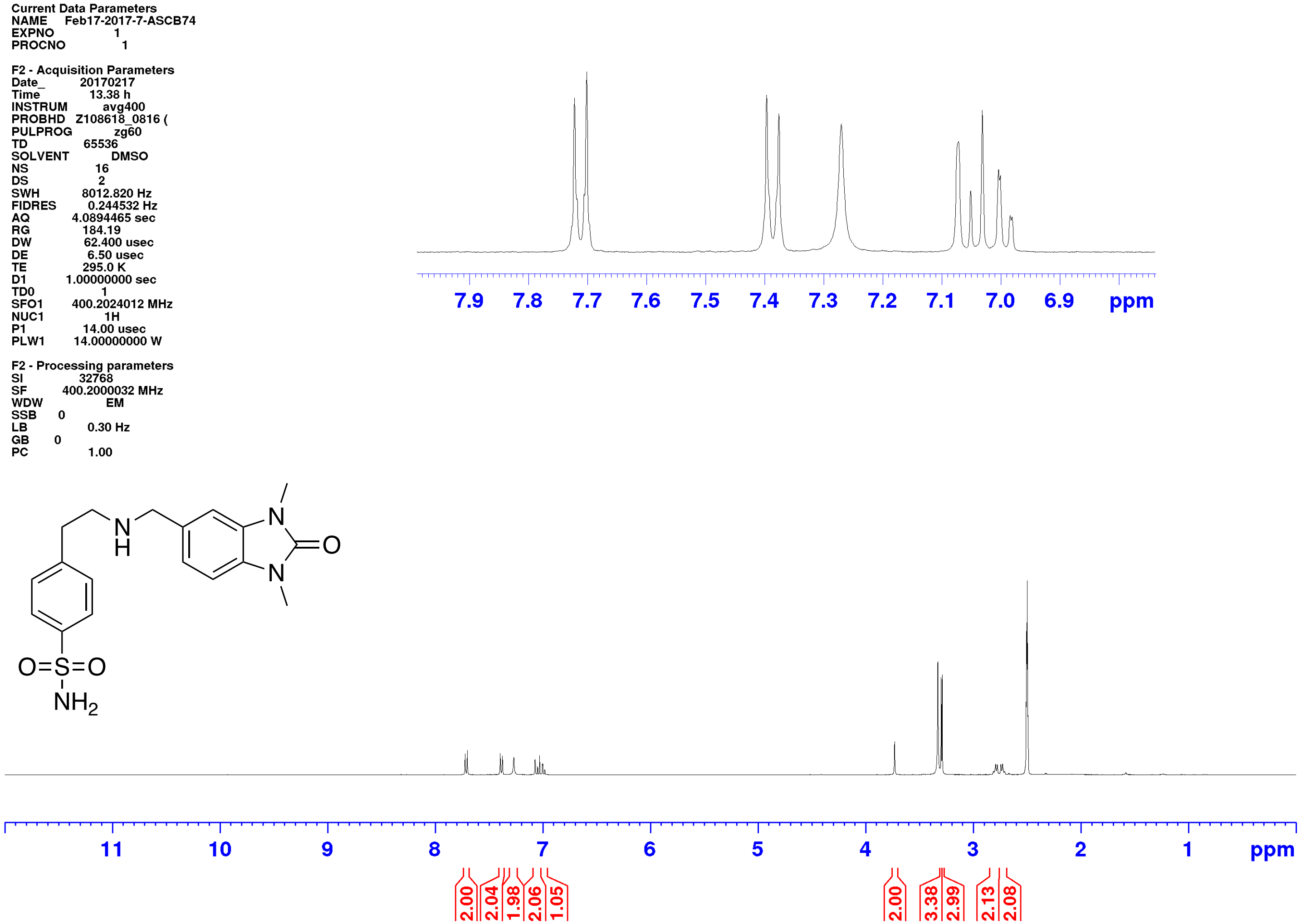

^{13}C NMR: 4’-(2’’-{[(1,3-Dimethyl-2-oxo-2,3-dihydro-1*H*-benzimidazol-5-yl)methyl]amino}ethyl)benzene-1’-sulfonamide (**8**)

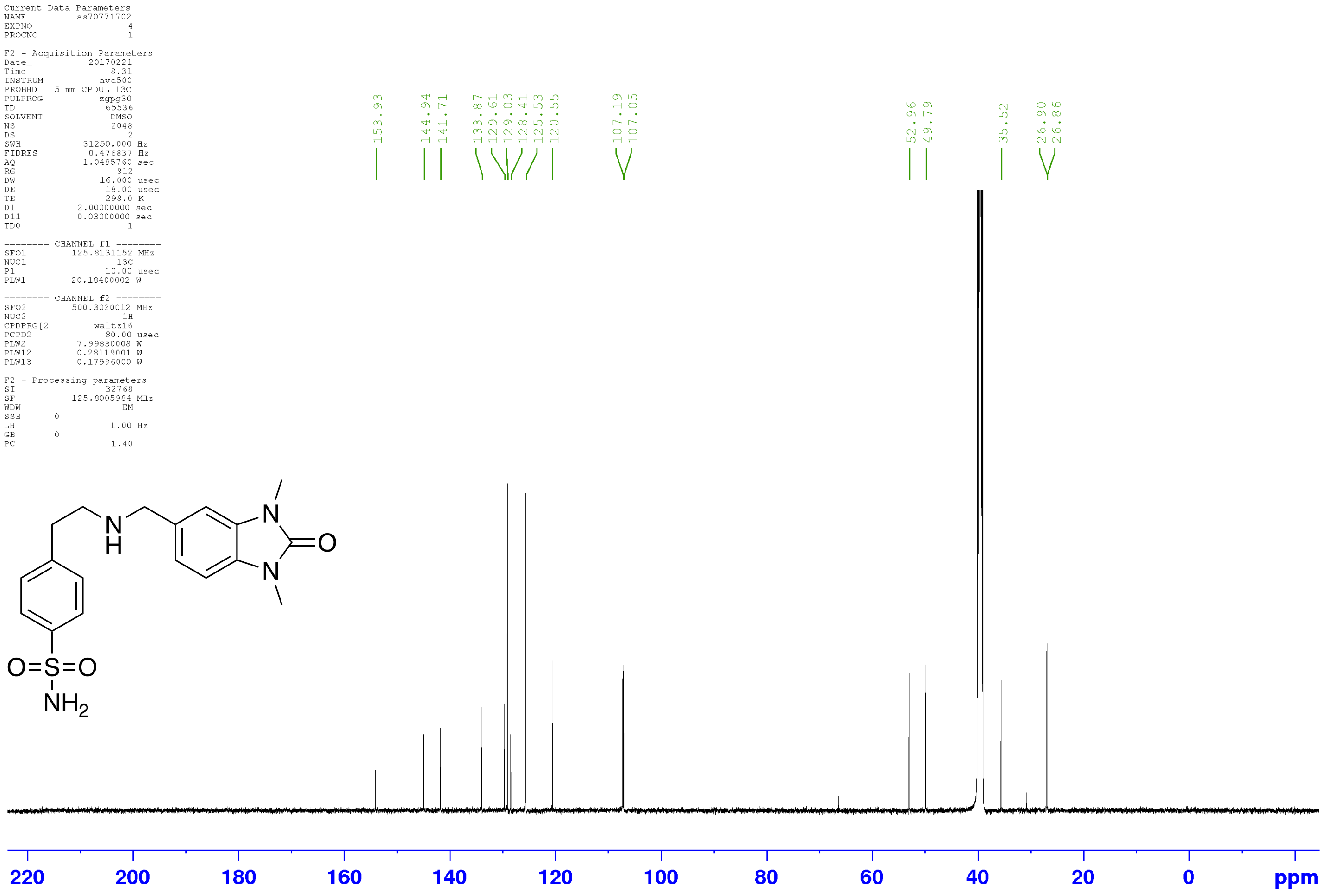

HPLC: 4’-(2’’-{[(1,3-Dimethyl-2-oxo-2,3-dihydro-1*H*-benzimidazol-5-yl)methyl]amino}ethyl)benzene-1’-sulfonamide (**8**)

ASCB74 **Purity short run @254 nm** **2/27/2017 4:09 pm**

Sample Name	ASCB74
Vial Number	2
Injection Volume	15
Acquisition Date/Time	2/27/2017 3:14 pm
Acquisition Method	Purity short run @254 nm
Processing Method	Purity short run @254 nm

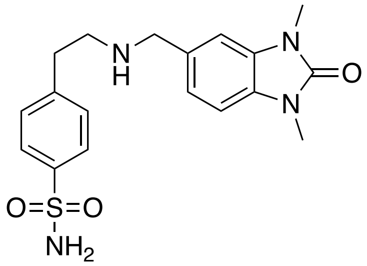

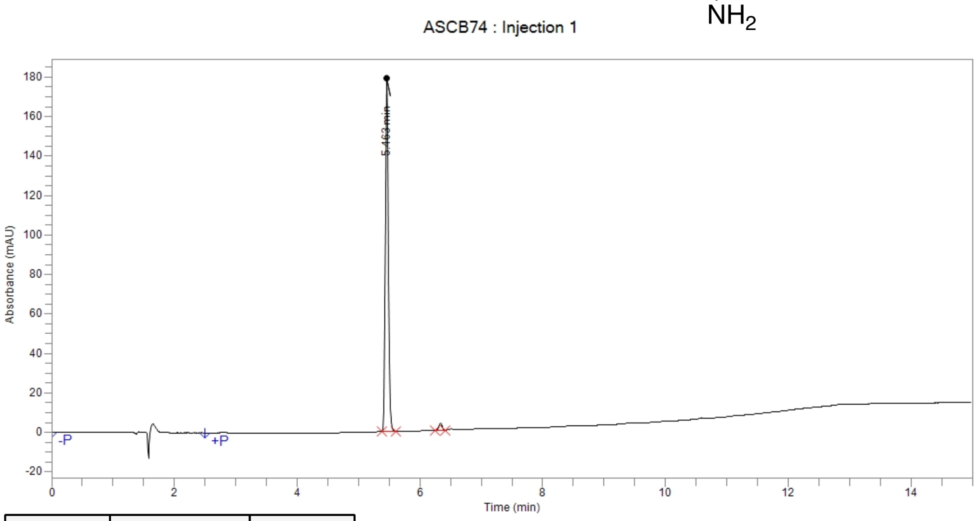

Time	Area	Area %
5.463	598,860.3	97.74
6.339	13,868.4	2.26
**Total**	612,728.6	100.00

5 References

(1) Gibson, D. G.; Young, L.; Chuang, R.; Venter, J. C.; Hutchison, C. A; Smith, H. O. Enzymatic Assembly of DNA Molecules up to Several Hundred Kilobases. *Nat. Methods* **2009**, *6*, 343–345. DOI: 10.1038/nmeth.1318.

(2) Leatherbarrow, R. J. *GraFit Version 7*; Erithacus Software Ltd. Horley, U.K., 2009.

(3) Watson, V. G.; Drake, K. M.; Peng, Y.; Napper, A. D. Development of a High-Throughput Screening-Compatible Assay for the Discovery of Inhibitors of the Af4-Af9 Interaction Using Alphascreen Technology. *Assay and Drug Development Technologies* **2013**, *11*, 253–268. DOI: 10.1089/ADT.2012.495/ASSET/IMAGES/LARGE/FIGURE11.JPEG.

(4) Dalvit, C. Efficient Multiple-Solvent Suppression for the Study of the Interactions of Organic Solvents with Biomolecules. *J. Biomol. NMR* **1998**, *11*, 437–444. DOI: 10.1023/A1008272928075.

(5) Fulmer, G. R.; Miller, A. J.; Sherden, N. H.; Gottlieb, H. E.; Nudelman, A.; Stoltz, B. M.; Bercaw, J. E.; Goldberg, K. I.; Beckman, M. NMR Chemical Shifts of Trace Impurities: Common Laboratory Solvents, Organics, and Gases in Deuterated Solvents Relevant to the Organometallic Chemist. *Organometallics* **2010**, *29*, 2176–2179. DOI: 10.1021/om100106e.

(6) Hewings, D. S.; Fedorov, O.; Filippakopoulos, P.; Martin, S.; Picaud, S.; Tumber, A.; Wells, C.; Olcina, M. M.; Freeman, K.; Gill, A. *et al.* Optimization of 3,5-Dimethylisoxazole Derivatives as Potent Bromodomain Ligands. *J. Med. Chem.* **2013**, *56*, 3217–3227. DOI: 10.1021/jm301588r.

(7) Philpott, M.; Yang, J.; Tumber, T.; Fedorov, O.; Uttarkar, S.; Filippakopoulos, P.; Picaud, S.; Keates, T.; Felletar, I.; Ciulli, A. *et al.* Bromodomain-Peptide Displacement Assays for Interactome Mapping and Inhibitor Discovery. *MolBioSyst.* **2011**, *7*, 2899–2908. DOI: 10.1039/c1mb05099k.

(8) Ross, G. A.; Morris, G. M.; Biggin, P. C. Rapid and Accurate Prediction and Scoring of Water Molecules in Protein Binding Sites. *PloS ONE* **2012**, *7*, e32036. DOI: 10.1371/journal.pone.0032036.

(9) Morris, G. M.; Huey, R.; Lindstrom, W.; Sanner, M. F.; Belew, R. K.; Goodsell, D. S.; Olson, A. J. AutoDock4 and AutoDockTools4: Automated Docking with Selective Receptor Flexibility. *J. Comp. Chem.* **2009**, *31*, 2967–2970. DOI: 10.1002/jcc.

(10) Trott, O.; Olson, A. J. AutoDock Vina : Improving the Speed and Accuracy of Docking with a New Scoring Function , Efficient Optimization , and Multithreading. *J. Comp. Chem.* **2010**, *31*, 455–461. DOI: 10.1002/jcc.

(11) Verdonk, M. L.; Cole, J. C.; Watson, P.; Gillet, V.; Willett, P. SuperStar: Improved Knowledge-Based Interaction Fields for Protein Binding Sites. *J. Mol. Biol.* **2001**, *307*, 841–859. DOI: 10.1006/jmbi.2001.4452.

(12) Lindorff-Larsen, K.; Piana, S.; Palmo, K.; Maragakis, P.; Klepeis, J. L.; Dror, R. O.; Shaw, D. E. Improved Side-chain Torsion Potentials for the Amber Ff99SB Protein Force Field.

Proteins: Structure, Function, and Bioinformatics **2010**, *78*, 1950–1958. DOI: 10.1002/prot.22711.

(13) Macchiagodena, M.; Pagliai, M.; Andreini, C.; Rosato, A.; Procacci, P. Upgrading and Validation of the AMBER Force Field for Histidine and Cysteine Zinc(II)-Binding Residues in Sites with Four Protein Ligands. *J. Chem. Inf. Model.* **2019**, *59*, 3803–3816. DOI: 10.1021/acs.jcim.9b00407.

(14) Khoury, G. A.; Thompson, J. P.; Smadbeck, J.; Kieslich, C. A.; Floudas, C. A. Forcefield_PTM: Ab Initio Charge and AMBER Forcefield Parameters for Frequently Occurring Post-Translational Modifications. *J. Chem. Theory Comp.* **2013**, *9*, 5653–5674. DOI: 10.1021/ct400556v.

(15) Duan, Y.; Wu, C.; Chowdhury, S.; Lee, M. C.; Xiong, G.; Zhang, W.; Yang, R.; Cieplak, P.; Luo, R.; Lee, T. *et al.* Point-charge Force Field for Molecular Mechanics Simulations of Proteins Based on Condensed-phase Quantum Mechanical Calculations. *J. Comp. Chem.* **2003**, *24*, 1999–2012. DOI: 10.1002/jcc.10349.

(16) Xi, Q.; Wang, Z.; Zaromytidou, A. I.; Zhang, X. H. F.; Chow-Tsang, L. F.; Liu, J. X.; Kim, H.; Barlas, A.; Manova-Todorova, K.; Kaartinen, V. *et al.* Poised Chromatin Platform for TGF-Beta Access to Master Regulators. *Cell* **2011**, *147*, 1511–1524. DOI: 10.1016/j.cell.2011.11.032.

(17) Tsai, W. W.; Wang, Z.; Yiu, T. T.; Akdemir, K. C.; Xia, W.; Winter, S.; Tsai, C.-Y.; Shi, X.; Schwarzer, D.; Plunkett, W. *et al.* TRIM24 Links a Non-Canonical Histone Signature to Breast Cancer. *Nature* **2010**, *468*, 927–932. DOI: 10.1038/nature09542.

(18) Biasini, M.; Bienert, S.; Waterhouse, A.; Arnold, K.; Studer, G.; Schmidt, T.; Kiefer, F.; Cassarino, T. G.; Bertoni, M.; Bordoli, L. *et al.* SWISS-MODEL: Modelling Protein Tertiary and Quaternary Structure Using Evolutionary Information. *Nucleic Acids Res.* **2014**, *42*, W252–W258. DOI: 10.1093/nar/gku340 PMID - 24782522.

(19) Waterhouse, A.; Bertoni, M.; Bienert, S.; Studer, G.; Tauriello, G.; Gumienny, R.; Heer, F. T.; de Beer, T. A. P.; Rempfer, C.; Bordoli, L. *et al.* SWISS-MODEL: Homology Modelling of Protein Structures and Complexes. *Nucleic Acids Res.* **2018**, *46*, gky427. DOI: 10.1093/nar/gky427.

(20) Williams, C. J.; Headd, J. J.; Moriarty, N. W.; Prisant, M. G.; Videau, L. L.; Deis, L. N.; Verma, V.; Keedy, D. A.; Hintze, B. J.; Chen, V. B. *et al.* MolProbity: More and Better Reference Data for Improved All-atom Structure Validation. *Protein Science* **2018**, *27*, 293–315. DOI: 10.1002/pro.3330.

(21) Abraham, M. J.; Murtola, T.; Schulz, R.; Páll, S.; Smith, J. C.; Hess, B.; Lindahl, E. GROMACS: High Performance Molecular Simulations through Multi-Level Parallelism from Laptops to Supercomputers. *SoftwareX* **2015**, *1*, 19–25. DOI: 10.1016/j.softx.2015.06.001.

(22) Case, D. A.; Belfon, K.; Ben-Shalom, I. Y.; Brozell, S. R.; Cerutti, D. S.; Cheatham, T. E.; III; Cruzeiro, V. W. D.; Darden, T. A.; Duke, R. E.; Giambasu, G. *et al.* AMBER 2020. University of California, San Francisco 2020.

(23) Silva, A. W. S. da; Vranken, W. F. ACPYPE - AnteChamber PYthon Parser InterfacE. *BMC Res. Notes* **2012**, *5*, 367. DOI: 10.1186/1756-0500-5-367.

(24) Jorgensen, W. L.; Chandrasekhar, J.; Madura, J. D.; Impey, R. W.; Klein, M. L. Comparison of Simple Potential Functions for Simulating Liquid Water. *J. Chem. Physics* **1983**, *79*, 926–935. DOI: 10.1063/1.445869.

(25) Bussi, G.; Donadio, D.; Parrinello, M. Canonical Sampling through Velocity Rescaling. *The J. Chem. Physics* **2007**, *126*, 14101. DOI: 10.1063/1.2408420 PMID - 17212484.

(26) Berendsen, H. J. C.; Postma, J. P. M.; van Gunsteren, W. F.; DiNola, A.; Haak, J. R. Molecular Dynamics with Coupling to an External Bath. *Phys Rev. Lett.* **1984**, *81*, 3684–3690. DOI: 10.1063/1.436761.

(27) Parrinello, M.; Rahman, A. Polymorphic Transitions in Single Crystals: A New Molecular Dynamics Method. *J. Applied Phy.* **1981**, *52*, 7182–7190. DOI: 10.1063/1.328693.

(28) Hess, B.; Bekker, H.; Berendsen, H. J. C.; Fraaije, J. G. E. M. LINCS: A Linear Constraint Solver for Molecular Simulations. *J. Comp. Chem.* **1997**, *18*, 1463–1472. DOI10.1002/(sici)1096-987x(199709)18:12<1463::aid-jcc4>3.0.co;2-h.

(29) Salentin, S.; Schreiber, S.; Haupt, V. J.; Adasme, M. F.; Schroeder, M. PLIP: Fully Automated Protein–Ligand Interaction Profiler. *Nucleic Acids Research* **2015**, *43*, W443–W447. DOI: 10.1093/nar/gkv315.

(30) Humphrey, W.; Dalke, A.; Schulten, K. VMD: Visual Molecular Dynamics. *J. Mol. Graph.* **1996**, *14*, 33–38. DOI: 10.1016/0263-7855(96)00018-5.

(31) Owen, D. J.; Ornaghi, P.; Yang, J. C.; Lowe, N.; Evans, P. R.; Ballario, P.; Neuhaus, D.; Filetici, P.; Travers, A. A. The Structural Basis for the Recognition of Acetylated Histone H4 by the Bromodomain of Histone Acetyltransferase Gcn5p. *EMBO J.* **2000**, *19*, 6141–6149. DOI: 10.1093/EMBOJ/19.22.6141.

(32) Huang, D.; Rossini, E.; Steiner, S.; Caflisch, A. Structured Water Molecules in the Binding Site of Bromodomains Can Be Displaced by Cosolvent. *ChemMedChem* **2014**, *9*, 573–579. DOI: 10.1002/cmdc.201300156.

(33) Aldeghi, M.; Ross, G. A.; Bodkin, M. J.; Essex, J. W.; Knapp, S.; Biggin, P. C. Large-Scale Analysis of Water Stability in Bromodomain Binding Pockets with Grand Canonical Monte Carlo. *Commun. Chem.* **2018**, *1*, 19. DOI: 10.1038/s42004-018-0019-x.
